# FOXJ1 transcriptional targets in human airway cells and impaired multiciliogenesis in *FOXJ1*-associated primary ciliary dyskinesia

**DOI:** 10.1093/ajrcmb/aanag071

**Published:** 2026-03-30

**Authors:** Lucie Thomas, Jacques Serizay, Rahma Mani, Sandrine Couvet, Jean-François Papon, Aline Tamalet, Rana Mitri, Catherine Faucon, Marlene Murris-Espin, Emmanuelle Blanchard, Maud Langeois, Sophie Blesson, Sylvie Tissier, Guy Montantin, Florence Dastot-Le Moal, Romain Levergeois, Bruno Louis, Nicole Beydon, Guillaume Thouvenin, Estelle Escudier, Serge Amselem, Irina Giurgea, Laure-Emmanuelle Zaragosi, Alice Meunier, Marie Legendre

**Affiliations:** INSERM U933, Childhood Genetic Diseases, Sorbonne Université, Hôpital Armand-Trousseau, Paris, France; Institut Pasteur, CNRS UMR 3525, Université Paris Cité, Unité Régulation Spatiale des Génomes, Paris, France; Institut de biologie de l‘Ecole normale supérieure (IBENS), Ecole normale supérieure, CNRS, INSERM, Université PSL, Paris, France; AP-HP, UF de Génétique Moléculaire, Sorbonne Université, Hôpital Armand-Trousseau, Paris, France; INSERM U933, Childhood Genetic Diseases, Sorbonne Université, Hôpital Armand-Trousseau, Paris, France; INSERM U933, Childhood Genetic Diseases, Sorbonne Université, Hôpital Armand-Trousseau, Paris, France; AP-HP, Service d’Oto-Rhino-Laryngologie et de Chirurgie Cervico-Faciale, Université Paris-Saclay, Hôpital Bicêtre, Le Kremlin–Bicêtre, France; Service de Pneumologie Pédiatrique, Hôpital Trousseau, AP-HP, Sorbonne Université, INSERM, Centre de Recherche Saint-Antoine, CRSA, Paris, France; Service d‘Anatomopathologie, Laboratoire de Microscopie Electronique, Centre Hospitalier Intercommunal de Créteil, Créteil, France; Service d‘Anatomopathologie, Laboratoire de Microscopie Electronique, Centre Hospitalier Intercommunal de Créteil, Créteil, France; Service de Pneumologie, CRCM adulte et Transplantation pulmonaire, Clinique des Voies Respiratoires, CHU de Toulouse, Hôpital Larrey, Toulouse, France; Service d’Anatomie et Cytologie Pathologiques, Hôpital Bretonneau & INSERM US61 ASB, Université de Tours & CHU de Tours, Tours, France; Service de Génétique Médicale, Hôpital Purpan, CHU Toulouse, Toulouse, France; Service de Génétique, Hôpital Bretonneau, CHU Tours, Tours, France; Pédiatrie du Développement, PCO TND 0-12, PEP32 Auch, Gers, France; AP-HP, UF de Génétique Moléculaire, Sorbonne Université, Hôpital Armand-Trousseau, Paris, France; AP-HP, UF de Génétique Moléculaire, Sorbonne Université, Hôpital Armand-Trousseau, Paris, France; AP-HP, UF de Génétique Moléculaire, Sorbonne Université, Hôpital Armand-Trousseau, Paris, France; AP-HP, UF de Génétique Moléculaire, Sorbonne Université, Hôpital Armand-Trousseau, Paris, France; Université Paris-Est Créteil, Faculté de Médecine, INSERM U955, CNRS ERL7240, Hôpital Henri-Mondor, Créteil, France; AP-HP, Unité Fonctionnelle d‘Exploration Fonctionnelle Respiratoire–Somnologie, Service de Physiologie et Explorations Fonctionnelles, Centre National de Référence des Maladies Respiratoires Rares RespiRare, Hôpital Armand-Trousseau, Paris, France; Service de Pneumologie Pédiatrique, Hôpital Trousseau, AP-HP, Sorbonne Université, INSERM, Centre de Recherche Saint-Antoine, CRSA, Paris, France; INSERM U933, Childhood Genetic Diseases, Sorbonne Université, Hôpital Armand-Trousseau, Paris, France; AP-HP, UF de Génétique Moléculaire, Sorbonne Université, Hôpital Armand-Trousseau, Paris, France; INSERM U933, Childhood Genetic Diseases, Sorbonne Université, Hôpital Armand-Trousseau, Paris, France; AP-HP, UF de Génétique Moléculaire, Sorbonne Université, Hôpital Armand-Trousseau, Paris, France; INSERM U933, Childhood Genetic Diseases, Sorbonne Université, Hôpital Armand-Trousseau, Paris, France; AP-HP, UF de Génétique Moléculaire, Sorbonne Université, Hôpital Armand-Trousseau, Paris, France; Université Côte d‘Azur, CNRS, Institut Pharmacologie Moléculaire et Cellulaire, Sophia-Antipolis, France; Institut de biologie de l‘Ecole normale supérieure (IBENS), Ecole normale supérieure, CNRS, INSERM, Université PSL, Paris, France; INSERM U933, Childhood Genetic Diseases, Sorbonne Université, Hôpital Armand-Trousseau, Paris, France; AP-HP, UF de Génétique Moléculaire, Sorbonne Université, Hôpital Armand-Trousseau, Paris, France

**Keywords:** multiciliogenesis, FOXJ1, primary ciliary dyskinesia, transcription factor, ChIP-sequencing

## Abstract

Fluid mobilization on epithelia is ensured by the motile cilia of differentiated multiciliated cells (MCCs). Key transcriptional regulators of motile ciliogenesis include CCNO, MCIDAS, RFXs, and the transcription factor FOXJ1, whose precise role in humans remains unclear. We show that, unlike CCNO and MCIDAS, FOXJ1 expression persists in well-differentiated human airway epithelial cells (hAECs), suggesting functions beyond cilia initiation. Chromatin-immunoprecipitation sequencing in human airway epithelial cells allowed us to refine the consensus target motifs of FOXJ1 and RFXs, as well as their close proximity, which strongly suggests functional cooperation within a transcriptional complex. By combining chromatin-immunoprecipitation sequencing in normal cells with RNAseq from patients with *FOXJ1*-related primary ciliary dyskinesia, we identified 683 direct FOXJ1 target genes. Among these, 89 MCC-enriched genes—particularly those encoding axonemal proteins such as microtubule-inner proteins and dynein arm docking components—were downregulated in FOXJ1-deficient cells. Collectively, these findings provide new insights into how FOXJ1 contributes to human motile ciliogenesis and reveal a potential role for FOXJ1 in maintaining MCCs differentiation and ciliary function by sustaining the expression of ciliary proteins, particularly those in direct contact with axonemal tubulin.

## Introduction

Multiciliated cells (MCCs) are highly specialized epithelial cells that generate directional biological fluid flow across epithelial surface through the movement of hundreds of motile cilia. They are abundant in the airways, oviducts, efferent ducts, and ependymal epithelia, where their activity is essential for mucociliary clearance, gamete transport, and cerebrospinal fluid circulation.^1^ Motile cilia, like immotile primary (sensory) cilia, are evolutionarily conserved organelles based on a microtubule core, the axoneme, which extends from basal bodies derived from centrioles during ciliogenesis. In motile cilia, outer dynein arms (ODAs) and inner dynein arms (IDAs)—multiprotein complexes carrying an ATPase activity—are preassembled in the cytoplasm by axonemal dynein assembly factors (DNAAFs) complexes before being attached along the axonemal microtubules to provide the sliding force for motility.^2^

In the airway epithelium, basal progenitor cells can differentiate into MCCs or secretory cells. Motile ciliogenesis requires the repression of the Notch signalling pathway and the coordinated action of multiple transcription factors,^3^^,^^4^ including MCIDAS (Multicilin), CCNO (Cyclin O), RFX2/3 (Regulatory Factor binding to the X-box 2 and 3), and the master regulator FOXJ1 (Forkhead Box J1).^1^ Although RFX transcription factors have been shown to play a role in both primary and motile ciliogenesis,^5^ CCNO, MCIDAS, and FOXJ1 are exclusively implicated in motile cilia formation. The process of MCC differentiation has been delineated into distinct stages. During the initial stage, the elevated expression of MCIDAS and GMN1 (Geminin coiled-coil-domain-containing-protein-1) enables the activation of genes involved in centriole formation such as CCNO.^6^ The following stage is then characterized by the biosynthesis of basal bodies. The final stage involves FOXJ1 and RFX2/3, which are activated by GMN1, MCIDAS, and TP73 and promote the docking of basal bodies at the apical membrane and the generation of motile cilia.^7^ Although interactions between FOXJ1 and RFX transcription factors have been well documented in several models such as *Drosophila*,^8^^,^^9^  *Caenorhabditis elegans*,^10^ or *Xenopus*,^11^ the molecular mechanisms underlying the FOXJ1 and RFX cooperation remain poorly characterized in humans. In humans, a dozen distinct *FOXJ1* pathogenic variations have been identified in the heterozygous state in patients with primary ciliary dyskinesia (PCD, OMIM 244400), a rare genetic disease caused by motile cilia dysfunction, leading to impaired mucociliary clearance and chronic airway infections.^12^ PCD is a clinically and genetically heterogeneous disorder with ∼60 genes currently implicated. Nearly one half of affected individuals present with organ laterality defects due to abnormal nodal cilia beating. In addition, the structural similarities between cilia and sperm flagella explain the infertility observed in the majority of affected men. All reported patients with *FOXJ1*-related PCD also present with hydrocephalus, an uncommon feature of the disease.^13-19^ In contrast to the typical autosomal recessive inheritance of PCD, FOXJ1-related PCD follows an autosomal dominant pattern. The ciliary phenotype is characterized by a reduced generation of multiple motile cilia reflecting impaired motile ciliogenesis. Such a defect is also observed in human airway epithelial cells (hAECs) from patients with pathogenic variations in *CCNO* or *MCIDAS*, who share the combined phenotype of PCD and hydrocephalus, but with autosomal-recessive inheritance.

In 2013, Didon  et al. showed a direct interaction between RFX3 and FOXJ1 in human airway MCCs, but the lack of knockdown and chromatin-immunoprecipitation sequencing (ChIP-seq) analyses precluded the identification of their transcriptional targets.^1^ To date, the direct FOXJ1 target genes in hAECs has not yet been defined, leaving a fundamental gap in our understanding of human motile ciliogenesis and the molecular basis of FOXJ1-related PCD.

In this study, we report 2 novel *FOXJ1* pathogenic variants—c.867dup, p.(Ser290Glnfs*12) and c.924del, p.(Phe309Leufs*24)—identified in 3 unrelated patients with PCD and hydrocephalus. To explore the molecular consequences of *FOXJ1* deficiency, we performed comparative RNA-seq on AECs from affected individuals, revealing both potential direct and indirect transcriptional targets. We next carried out ChIP-seq in hAECs to define the genome-wide binding profile of FOXJ1. Integrating these datasets uncovered a central role for FOXJ1 in orchestrating the expression of core structural components of the human ciliary axoneme, providing the first direct map of its transcriptional program in human motile ciliogenesis.

## Material and methods

### Patients

Written informed consent was obtained from all affected individuals and/or their parents following protocols approved by the Comité de Protection des Personnes (CPP) Ile-de-France III and the Institutional Review Board of the French Institute of medical research health (CPP02748 and CEEI-IRB: opinion number 15 and 59, respectively).

### PCD-related investigations

Nasal nitric oxide measurements, ciliary beat frequency of motile cilia on airway brushings as assessed by high-speed videomicroscopy (HSVM), and ultrastructural analyses of cilia by transmission electron microscopy (TEM) were performed as previously described.^12^^,^^20-22^

### Genetic analyses

The identification of pathogenic variations in *FOXJ1* (RefSeq NM_001454) was performed by next-generation sequencing and confirmed by Sanger sequencing as described in Supplemental methods.

### Air-liquid interface (ALI) cell cultures

Airway epithelial cells were obtained from nasal brushings of patients DCP521 and 17GM0068, as well as from surgical material from non–PCD-affected individuals with nasal polyps requiring surgery. The cells were then cultured at ALI as previously described.^23^

### Immunofluorescence studies

Human AECs obtained from fresh nasal brushing (ex vivo hAECs)or ALI-cultured cells (in vitro hAECs) were cytocentrifuged onto glass slides, air-dried, and directly used for immunostaining as previously described.^22^

### RNA extraction and RNA-sequencing (RNA-seq)

RNA-seq were carried out on RNA extracted from hAECs cultured at ALI for 1 month (17GM00680 and three controls) or 2 months (DCP521) and described in Supplemental Methods.

### Chromatin-immunoprecipitation sequencing

As fully described in Supp Method, ChIP-seq was performed by Active Motif (Carlsbad, CA) following 5 steps: chromatin preparation, ChIP reactions, library generation, sequencing, and basic data analysis.

### Bioinformatic analyses

RNA-seq and ChIP-seq bioinformatic analyses are described in Supplemental Methods.

### Identification of direct FOXJ1 targets by integrating ChIP-seq and RNA-seq data

Genes both differentially expressed in FOXJ1-deficient patients (measured by RNA-seq), and with a FOXJ1 peak within their gene body or up to 5 kb upstream of their Transcription Start Site were annotated as direct FOXJ1 targets. Other genes differentially expressed in FOXJ1-deficient patients but without a FOXJ1 peak were annotated as indirectly regulated genes. In parallel, differentially regulated genes with a multiciliated/secretory cell expression ratio > 4 in single-cell RNA-seq analyses^24^ were annotated as multiciliated cell-enriched genes, and those with a multiciliated/secretory cell expression ratio < 0.25 were annotated as secretory cell-enriched genes. Overrepresentation of biological functions within multiciliated cell-enriched down-regulated genes was performed using Metascape.^25^ A schematic overview of the study is in Figure S1.

### Data availability

RNA-seq and ChIP-seq data have been deposited in the European Nucleotide Archive (ENA) at EMBL-EBI under accession number PRJEB99018.

## Results

### Identification of 2 novel heterozygous *FOXJ1* pathogenic variants in 3 independent patients with PCD and hydrocephalus

A targeted DNA-sequencing approach (see Methods), targeting the main genes involved in PCD, identified the same novel heterozygous pathogenic variant—c.867dup, p.(Ser290Glnfs*12)—in 2 unrelated patients with PCD and hydrocephalus, 1 male (DCP521) and 1 female (17GM00680). This variant is located in the last exon of *FOXJ1* [MIM: 618699, 602291] (Table 1), a gene located on chromosome 17, consisting of 3 coding exons (RefSeq NM_001454), and encoding a 421 amino acid protein (Figure 1A). A third unrelated female patient (8821GM000400) with a PCD phenotype associated with hydrocephalus carried another novel *FOXJ1* heterozygous pathogenic variant in *FOXJ1*, c.924del p.(Phe309Leufs*24), also in the last exon, downstream of the Forkhead protein domain (Figure 1A). The 2 patients with the same *FOXJ1* pathogenic variant had laterality defects: *situs inversus totalis* (DCP521) and transposition of the great vessels (17GM00680). One of the 2 female patients (8821GM000400) presented hypofertility with tubal obstruction. Fertility status could not be assessed in the male patient (DCP521), who was too young for evaluation, nor in patient 17GM00680. The intrafamilial segregation analysis revealed that the *FOXJ1* variants occurred de novo in 2 patients (DCP521 and 8821GM000400) (Figure S2). The inheritance pattern could not be determined in patient 17GM00680, as her father was deceased. As detailed in Table 2, nasal nitric oxide levels were within the normal range for both DCP521 and 17GM00680. Interestingly, all 11 *FOXJ1* pathogenic variations previously reported in the literature are also located in the last exon and are heterozygous associated with a similar clinical phenotype (ie, hydrocephalus, upper and lower respiratory symptoms, and laterality defects in about one half of the cases) (Figure 1A, 1B).

**Figure 1:**
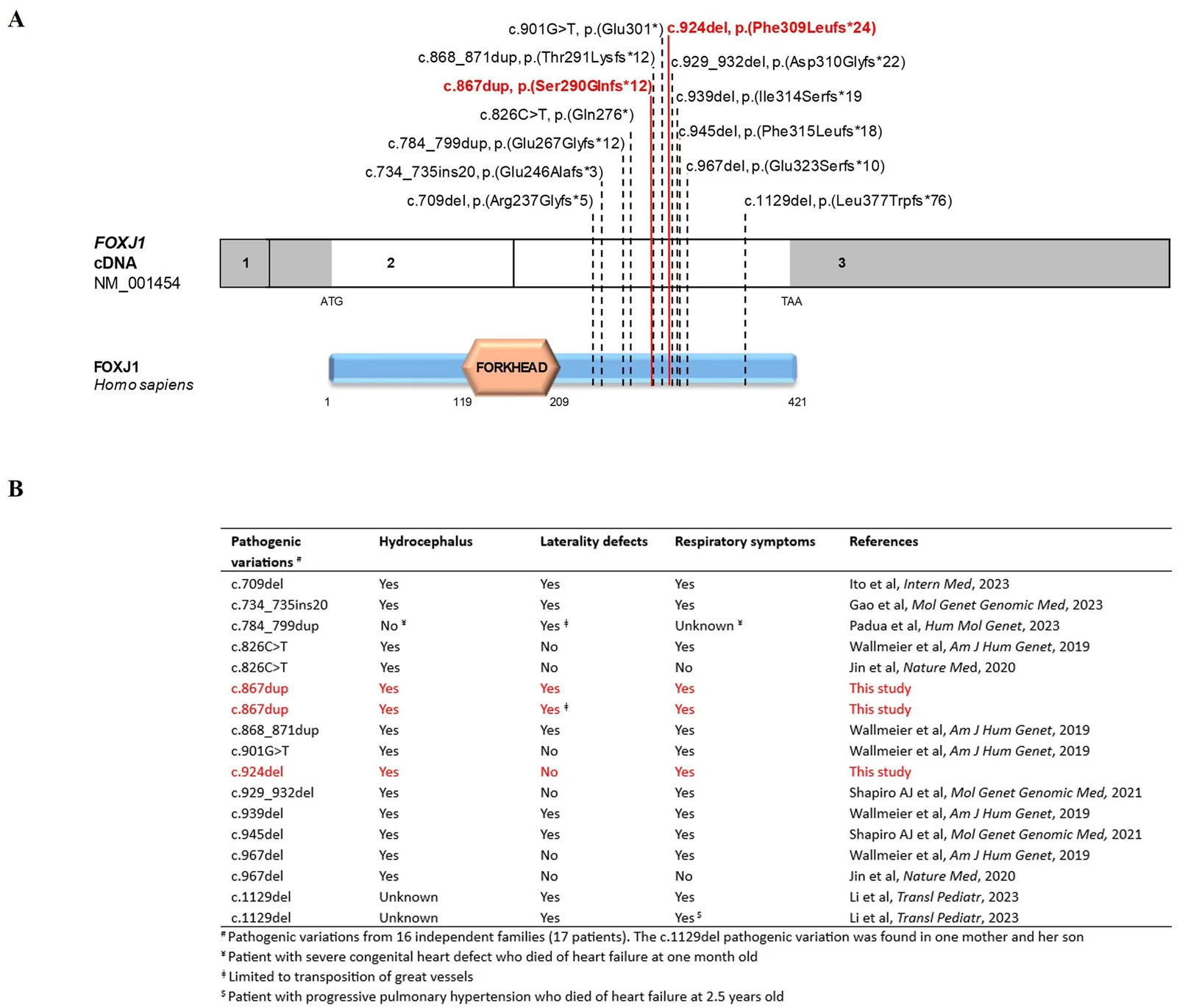
Location of newly identified and previously reported heterozygous *FOXJ1* pathogenic variations along with their associated clinical phenotype. (A) The newly identified *FOXJ1* variations c.867dup (patients DCP521 and 17GM00680) and c.924del (patient 8821GM000400) are shown in red within the exonic organization of the human *FOXJ1* cDNA whereas the 11 previously described pathogenic variations are shown in black. Domain-organization model of the corresponding WT FOXJ1 protein according to the SMART webtool and the NCBI Conserved Domain Database. The 13 *FOXJ1* variations are predicted to lead to a premature stop codon in the last exon of *FOXJ1*. They are all located in a mutation hotspot of 420bp in exon 3 that represents 33% of the *FOXJ1* coding sequence. (B) Clinical phenotypes associated with the heterozygous *FOXJ1* pathogenic variations reported in humans.

**Table 1:** Clinical features of PCD patients with a heterozygous *FOXJ1* pathogenic variation.

Patients (origin)	Known consanguinity	Sex (age at PCD diagnosis)	Laterality defect	Airway disease		Fertility	Hydrocephalus (age at diagnosis)	Genotype	Expression pattern
				Lower	Upper				
**DCP521** **(France)**	No	Male (6 years)	SI totalis	NNRD Chronic bronchitis Bronchiectasis	Rhinosinusitis	NA	Yes (11 years)	c.[867dup];[=] p.[(Ser290Glnfs*12)];[=]	Autosomal dominant De novo
**17GM00680** **(France)**	No	Female (31 years)	Transposition of the great vessels^a^	NNRD Chronic bronchitis Bronchiectasis Pneumopathies	Rhinosinusitis Nasal polyposis	NA	Yes (3 months)	c.[867dup];[=] p.[(Ser290Glnfs*12)];[=]	Autosomal dominant^b^
**8821GM000400** **(France)**	No	Female (27 years)	No	Chronic bronchitis Bronchiectasis Pneumopathies	Rhinosinusitis Nasal polyposis	Hypofertility (tubal obstruction)	Yes (childhood)	c.[924del];[=] p.[(Phe309Leufs*24)];[=]	Autosomal dominant De novo

Abbreviations: NA, not available; NNRD, neonatal respiratory distress; SI, situs inversus.

a Inferior vena cava and aorta.

b Impossible segregation (father deceased).

**Table 2:** Ciliary investigations in PCD patients with a heterozygous *FOXJ1* pathogenic variation.

Patients	Nasal NO^a^	**CBF (Hz)** ^b^	HSVM	TEM results (abnormal cilia %; main defect)
**DCP521**	165nL/min	5 ±0.7	Rare and short cilia Rare immotile cilia	Very few cilia Rare basal bodies (5%; variable defects)
**17GM00680**	117nL/min	6.3 ± 1.4	Rare and short cilia Some immotile cilia	Very few cilia Rare and deep cytoplasmic basal bodies (27%; CC)
**8821GM000400**	NP	NP	NP	Very few cilia Rare and deep cytoplasmic basal bodies (33%; axonemal disorganization)

Abbreviations: CBF, ciliary beat frequency; CC, central complex; HSVM, high speed videomicroscopy; NO, nitric oxide; NP, not performed; TEM, transmission electron microscopy.

a Nasal NO threshold for PCD diagnosis < 77 nL/min.^26^

b CBF mean in controls: 8.83Hz ± 0.26 Hz.^27^

### Impact of heterozygous *FOXJ1* pathogenic variants on the ciliary phenotype, the FOXJ1 protein, and its posttranslational modifications

The impact of identified heterozygous *FOXJ1* variants on the ciliary phenotype was studied by TEM, HSVM, and high-resolution immunofluorescence (IF) analyses. TEM analysis of airway cilia of the 3 patients revealed: (1) a drastically reduced number of cilia (Table 2, Figure S3), with those present displaying an essentially normal axonemal ultrastructure on transverse sections, including both dynein arms, and (2) an abnormal localization of the basal bodies, which were positioned deep in the cytoplasm of all ciliated cells on longitudinal sections. HSVM performed on fresh nasal brushings from patients DCP521 and 17GM00680 confirmed the presence of very rare and short cilia in both patients (Table 2). Almost all cilia were motile with a beating pattern comparable to controls and a beat frequency slightly reduced. Representative movies of the rare area with beating cilia are shown in supplemental data (Movies S1, S2, S3). High-resolution IF analysis on freshly collected hAECs from these 2 patients compared to controls also confirmed the rarefaction of cilia in both patients (Figure S4). Furthermore, the presence of ODAs and IDAs, initially revealed by TEM, was also confirmed in all remaining cilia by DNAH5 and DNALI1 immunolabeling, respectively (Figure S4). To further investigate the impact of heterozygous *FOXJ1* variations on respiratory cilia, we performed additional immunolabeling on hAECs cultured at ALI from DCP521 and a control. We used antibodies directed against (1) DNAH5 and DNAH9 as markers of the ODAs, (2) DNALI1 as marker of the IDAs, (3) ROOTLETIN as marker of the ciliary rootlet, (4) CEP164 as marker of basal bodies, (5) GAS2L2 as marker of basal feet with actin connexion, and (6) ARMC4/ODAD2 as marker of the ODA docking complex (Figure 2A). Noteworthy, the labeling levels of GAS2L2 and ARMC4/ODAD2 were markedly reduced in the patient sample, whereas those of other markers were not substantially altered compared to the control sample (Figure 2A, B). Furthermore, the length of the cilia microtubules, as assessed after labelling with an anti-alpha acetylated tubulin antibody, was significantly shorter in the DCP521 sample than in the control (Figure 2C).

**Figure 2:**
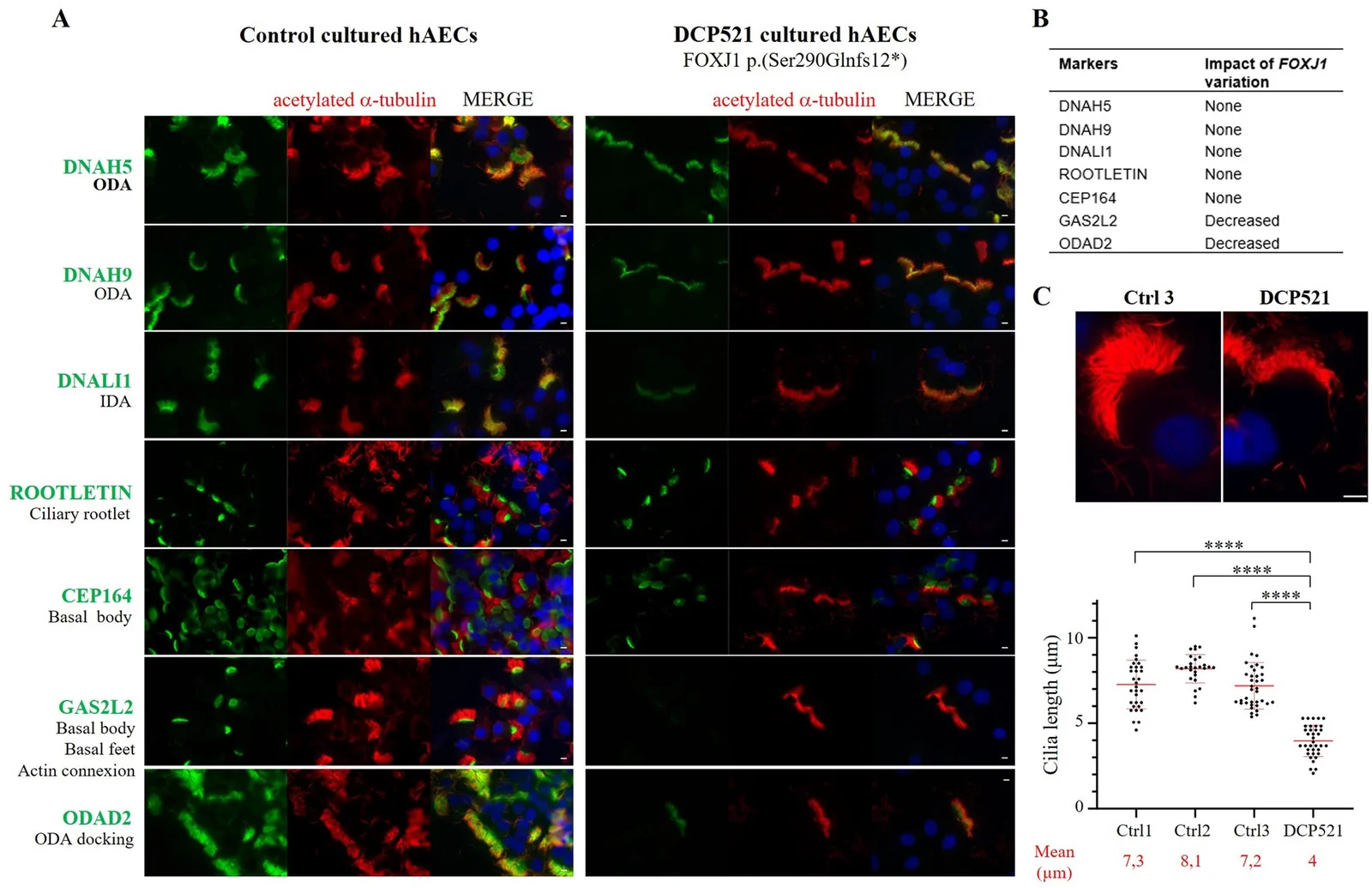
Impact of *FOXJ1* pathogenic variations on axonemal markers and cilia length in hAECs. (A) Immunofluorescence images of ALI-cultured hAECs from a control individual (Ctrl 3) and the DCP521 individual heterozygous for the *FOXJ1* c.867dup variation. Cells were double-labeled with a marker of ciliary axonemes (anti–α-tubulin antibody, red), together with anti-DNAH5 or anti-DNAH9 antibodies as markers of ODAs (green), anti-DNALI1 antibody as marker of IDAs (green), anti-CEP164, or anti-ROOTLETIN as markers of basal body and ciliary rootlet respectively, anti-GAS2L2 as marker of basal body/basal foot/actin connexion, and anti-ARMC4 as marker of ODA docking. Nuclear staining was performed with DAPI (blue). In DCP521, all markers are present, but GAS2L2 and ARMC4 are significantly decreased. (B) Table summarizing immunofluorescence results. (C) Representative image of hAECs immunolabeled with an anti-tubulin antibody (red) showing a significantly reduced cilia length in AECs from patient DCP521 compared to one control (Ctrl 3). Dot plot represents cilia length in 32, 30, 37, and 36 independent cells from 3 Ctrl and 1 patient (DCP521), respectively. Means ± SD for each sample are in red. Statistical analysis was performed using GraphPad Prism v9 software with 2-tailed unpaired *t*-tests, ****P < .0001.

To study the impact, at the protein level, of a frameshift variation leading to a premature stop codon in *FOXJ1*, airway cells from patient DCP521 and controls were grown at ALI, and the total protein extracts were analyzed by immunoblotting with an anti-FOXJ1 antibody. The detection of both wild-type (WT) and truncated FOXJ1 proteins in the patient DCP521 who carries the c.867dup, p.(Ser290Glnfs*12) variation in the heterozygous state indicates that the mutated transcripts escape nonsense-mediated mRNA decay (NMD) (Figure 3A). Interestingly, in the patient’s AECs, 2 molecular species of close molecular mass (∼ 37 KDa) were detected, corresponding to 2 truncated forms of the FOXJ1 protein (Figure 3A). In addition, the analysis of FOXJ1 by IF in patients’ AECs revealed that FOXJ1 is still only located inside the nucleus of MCCs (Figure 3B). These results have been confirmed in HeLa cells transiently overexpressing WT or mutated FOXJ1 proteins (Figure S5A). The predicted truncated proteins p.(Ser290Glnfs*12) and p.(Phe309Leufs*24) are illustrated in Figure S5B. To rule out the existence of another FOXJ1 isoform or any splicing effect of the variant, immunoblotting was repeated with total protein extracts from HeLa cells transfected with plasmids expressing the WT or mutated *FOXJ1* cDNAs (Figure S5C). This confirmed the presence of 2 molecular species for the mutated FOXJ1 (ie, truncated proteins). Taken together, these data are consistent with posttranslational modification(s)—PTM(s)—occurring in both native hAECs and transfected HeLa cells. With the aim to identify such PTM(s), we treated protein lysates from normal hAECs and HeLa cells transfected with either the WT or the mutated *FOXJ1* constructs with lambda phosphatase. This experiment revealed that the observed PTMs correspond to the phosphorylation of 2 distinct FOXJ1 sites: 1 located upstream of the first mutation site (Ser290), and the other located downstream of the second mutation site (Phe309) (Figure S6A). Notably, the phosphorylation pattern remained stable even after 21 days in ALI culture, when cells are fully differentiated (Figure S6B). Indeed, *FOXJ1* transcript expression is maintained in hAECs at least until day 90, in contrast to *CCNO* and *MCIDAS* expression, which shows a drastic decrease by day 21 (Figure S6C).

**Figure 3:**
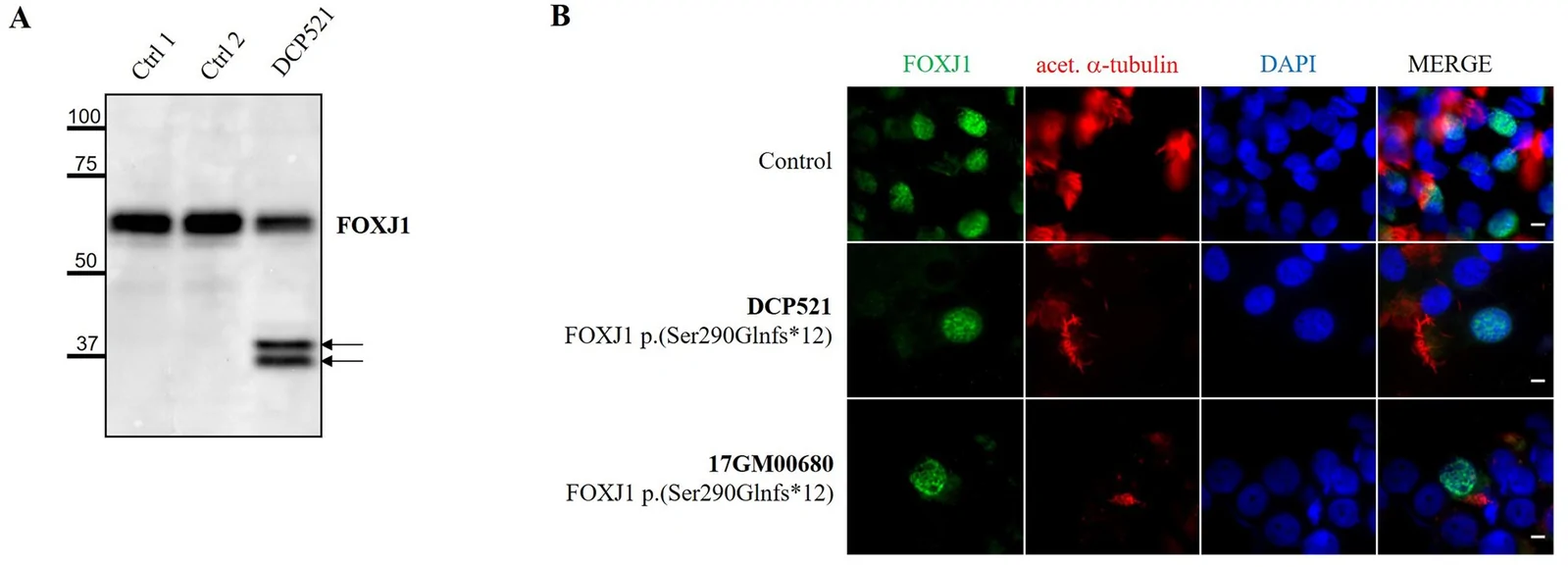
Subcellular localization and characterization of WT versus mutated FOXJ1 protein in ALI-cultured hAECs. (A) Detection of the FOXJ1 protein by immunoblot in hAECs from 2 controls and individual DCP521. In cell lysate from DCP521, both WT and mutated forms of FOXJ1 are detected. The mutant form appears as 2 distinct bands (arrows). (B) Immunofluorescence microscopy analysis of FOXJ1 in hAECs from a control and 2 PCD patients (DCP521 and 17GM00680), both heterozygous for the c.867dup *FOXJ1* variation. Cells were double-labeled with a marker of ciliary axonemes (anti-α-tubulin antibody, red), together with anti-FOXJ1 antibody (green). Nuclear staining was performed with DAPI (blue). Scale bars represent 5 μm.

### Differentially expressed protein-coding genes (DEGs) in PCD patients with a heterozygous *FOXJ1* pathogenic variation

In order to identify DEGs in patients with a heterozygous *FOXJ1* pathogenic variation, RNA-seq analysis was performed on ALI-cultured AECs from DCP521 and 17GM00680, as well as on ALI-cultured AECs from 3 control individuals. This analysis showed that 393 genes are differentially expressed in patients, with 95 and 298 genes up- and down-regulated, respectively (Figure 4A). Before any interpretation, we first assessed whether the *FOXJ1* molecular defects could affect the expression of key regulators and transcription factors involved in motile ciliogenesis, including *GMN1*, *MCIDAS*, *CCNO*, *TP73,* and *RFX2*. Our analysis showed no such effect (Table S3), consistent with the fact that all these genes act upstream of *FOXJ1* in the signalling cascade activated during motile ciliogenesis.^28^ In addition, consistent with data from western blots assays, this RNA-seq analysis confirmed that the mutated *FOXJ1* transcripts escaped NMD in patients since (1) *FOXJ1* transcript levels in patients were comparable to those in controls (Table S3), and (2) the *FOXJ1* coding heterozygous SNPs showed balanced variant allele fraction in both patients (Table S5).

**Figure 4:**
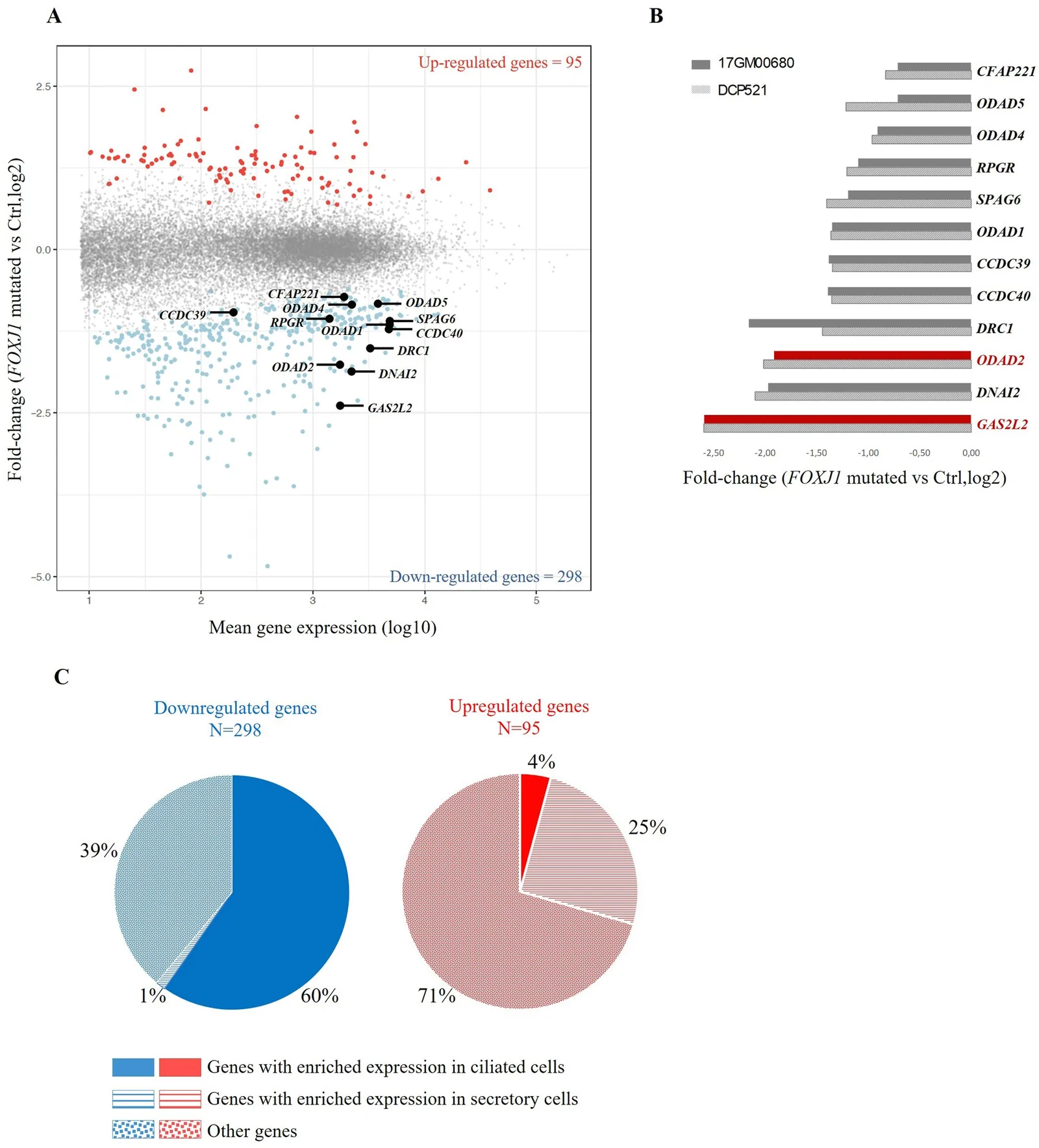
DEGs in hAECs from 3 control individuals and 2 PCD patients (DCP521 and 17GM00680) heterozygous for a *FOXJ1* pathogenic variation. (A) The MA plot represents gene expression (X-axis, log10 mean gene expression) and fold-change (Y-axis, log2 fold-change). A minimum fold-change of ± 1.5 and a maximum adjusted *P* value of .1 are used as thresholds to identify DEGs (95 up-regulated genes [red], 298 down-regulated genes [blue]). Genes implicated in PCD are annotated in black. (B) Histogram showing for each patient, the log2 fold-change of the 12 PCD genes that are downregulated. The values are highly similar in both patients. In red are the 2 genes whose decreased expression could have been assessed by immunofluorescence on AECs from DCP521 in Figure 3. (C) Distribution of DEGs into 3 categories: genes whose expression is enriched in multiciliated cells, in secretory cells, and in other cell types. The left pie chart shows down-regulated genes (*N* = 298), whereas the right pie chart shows up-regulated genes (*N* = 95). Multiciliated cell–enriched genes correspond to genes with a multiciliated/secretory cell expression ratio > 4, whereas secretory cells-enriched genes correspond to a secretory/multiciliated cell expression ratio < 0.25.^57^ Sixty percent of the 298 down-regulated genes are expressed in multiciliated cells.

Among the 298 downregulated genes in patients with a pathogenic *FOXJ1* variation, 12 are known to be implicated in PCD. With the exception of *DRC1*, these genes showed a comparable reduction in expression in both patients, ranging from a 1.75-fold decrease for *CFAP221* (log2FC = 0.78) to a 6-fold decrease for *GAS2L2* (log2FC = 2.5) (Figure 4B). In particular, 178 (60%) of the 298 down-regulated genes have a physiological expression that is enriched in MCCs compared to secretory cells (Table S3 and Figure 4C). In contrast, among the 95 genes that are up-regulated in the patients, only 4 (4%) are physiologically enriched in MCCs, whereas 24 (25%) are enriched in secretory cells (Figure 4C).

### FOXJ1 ChIP-seq in hAECs and de novo identification of target motifs

To determine which of the DEGs are directly regulated by FOXJ1, we performed a chromatin immunoprecipitation assay using an anti-FOXJ1 antibody in control hAECs in ALI culture, followed by high-throughput sequencing (ChIP-seq). The bioinformatics analyses identified 785 FOXJ1 peaks. We observed significant enrichment (adjusted *P* ≤ .01, fold-change ≥ 2) of FOXJ1 binding sites upstream promoters and proximal enhancers, especially when they are associated with CTCF binding sites, and a relative depletion of FOXJ1 binding sites at distal enhancers, H3K4me3 regulatory sites, or CTCF binding sites not associated to promoters. Overall, the identified 785 FOXJ1 peaks correspond to 683 protein-coding genes, with a mean size ± SD of 237 ± 63 pb (Table S4).

Two target motifs were de novo identified: GTAAACAA corresponding to a known binding motif of the FOX protein family members, and GTTGCYAKGGAR corresponding to a known binding motif of RFX protein family members (Figure 5A). The RFX motifs were observed more frequently (71%) than the FOXJ1 motifs (27%). A third de novo motif (CHTRGWKAC) was identified, also corresponding to the RFX motif family. Notably, both RFX motifs identified constitute a partially degenerate palindrome which explains why the 2 different motifs match the same putative full-length palindromic motif (Figure 5B). RFX and FOXJ1 motifs were both mainly observed at the center of the FOXJ1 peaks (Figure 5C). When the 2 motifs were located in the same peak (251 occurrences), we found that the RFX motif was reproducibly positioned immediately upstream (from –1 to –9 bp) from the FOX motif (median = –2, binomial test *P* = 1.29.10^−12^), indicating that both factors bind DNA in close proximity to each other (Figure 5D).

**Figure 5:**
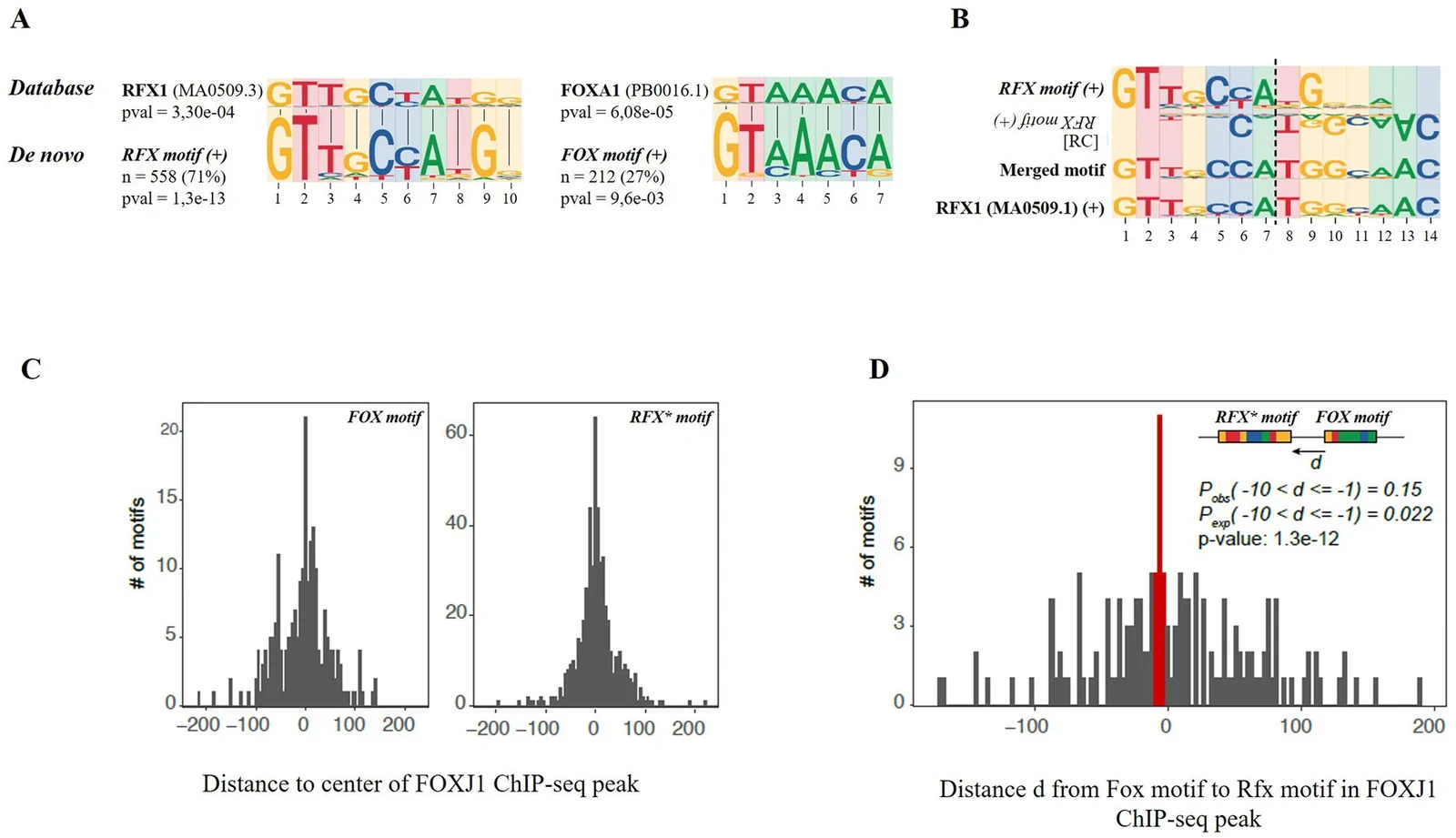
Analysis of FOXJ1 and RFX motifs in endogenous FOXJ1 ChIP-seq peaks in control hAECs. (A) De novo identification of motifs enriched in the endogenous FOXJ1 ChIP-seq peaks using XSTREME.^56^ The 2 most significant motifs are shown. TOMTOM was used to compared them to annotated motifs from JASPAR database^39^ and found significant hit with DNA-binding motifs from the RFX and FOX families (shown above the de novo motifs). Note that the RFX motif is more prevalent (∼ 71%) than the FOX motif (∼ 27%) in the FOXJ1 peaks set. (B) Full-length de novo RFX motif aligned with its own reverse complement (RC) reveals that the RFX motif is a palindrome. (C) Position of FOX and RFX motifs in FOXJ1 ChIP-seq peaks. The position is indicated as the distance to the center of FOXJ1 ChIP-seq peaks. (D) Distance from the FOX motif to the RFX motif, in all FOXJ1 peaks where both motifs were found. FOX motifs are significantly enriched 1–9 bp downstream of a RFX motif. In red: RFX motifs located immediately upstream (–1 to –9 bp) from FOX motif. P_obs_ = 23/156 = 0.15 is the observed fraction of FOX motifs with an RFX motif immediately upstream (–1 to –9 bp); P_exp_ = 9/400 = 0.022 is expected fraction of FOX motifs with an RFX motif immediately upstream (–1 to –9 bp) under the assumption of a uniform distribution of RFX motifs.

### Direct vs indirect FOXJ1 targets and functional annotation of the 178 MCC-specific down-regulated genes identified in patients with *FOXJ1*-related PCD

To generate a list of DEGs that are directly or indirectly regulated by FOXJ1, we integrated the RNA-seq data, which identified DEGs in AECs from patients with a heterozygous pathogenic *FOXJ1* variation, and the FOXJ1 ChIP-seq data obtained in control hAECs. This analysis revealed a highly significant overrepresentation of down-regulated genes among FOXJ1 targets (17.6-fold enrichment, Fisher exact test *P* = 1.16.10^−76^), and that 105 of the 393 DEGs in FOXJ1 patients are direct targets of FOXJ1 (Table S3, Figure 6A). More precisely, in patients with a pathogenic *FOXJ1* variation, among the 178 down-regulated genes that have a physiological expression enriched in MCCs, half (*n* = 89) are directly regulated by FOXJ1 (Figure 6B). In contrast, none of the 4 up-regulated genes that are physiologically enriched in MCCs is a direct target of FOXJ1; only 1 gene, *MBOAT7*, appears as a direct target of FOXJ1, but its expression is not enriched in either multiciliated or secretory cells.

**Figure 6:**
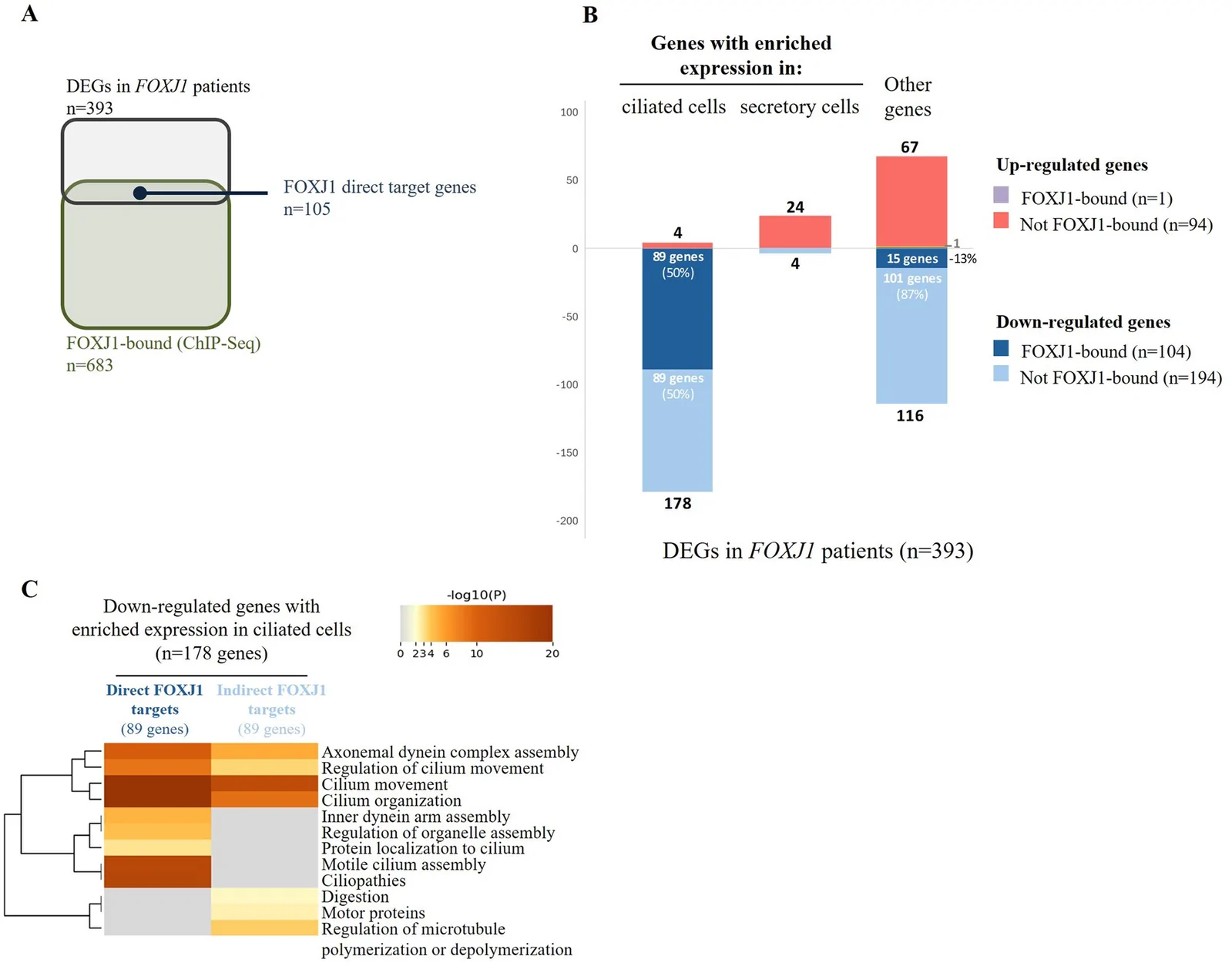
Identification of FOXJ1 direct targets through the integrated analysis of FOXJ1 ChIP-seq and RNA-seq data. (A) Schematic representation of the integrated data from FOXJ1 ChIP-seq and RNA-seq showing 105 genes directly targeted by FOXJ1 and differentially expressed in hAECs from the 2 PCD patients (DCP521 and 17GM00680) heterozygous for a *FOXJ1* pathogenic variation. (B) DEGs, grouped into multiciliated cell–enriched genes, secretory cell–enriched genes, and other DEGs. Multiciliated cell–enriched genes correspond to genes with a multiciliated/secretory cell expression ratio > 4, whereas secretory cell–enriched genes correspond to a secretory/multiciliated cell expression ratio < 0.25.^57^ In each group, DEGs are classified as either direct FOXJ1 targets (directly bound by FOXJ1), or indirect targets regulated by FOXJ1 (not directly bound by FOXJ1). (C) Heatmap generated with the Metascape webtool^22^ of enriched terms (colored by *P* values) across the list of the 178 down-regulated genes identified in patients and whose expression is enriched in MCC, split into direct or indirect FOXJ1 targets.

To functionally annotate the 178 genes that are downregulated in patients and have a MCC-specific expression profile, we drew a heat map of enriched terms across this list of genes using Metascape.^25^ Overall, this analysis revealed the importance of these genes in cilia organization, assembly, and movement, with functional importance being even more pronounced for genes that are direct targets of FOXJ1 (Figure 6C). In addition, in an attempt to identify specific features shared by proteins encoded by the genes downregulated in *FOXJ1* patients, we confronted the RNA-seq data from these patients and the function/localization of proteins that are crucial for cilia motility. To this end, we first reviewed the cryotomographic data of previous studies focusing on structural axonemal proteins,^29-36^ and then considered proteins known to be involved in the assembly of axoneme-decorating structures in motile cilia. This analysis enabled us to determine the location of 151 proteins that could be divided into 2 groups: those in contact with axonemal tubulin (*n* = 72) and those with no contact with tubulin (*n* = 79) (Figure S7A). Notably, 29 of the 72 genes encoding proteins directly bound to axonemal tubulin were found to be downregulated in AECs from PCD patients heterozygous for a pathogenic *FOXJ1* variation, whereas only 6 of 79 genes encoding proteins not bound to tubulin were found to be downregulated (χ^2^ = 21.15, *P* = 4.23.10^−6^). This strongly suggests that FOXJ1 may predominantly regulate the expression of proteins that are in close contact with tubulin and that pioneer the docking of protein complexes such as dynein arms and radial spokes. Interestingly, 21/29 of the downregulated genes coding for proteins in contact with axonemal tubulin were captured in FOXJ1 ChIP-seq, thus corresponding to genes directly regulated by FOXJ1. Furthermore, examination of the functional/structural groups of these 151 ciliary proteins revealed that numerous proteins whose expression is highly downregulated by FOXJ1 belong to the microtubule-inner proteins or to the docking complex (Figure S7B, S7C). Conversely, none of the dynein heavy chains and none of the components of the DNAAFs seem to be regulated by FOXJ1.

## Discussion

In this study dedicated to the transcription factor FOXJ1, a master regulator of motile ciliogenesis, we provide the first description in humans of its transcriptional targets (both direct and indirect), by combining 2 genome-wide approaches (ie, ChIP-seq in normal hAECs and RNAseq in hAECs from patients with *FOXJ1*-related PCD associated with hydrocephalus). Our data also enabled refinement of the consensus target motifs for both FOXJ1 and the RFX family transcription factors, which are also key regulators of motile ciliogenesis. These motifs were found in close proximity, and—together with previous reports in other species^7^^,^  ^11^—strongly support a FOXJ1/RFX functional cooperation in the human motile ciliogenic program.

The ciliary phenotype observed in our patients—used to define the FOXJ1-dependent gene network in hAECs—is characteristic of impaired motile ciliogenesis. TEM analyses showed fewer cilia, basal body mislocalization, and markedly shorter cilia, with lengths nearly one half those of the control subjects. These short cilia resemble those in tracheal MCCs from *Foxj1-*deficient mice^14^ but are rare in PCD caused by other genes, except in cases with pathogenic variations in the kinase-encoding gene *NEK10*^37^ or the *TUBB4B* gene that encodes a cilia-specific tubulin.^38^ Interestingly, in patients with biallelic pathogenic variants of the *NEK10* gene, shorter cilia appear to result in altered mucociliary transport and a thinner periciliary layer.^37^ However, in patients with a *FOXJ1* pathogenic variant, the drastic reduction in the number of cilia alone can underlie the mucociliary clearance defect. As for patients with pathogenic *TUBB4B* variations, 75% of them also have hydrocephalus, probably from a defect in the MCCs of the choroid plexus as shown in *Foxj1* knockout mice.^39^

Interestingly, almost all patients with pathogenic *FOXJ1* variants reported in the literature, as well as those described in this study, exhibit normal nasal nitric oxide values. This may be explained by the presence of residual ciliary beating, as previously reported.^21^ Consistently, patients with genotypes associated with milder forms of PCD—such as *RSPH1* or *CCDC103* p. His154Pro variants—which are also characterized by residual ciliary activity on HSVM, display higher nasal nitric oxide levels. These observations suggest that, although the remaining cilia in patients carrying pathogenic *FOXJ1* variants are shorter than in controls, they may still generate sufficient shear stress to account for the preserved nasal nitric oxide levels.

Beyond the lung disease, 1 of the affected women in the present study exhibited hypofertility, in line with previous reports of hypofertility in 3 other women.^13^^,^^14^^,^^16^ Moreover, 1 man heterozygous for a pathogenic *FOXJ1* variation was also reported as infertile.^13^ These findings support a role for FOXJ1 in the motile ciliogenesis of MCCs in the oviduct and the efferent ducts and/or in spermatogenesis.

Interestingly, all 13 of the pathogenic *FOXJ1* variants identified to date are all located within a mutational hotspot downstream of the Forkhead DNA-binding domain. In this study, we show that *FOXJ1* transcripts with the c.867dup variant, one of the earliest reported premature stop codons, escape NMD and result in protein production.

This finding suggests that *FOXJ1* variants truncated downstream of the Forkhead DNA-binding domain may retain DNA-binding capacity while impairing transcriptional activity through a dominant-negative effect. In line with this, Li  et al.^14^ reported a dominant-negative mechanism for the c.1129del p.(Leu377Trpfs*76) variant, both in vitro and in vivo.

Indeed, whereas mice with a homozygous targeted deletion of the entire *Foxj1/Hfh4* gene display defective airway epithelial ciliogenesis and randomized left–right asymmetry only in the homozygous state,^40^^,^^41^ those heterozygous for the c.1129del variant already exhibit situs inversus, hydrocephalus, and a reduced number of tracheal cilia.

In contrast to MCIDAS and CCNO, the expression of FOXJ1 rises at the late stage of ciliogenesis in hAECs and remains highly expressed beyond the end of motile ciliogenesis (for at least until 3 months). This suggests that, in humans, FOXJ1 may contribute to maintain differentiation in MCCs and prevent their dedifferentiation. Such a role has been delineated in mouse multiciliated ependymal cells, where the persistence of ciliary differentiation depends on the IKK2 kinase, which stabilizes FOXJ1 through its phosphorylation.^42^ In our study, we showed that, human FOXJ1 is phosphorylated on at least 1 N-terminal and 1 C-terminal site. Given the evolutionary conservation of the IKK2 target site encompassing serines 32/35 within the N-terminal region of FOXJ1 (ie, DALDDSLTS),^42^ we hypothesize that the N-terminal phosphorylation may also stabilize human FOXJ1, as observed in mice. Further investigations are needed to substantiate this hypothesis in human cells.

Transcriptome analysis of AECs from patients with a heterozygous *FOXJ1* pathogenic variant revealed predominantly downregulated DEGs as a result of both RNA-seq and IF experiments performed in AECs from patients. Among the 298 downregulated genes, 60% (*n* = 178) correspond to genes with an enriched expression in normal MCCs, including 12 PCD genes. In contrast, genes that are physiologically enriched in secretory cells are almost exclusively upregulated in FOXJ1 patients (24/28). This pattern could reflect either a reduced proportion of MCCs or a partial fate shift of progenitors towards the secretory lineage. Single-cell RNA-seq analyses would provide valuable insight into cell-type proportions.

To better characterize the transcriptional network controlled by FOXJ1, we performed the first FOXJ1 ChIP-seq experiment in human airway epithelial cells, identifying 683 directly bound protein-coding genes. This dataset extends previous knowledge, which relied mainly on DEGs analyses following Foxj1 overexpression in *Xenopus* or zebrafish, or in *Foxj1*-/- mice, where only 16 genes had been reported as regulated by FoxJ1.^5^ Our ChIP-seq data showed that 10 of these 16 genes are directly targeted in hAECs including *DNAH9*, *DNAI1*, *DNALI1*, *DYNLRB2*, *EFHC1*, *KIF9, RSPH4A, SPAG6*, *SPATA17*, and *TEKT4*. In addition, only 3 direct targets of FOXJ1 have been identified so far by ChIP-PCR: *DNAH9* and *WDR78/DNAI4* in zebrafish,^43^ and *ADGB* (androglobin) recently implicated in motile ciliogenesis through a FOXJ1-dependent regulation in human cell lines transiently expressing FOXJ1-FLAG.^44^ Beyond these, our data uncovered numerous novel FOXJ1 targets, including 14 cilia and flagella associated proteins, 9 axonemal dynein chains, and the transcription factors *RFX1*, *RFX2*, *RFX3*, *TP73*, as well as *FOXJ1* itself.

The de novo determination of FOXJ1 and RFX target motifs in humans has allowed us to precisely refine the consensus motifs previously described in other species^45^^,^^46^ listed in JASPAR database.^47^ The RFX motif was more prevalent than the FOXJ1 motif among FOXJ1 peaks, as previously observed in *Xenopus* overexpressing FOXJ1-FLAG^11^ and in transfected human cell lines.^44^ In our study, we further found that, in humans, FOXJ1 binding loci are located close to gene promoters, with a supporting RFX motif in most cases (72%) situated a short distance upstream from the FOXJ1-binding motif (median distance –2 bp). This supports the hypothesis that RFX factors scaffold FOXJ1 binding to its targets, as suggested in *Xenopus*.^11^ However, our study also revealed a relative depletion of FOXJ1 at distal enhancers, H3K4me3 regulatory sites, and nonpromoter-associated CTCF binding sites—findings that do not support the previously proposed model in *Xenopus*, in which RFX2 is thought to stabilize FOXJ1 binding at chromatin loops.^11^

Cross-analysis of patient transcriptomes and FOXJ1 ChIP-seq data showed that FOXJ1 directly regulates genes—defined as those both dysregulated in patients carrying a pathogenic FOXJ1 variant and bound by FOXJ1 ChIP—that are predominantly enriched in MCCs (89/104, 86%). In contrast, none of the secretory cell-enriched DEGs were directly regulated by FOXJ1, underscoring the specificity of our FOXJ1 binding. Noteworthy, the genes targeted by FOXJ1 in ChIP-seq experiments are not always deregulated in patients with a *FOXJ1* molecular defect. For instance, our results revealed that among the 60 genes so far implicated in PCD, 12 were downregulated in patients with a *FOXJ1* heterozygous variation, of which only 7 corresponded to a direct FOXJ1 target.

Comparison of transcriptomic data from patients carrying *FOXJ1* defects with datasets on the localization and function of MCC-specific proteins, revealed that FOXJ1 directly and indirectly regulates numerous genes encoding proteins in contact with axonemal microtubules, particularly microtubule-inner proteins and docking components. Together with the persistent expression of FOXJ1 in well-differentiated MCCs, this strongly suggests a role of FOXJ1 in maintaining functional cilia by sustaining ciliary proteins, particularly those in direct contact with axonemal tubulin. In addition, the fact that genes encoding heavy chains of dyneins and DNAAFs are not downregulated in *FOXJ1* patients resonates with the recent characterization of cytoplasmic foci that concentrate dynein heavy chain mRNA, chaperones, and DNAAFs.^48-50^ These foci are thought to be hubs dedicated to heavy chains production and assembly to form dynein arms, processes that may therefore be FOXJ1-independent.

In summary, this study provides a functional and molecular characterization of FOXJ1 in human airway epithelial cells. By integrating ChIP-seq data in normal hAECs with RNA-seq analyses of patient-derived cells, we identified a broad network of direct and indirect FOXJ1 transcriptional targets encompassing hundreds of cilia-associated genes. These findings establish FOXJ1 as a key regulator not only of motile ciliogenesis but also of cilia maintenance. They also lay the groundwork for future studies into its role in motile ciliogenesis and *FOXJ1*-related PCD.

## Supplementary Material

aanag071_Supplementary_Data

## Data Availability

**Web resources:** GnomAD, https://gnomad.broadinstitute.org/, OMIM, http://www.omim.org/, SMART, http://smart.embl-heidelberg.de/, Uniprot, https://www.uniprot.org/, Metascape, https://metascape.org, JASPAR2024 https://jaspar.elixir.no/. **Software:** R v4.4.1 R-project https://www.r-project.org/Bioconductor v3.19 [10.1186/gb-2004-5-10-r80], ggplot2 3.5.1 [10.21105/joss.01686], tidyverse 2.0.0 [10.21105/joss.01686], tidyomics 1.0.0 [10.1038/s41592-024-02299-2], meme 5.4.1 [10.1093/nar/gkl198].
