# Supplementary material for: FOXJ1 transcriptional targets in human airway cells and impaired multiciliogenesis in FOXJ1-associated primary ciliary dyskinesia
Source: Am J Respir Cell Mol Biol. 2026 Mar 30;74(11):1553–65. doi: 10.1093/ajrcmb/aanag071 (PMC13720309; doi:10.1093/ajrcmb/aanag071)
Supplement: Supplementary file 1 [file aanag071_supplementary_data.zip › FIG S7 total.pdf]

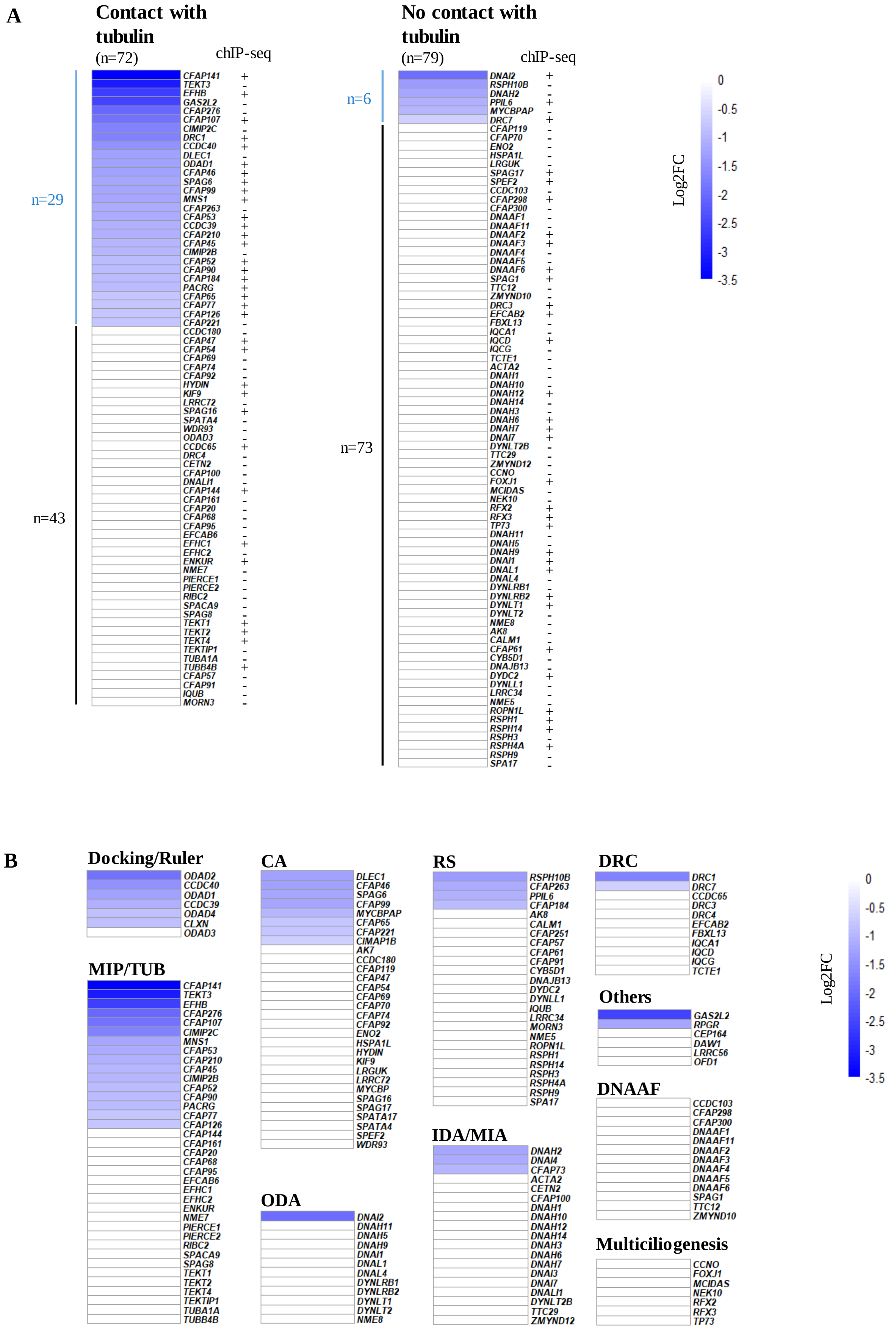
A
Contact with tubulin
(n=72)
chIP-seq
n=29
n=43
CFAP141 +
TEKT3 -
EFHB +
GAS2L2 -
CFAP276 -
CFAP107 +
CIMIP2C -
DRC1 +
CCDC40 +
DLEC1 -
ODAD1 +
CFAP46 +
SPAG6 +
CFAP99 +
MNS1 +
CFAP263 -
CFAP53 +
CCDC39 +
CFAP210 +
CFAP45 +
CIMIP2B -
CFAP52 +
CFAP90 +
CFAP184 +
PACRG +
CFAP65 +
CFAP77 +
CFAP126 +
CFAP221 -
CCDC180 -
CFAP47 +
CFAP54 +
CFAP69 -
CFAP74 -
CFAP92 -
HYDIN +
KIF9 +
LRRC72 -
SPAG16 +
SPATA4 -
WDR93 -
ODAD3 -
CCDC65 +
DRC4 -
CETN2 -
CFAP100 -
DNALI1 -
CFAP144 +
CFAP161 -
CFAP20 -
CFAP68 -
CFAP95 -
EFCAB6 -
EFHC1 +
EFHC2 -
ENKUR +
NME7 -
PIERCE1 -
PIERCE2 -
RIBC2 -
SPACA9 -
SPAG8 -
TEKT1 +
TEKT2 +
TEKT4 +
TEKTIP1 -
TUBA1A -
TUBB4B +
CFAP57 -
CFAP91 -
IQUB -
MORN3 -
No contact with tubulin
(n=79)
chIP-seq
n=6
n=73
DNAI2 +
RSPH10B -
DNAH2 -
PPIL6 +
MYCBPAP -
DRC7 +
CFAP119 -
CFAP70 -
ENO2 -
HSPA1L -
LRGUK -
SPAG17 +
SPEF2 +
CCDC103 -
CFAP298 +
CFAP300 -
DNAAF1 -
DNAAF11 -
DNAAF2 +
DNAAF3 +
DNAAF4 -
DNAAF5 -
DNAAF6 +
SPAG1 +
TTC12 -
ZMYND10 -
DRC3 +
EFCAB2 +
FBXL13 -
IQCA1 -
IQCD +
IQCG -
TCTE1 -
ACTA2 -
DNAH1 -
DNAH10 -
DNAH12 +
DNAH14 -
DNAH3 -
DNAH6 +
DNAH7 +
DNAI7 +
DYNLT2B -
TTC29 -
ZMYND12 -
CCNO -
FOXJ1 +
MCIDAS -
NEK10 -
RFX2 +
RFX3 +
TP73 +
DNAH11 -
DNAH5 -
DNAH9 +
DNAI1 +
DNAL1 +
DNAL4 -
DYNLRB1 -
DYNLRB2 +
DYNLT1 +
DYNLT2 -
NME8 -
AK8 -
CALM1 -
CFAP61 +
CYB5D1 -
DNAJB13 -
DYDC2 +
DYNLL1 -
LRRC34 -
NME5 -
ROPN1L +
RSPH1 +
RSPH14 +
RSPH3 -
RSPH4A +
RSPH9 -
SPA17 -
Log2FC
0
-0.5
-1
-1.5
-2
-2.5
-3
-3.5
B
Docking/Ruler
ODAD2
CCDC40
ODAD1
CCDC39
ODAD4
CLXN
ODAD3
MIP/TUB
CFAP141
TEKT3
EFHB
CFAP276
CFAP107
CIMIP2C
MNS1
CFAP53
CFAP210
CFAP45
CIMIP2B
CFAP52
CFAP90
PACRG
CFAP77
CFAP126
CFAP144
CFAP161
CFAP20
CFAP68
CFAP95
EFCAB6
EFHC1
EFHC2
ENKUR
NME7
PIERCE1
PIERCE2
RIBC2
SPACA9
SPAG8
TEKT1
TEKT2
TEKT4
TEKTIP1
TUBA1A
TUBB4B
CA
DLEC1
CFAP46
SPAG6
CFAP99
MYCBPAP
CFAP65
CFAP221
CIMAP1B
AK7
CCDC180
CFAP119
CFAP47
CFAP54
CFAP69
CFAP70
CFAP74
CFAP92
ENO2
HSPA1L
HYDIN
KIF9
LRGUK
LRRC72
MYCBP
SPAG16
SPAG17
SPATA17
SPATA4
SPEF2
WDR93
ODA
DNAI2
DNAH11
DNAH5
DNAH9
DNAI1
DNAL1
DNAL4
DYNLRB1
DYNLRB2
DYNLT1
DYNLT2
NME8
RS
RSPH10B
CFAP263
PPIL6
CFAP184
AK8
CALM1
CFAP251
CFAP57
CFAP61
CFAP91
CYB5D1
DNAJB13
DYDC2
DYNLL1
IQUB
LRRC34
MORN3
NME5
ROPN1L
RSPH1
RSPH14
RSPH3
RSPH4A
RSPH9
SPA17
IDA/MIA
DNAH2
DNAI4
CFAP73
ACTA2
CETN2
CFAP100
DNAH1
DNAH10
DNAH12
DNAH14
DNAH3
DNAH6
DNAH7
DNAI3
DNAI7
DNALI1
DYNLT2B
TTC29
ZMYND12
DRC
DRC1
DRC7
CCDC65
DRC3
DRC4
EFCAB2
FBXL13
IQCA1
IQCD
IQCG
TCTE1
Others
GAS2L2
RPGR
CEP164
DAW1
LRRC56
OFD1
DNAAF
CCDC103
CFAP298
CFAP300
DNAAF1
DNAAF11
DNAAF2
DNAAF3
DNAAF4
DNAAF5
DNAAF6
SPAG1
TTC12
ZMYND10
Multiciliogenesis
CCNO
FOXJ1
MCIDAS
NEK10
RFX2
RFX3
TP73
Log2FC
0
-0.5
-1
-1.5
-2
-2.5
-3
-3.5

C

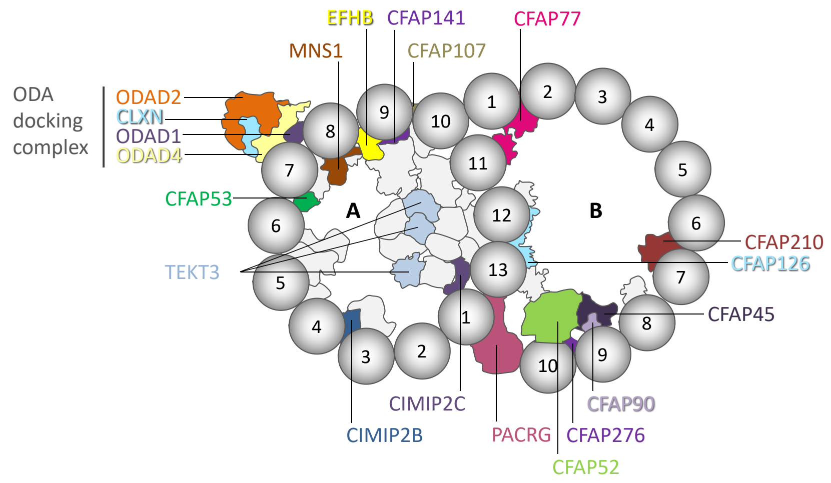
EFHB
CFAP141
CFAP77
MNS1
CFAP107
ODA
docking
complex
ODAD2
CLXN
ODAD1
ODAD4
CFAP53
TEKT3
A
B
CFAP210
CFAP126
CFAP45
CIMIP2C
CIMIP2B
PACRG
CFAP276
CFAP90
CFAP52

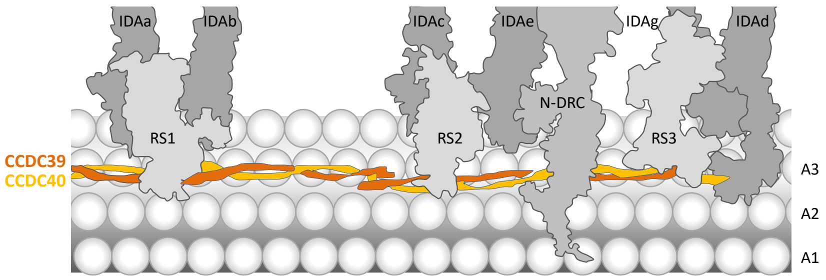
IDAa
IDAb
IDAc
IDAe
IDAg
IDAd
N-DRC
RS1
RS2
RS3
CCDC39
CCDC40
A3
A2
A1

Figure S7. Heatmap of transcript expression (log_2FC) in AECs from PCD patients heterozygous for a *FOXJ1* pathogenic variation compared to controls

RNA-seq expression analysis of a subset of 167 genes enriched in multiciliated cells encoding axoneme components or proteins involved in its assembly. Genes downregulated in patients are shown in blue, while those with unchanged expression appear in white. The heatmap was generated using RStudio.

A) The proteins encoded by these genes are categorized into two groups based on whether they interact with axonemal tubulin. Of the 167 proteins, 151 are represented in the heatmap (the interaction with axonemal tubulin remains unknown for 16). The presence of FOXJ1 ChIP-Seq peaks in gene promoters is indicated to the right of each gene (+). Among the 72 genes encoding proteins that directly bind to axonemal tubulin, 29 are downregulated in PCD patients heterozygous for a *FOXJ1* pathogenic variation. Of these, 21 (21/29) are directly regulated by FOXJ1, as evidenced by their capture in ChIP-Seq. In contrast, among the 79 proteins that do not bind to axonemal tubulin, only six exhibit downregulated transcripts in PCD patients with a heterozygous *FOXJ1* pathogenic variation, and three of these (3/6) are directly regulated by FOXJ1 (captured in ChIP-Seq). These highly significant findings ($\chi^2 = 21.15$; $p = 4.23 \times 10^{-6}$) suggest that FOXJ1 predominantly regulates the expression of proteins that directly interact with axonemal tubulin.

B) The proteins encoded by these genes are categorized according to their associated ciliary structure. CA: central apparatus; DNAAF: dynein axonemal assembly factor; DRC: dynein regulatory complex; IDA: inner dynein arm; MIA: modifier of inner arms; MIP: microtubule inner protein; ODA: outer dynein arm; RS: radial spoke; Ruler: CCDC39 and CCDC40 ruler; TUB: tubulin.

C) Schematic representation of axoneme cross-section (upper panel) adapted from Gui et al (25,30) and longitudinal section (lower panel) adapted from Leung et al (29). In color are proteins of docking complexes, MIPs and rulers, whose expression is downregulated in AECs from PCD patients carrying a heterozygous pathogenic variation in *FOXJ1*.
