# Supplementary material for: FOXJ1 transcriptional targets in human airway cells and impaired multiciliogenesis in FOXJ1-associated primary ciliary dyskinesia
Source: Am J Respir Cell Mol Biol. 2026 Mar 30;74(11):1553–65. doi: 10.1093/ajrcmb/aanag071 (PMC13720309; doi:10.1093/ajrcmb/aanag071)
Supplement: Supplementary file 1 [file aanag071_supplementary_data.zip › Supp text and table.pdf]

FOXJ1 transcriptional targets in human airway cells and impaired multiciliogenesis in *FOXJ1*-associated primary ciliary dyskinesia

Lucie Thomas, Jacques Serizay, Rahma Mani, Sandrine Couvet, Jean-François Papon, Aline Tamalet, Rana Mitri, Catherine Faucon, Marlene Murris-Espin, Emmanuelle Blanchard, Maud Langeois, Sophie Blesson, Sylvie Tissier, Guy Montantin, Florence Dastot-Le Moal, Romain Levergeois, Bruno Louis, Nicole Beydon, Guillaume Thouvenin, Estelle Escudier, Serge Amselem, Irina Giurgea, Laure-Emmanuelle Zaragosi, Alice Meunier, Marie Legendre

ONLINE DATA SUPPLEMENTAL TEXT

Material and methods

Genetic analyses

The identification of pathogenic variations in *FOXJ1* was performed on genomic DNA extracted from peripheral blood leukocytes. DNA samples underwent sequencing through next-generation sequencing (NGS), using a custom targeted capture panel (SeqCap EZ Choice, Roche Diagnostics) that encompasses 55 genes known to be involved in PCD. The libraries were prepared following the manufacturer's instructions and sequenced on a MiSeq system (Illumina). The data were analysed through an in-house double pipeline based on the Bowtie2 and BWA tools. The reads were visualized with the IGV viewer (Broad Institute). Copy number variation analysis was performed using a depth-ratio comparison between subjects sequenced in the same run. The DNA from individuals DCP521, 17GM00680 and 8821GM000400 was analysed with the aforementioned targeted panel, and the *FOXJ1* variants were confirmed by Sanger sequencing using the BigDye Terminator v3.1 system (ThermoFisher) as previously described (1) with amplification and sequencing primers listed in **Table S1**. The sequences were analysed with the SeqScape software (ThermoFisher).

Constructs and transfections

Total RNA was extracted from a control hAECs with RNeasy Mini Kit (Qiagen) before being retro-transcribed in cDNA with the Reverse Transcriptor kit (Roche) following the manufacturer's instructions. *FOXJ1* cDNA was amplified by PCR and cloned into pcDNA3.1/V5-His-TOPO using the TOPO TA cloning kit (Thermofischer Sc.) according to the manufacturer's recommendations to generate the plasmid p.cDNA3 FOXJ1 wt. Site directed mutagenesis was then performed on this plasmid with high fidelity polymerase (Q5 HF NEB) to generate two mutated plasmids: p.cDNA3-FOXJ1 867dup and p.cDNA3-FOXJ1 924del. Constructs were checked by Sanger sequencing before being transfected in HeLa cells with FuGENE® HD Transfection Reagent (Promega). All primers used are listed in **Table S1**.

Immunoblotting

HeLa cells and hAECs obtained from ALI cultures were suspended in ice-cold lysis buffer (50mM Tris, 150 mM NaCl, 1% Triton X-100, and Complete Protease Inhibitor Cocktail from Roche Diagnostics). The cell lysates were sonicated and cleared by centrifugation at 1000g. The cell lysates were loaded onto 10% SDS-PAGE gel, transferred on a PVDF membrane and analysed by western blotting with an anti-FOXJ1 antibody (Thermofischer 14-9965-82, 1/200) or an anti-LAMIN B1 antibody (SIGMA HPA050524, 1/500). All blots were incubated overnight at 4°C in PBS - 0.1% Tween 20 - 5% milk. Secondary antibodies (rabbit-HRP, SIGMA A0545, or mouse-HRP, SIGMA A9044) were incubated for 1h at room temperature at a final dilution of 1:5000 in PBS - 0.1% Tween 20 - 5% milk and revealed by Amersham ECL Select Western Blotting Detection Reagent (Cytiva) according to the manufacturer's recommendations.

Quantitative RT-PCR

One microgram of total RNA was extracted from ALI-cultured hAECs using the RNeasy Mini Kit (Qiagen). Reverse transcription was performed with the Reverse Transcriptor kit (Roche) following the manufacturer's instructions. Real-time PCR assays were performed in a LC480 Light Cycler device (Roche) with the MESA BLUE qPCR MasterMix Plus for SYBR Assay (Eurogentec). The ubiquitously expressed *ERCC3* gene was used as an endogenous control for data normalization. Corresponding primers are listed in **Table S1**.

Dephosphorylation assay

HeLa cells and hAECs obtained from ALI cultures were suspended in ice-cold lysis buffer (50mM Tris, 150 mM NaCl, 1% Triton X-100, and Complete Protease Inhibitor Cocktail from Roche Diagnostics). The lysates were sonicated and centrifuged at 1000g. Following quantification with the BCA Protein Assay Kit (Thermoscientific), total protein extracts were treated with Lambda Protein Phosphatase (NEB P0753S) in accordance with the manufacturer's instructions and resolved by immunoblotting.

RNA extraction and transcriptome analyses

RNA was extracted from hAECs of patients DCP521, 17GM00680 and three control individuals with RNeasy mini kit (QIAGEN). The RNA sequencing was performed by Integragen (Evry, France). The libraries were prepared with the TruSeq Stranded mRNA kit protocol according to supplier recommendations. In brief, polyA-containing mRNA molecules were isolated from 1µg total RNA using poly-T oligonucleotides attached to magnetic beads. The RNA matrices were fragmented using divalent cations under elevated temperature to obtain approximately 300bp-long fragments. Double-strand cDNAs were synthetized, Illumina adapters were ligated and the cDNA libraries were amplificated by PCR for sequencing on Illumina HiSeq4000.

Chromatin-Immunoprecipitation sequencing (ChIP-seq)

ChIP-seq was performed by Active Motif (Carlsbad, CA) following 5 steps: chromatin preparation, chIP reactions, library generation, sequencing, and basic data analysis. Firstly, control hAECs cultured at ALI for 3 months were fixed with 1% formaldehyde for 15 min and quenched with 0.125 M glycine. Chromatin was then isolated by adding lysis buffer, followed by disruption with a Dounce homogenizer. The lysates were sonicated and the DNA sheared to an average length of 300-500 bp with EpiShear probe sonicator (Active Motif). Genomic DNA (Input) was prepared by treating aliquots of chromatin with RNase, proteinase K and heat for de-crosslinking, followed by SPRI beads clean up (Beckman Coulter) and quantitation by Clariostar (BMG Labtech). Extrapolation to the original chromatin volume allowed determination of the total chromatin yield. An aliquot of chromatin (25 ug) was precleared with protein A agarose beads (Invitrogen). The genomic DNA regions of interest were isolated using 25ul of antibody against FOXJ1 (Thermo cat# 14-9965-82, lot# 2417646). The complexes were washed, eluted from the beads with SDS buffer, and subjected to RNase and proteinase K treatment. The crosslinks were reversed by incubation at 65°C overnight, and ChIP DNA was purified by phenol-chloroform extraction and ethanol precipitation. Quantitative PCR (QPCR) reactions were then carried out in triplicate on specific genomic regions using SYBR Green Supermix (Bio-Rad). The resulting signals were normalized for primer efficiency by carrying out QPCR for each primer pair using Input DNA. Illumina sequencing libraries were subsequently prepared using a custom type based on the paired read

adapter oligonucleotides previously described (2). The libraries were prepared from the ChIP and Input DNAs on an automated system (Apollo 342, Wafergen Biosystems/Takara). After a final PCR amplification step, the resulting DNA libraries were quantified and sequenced on an Illumina's NovaSeq platform (75 nt reads, single end).

Bioinformatic analyses

Transcriptome analyses

Sequencing, data quality, reads repartition (e.g., for potential adaptor contamination), and adaptor trimming were performed using FastQC and trim_galore. Paired-end reads were mapped using STARv2.4.0 (3) on the hg38 Human genome assembly. Samtools was used to identify and remove duplicates, remove reads with mapping quality lower than 20 and filter out pairs aligned discordantly.

Gene expression regulation study was performed as previously described (4). In brief, transcript abundance was estimated using featureCounts (5) with Gencode v41 human gene annotations. Transcript abundance across all samples and replicates was gathered into a single count matrix. Normalization and differential gene expression were performed using DESeq2 (6) on R (v.4.4.0). MA plot was generated using shrunken log2 fold change estimates from DESeq2, and differential expression was considered statistically significant for adjusted p-values ≤ 0.1 and absolute fold-changes ≥ 1.5.

ChIP-sequencing

Sequencing, data quality, read distribution and adaptor trimming were performed as described above. Single-end reads were mapped using bowtie2 (7) on the hg38 Human genome assembly. Samtools was used to mark and remove duplicates, remove reads with mapping quality lower than 10 and filter out unmapped reads. Alignments were extended in silico at their 3'-ends to a length of 169bp, which is the average genomic fragment length in the size-selected library, and genome coverage was generated and stored in bigWig files with deepTools (8).

The MACS algorithm (v2.1.0) was employed with default parameters to annotate 785 peaks. The five motifs (between 4 and 12 bp) most enriched in FOXJ1 peak sequences were identified *de novo* using XSTREME (9), with ` --meme-mod zoops` argument. All the sequences of FOXJ1 peaks

(including reverse complement sequences) were subsequently scanned using fimo (DOI: 10.1093/bioinformatics/btr064) to annotate those harboring FOX, RFX, both or no motifs (p-value ≤ 0.001). For each FOXJ1 peak with both FOX and RFX motifs, the enrichment of peaks with an inter-motif distance between -10 and -1 (i.e., RFX upstream of FOX) was measured using a binomial test.

Identification of direct FOXJ1 targets

Genes both differentially expressed in FOXJ1-deficient patients (measured by RNA-seq), and with a FOXJ1 peak within their gene body or up to 5kb upstream of their Transcription Start Site (TSS) were annotated as direct FOXJ1 targets. Other genes differentially expressed in FOXJ1-deficient patients but without a FOXJ1 peak were annotated as indirectly regulated genes. In parallel, differentially regulated genes with a multiciliated/secretory cell expression ratio > 4 in single-cell RNAseq analyses (10) were annotated as multiciliated cells-enriched genes, and those with a multiciliated/secretory cell expression ratio < 0.25 were annotated as secretory cells-enriched genes. Over-representation of biological functions within multiciliated cells-enriched down-regulated genes was performed using Metascape (11).

REFERENCES

1. Thomas L, Bouhouche K, Whitfield M, Thouvenin G, Coste A, Louis B, et al. TTC12 Loss-of-Function Mutations Cause Primary Ciliary Dyskinesia and Unveil Distinct Dynein Assembly Mechanisms in Motile Cilia Versus Flagella. The American Journal of Human Genetics. 2020 Feb;106(2):153–69.

2. Bentley DR, Balasubramanian S, Swerdlow HP, Smith GP, Milton J, Brown CG, et al. Accurate whole human genome sequencing using reversible terminator chemistry. Nature. 2008 Nov 6;456(7218):53–9.

3. Dobin A, Davis CA, Schlesinger F, Drenkow J, Zaleski C, Jha S, et al. STAR: ultrafast universal RNA-seq aligner. Bioinformatics. 2013 Jan 1;29(1):15–21.

4. Noli L, Capalbo A, Ogilvie C, Khalaf Y, Ilic D. Discordant Growth of Monozygotic Twins Starts at the Blastocyst Stage: A Case Study. Stem Cell Reports. 2015 Dec 8;5(6):946–53.

5. Liao Y, Smyth GK, Shi W. featureCounts: an efficient general purpose program for assigning sequence reads to genomic features. Bioinformatics. 2014 Apr 1;30(7):923–30.

6. Love MI, Huber W, Anders S. Moderated estimation of fold change and dispersion for RNA-seq data with DESeq2. Genome Biol. 2014;15(12):550.

7. Langmead B, Salzberg SL. Fast gapped-read alignment with Bowtie 2. Nat Methods. 2012 Apr;9(4):357–9.

8. Ramírez F, Dündar F, Diehl S, Grüning BA, Manke T. deepTools: a flexible platform for exploring deep-sequencing data. Nucleic Acids Research. 2014 July 1;42(W1):W187–91.

9. Grant CE, Bailey TL. XSTREME: Comprehensive motif analysis of biological sequence datasets [Internet]. 2021 [cited 2025 June 18]. Available from: http://biorxiv.org/lookup/doi/10.1101/2021.09.02.458722

10. Plasschaert LW, Žilionis R, Choo-Wing R, Savova V, Knehr J, Roma G, et al. A single-cell atlas of the airway epithelium reveals the CFTR-rich pulmonary ionocyte. Nature. 2018 Aug;560(7718):377–81.

11. Zhou Y, Zhou B, Pache L, Chang M, Khodabakhshi AH, Tanaseichuk O, et al. Metascape provides a biologist-oriented resource for the analysis of systems-level datasets. Nat Commun. 2019 Apr 3;10(1):1523.

Table S1: Primer sequences	
**Name**	**Sequence**
PCR amplification and Sanger sequencing	
FOXJ1_ex2F	AGGCCGTGTAACGGGAG
FOXJ1_ex2R	TGCAGGGCTCCCTTCTG
FOXJ1_ex3F	GTGGCTGGTGTCCCAGAG
FOXJ1_ex3R	TCCTGGGCTTGAATCTGATG
FOXJ1_ex3SeqF	GGAGCAGGGTGAGCTGGA
FOXJ1_ex3SeqR	CCTCCCAGTCAAAGTTGCC
FOXJ1_ex3SeqF2	CCTGGGCAGACCAGTGTGT
FOXJ1_ex3SeqR2	TGTCTGTGGACCTGTGTTGG
FOXJ1_F	ACCAGGTGCCAGGTTCAG
FOXJ1_F2	TGTCGGCCATCTACAAGTGG
FOXJ1_R	ATGGTGAGGTCCACGTCCA
Real-time PCR	
QPCR_FOXJ1-F	GGGGTGGGAGCAACTTCT
QPCR_FOXJ1-R	CCTCCTCCGAATAAGTATGTGGT
QPCR_CCNO-F	GCAGGAGAGCCACTTCCA
QPCR_CCNO-R	GCAGCTTACAGCGGGATTC
QPCR_MCIDAS-F	AGACGCGCTTGTTGAGAATAAT
QPCR_MCIDAS-R	TGTGTGATCATCAGCTTATCCAG
QPCR_ERCC3-F	ACTGGATGGAGCTGCAGAAT
QPCR_ERCC3-R	GACATAGGGCACCAGACCTC
Directed site mutagenesis	
FOXJ1_867dup_F	CGCGGCCCCCCCAGCA
FOXJ1_867dup_R	TGCTGGGGGGGCCGCG
FOXJ1_924del_F	TTTGACTGGGAGGCCATCTTCG
FOXJ1_924del_R	TTGCCTTTGAGGGGTTCCAGC

PCR: polymerase chain reaction

Table S2. Antibodies used for immunofluorescence				
**Antibody**	**Host**	**Company**	**Reference**	**Dilution**
**α-acetylated tubulin**	mouse	Sigma	(6-11-B1) T6793	1/600
**CEP164**	rabbit	Sigma	HPA037606	1/200
**DNAH5**	rabbit	Sigma	HPA037470	1/200
**DNAH9**	rabbit	Sigma	HPA052641	1/150
**DNALI1**	rabbit	Sigma	HPA028305	1/150
**FOXJ1**	mouse	ThermoFisher	14-9965-82	1/100
**GAS2L2**	rabbit	Sigma	HPA041311	1/400
**ODAD2/ARMC4**	rabbit	Sigma	HPA037829	1/400
**ROOTLETIN**	rabbit	Sigma	HPA021191	1/200
**Rabbit A488**	goat	LifeTechnologies	A11034	1/500
**Mouse A594**	goat	LifeTechnologies	A11032	1/500

Table S3. Differentially expressed genes directly or undirectly regulated by FOXJ1

HGNC ID	gene_name	log2FoldChange	direction	foxj1_peak	CELL TYPE
HGNC:23336	A2ML1	2,56737587	up	FALSE	OTHER
HGNC:42	ABCB11	1,71902068	up	FALSE	OTHER
HGNC:19837	ABHD12B	1,33646824	up	FALSE	CILIATED
HGNC:29132	ABLIM3	2,8443086	up	FALSE	OTHER
HGNC:14899	ADAMTS14	-3,1872441	down	FALSE	OTHER
HGNC:219	ADAMTS3	-3,3159961	down	FALSE	OTHER
HGNC:13202	ADAMTS9	-4,0847601	down	FALSE	OTHER
HGNC:21212	ADGB	-0,823299	down	TRUE	CILIATED
HGNC:255	ADH6	-2,6264689	down	FALSE	CILIATED
HGNC:7137	AFDN	-1,1037821	down	TRUE	OTHER
HGNC:24061	AKAP14	-2,4997166	down	FALSE	CILIATED
HGNC:376	AKAP6	-0,9931515	down	FALSE	OTHER
HGNC:19705	ANGPTL5	-2,0178412	down	TRUE	OTHER
HGNC:32525	ANKDD1B	-1,3694329	down	FALSE	CILIATED
HGNC:25691	ANKRD53	-2,2830083	down	FALSE	OTHER
HGNC:44669	ANKRD66	-1,3878902	down	FALSE	CILIATED
HGNC:24600	ANKS1B	-2,2338122	down	FALSE	OTHER
HGNC:534	ANXA10	5,98930302	up	FALSE	OTHER
HGNC:581	APBB1	-1,7433121	down	FALSE	CILIATED
HGNC:32152	APOBEC4	-1,740006	down	TRUE	CILIATED
HGNC:613	APOE	3,06090581	up	FALSE	OTHER
HGNC:16029	AQP10	-2,9068002	down	FALSE	CILIATED
HGNC:21035	ARHGAP18	-1,8752402	down	TRUE	CILIATED
HGNC:676	ARHGAP6	-1,0499158	down	FALSE	OTHER
HGNC:30964	ARMC3	-1,2455038	down	TRUE	CILIATED
HGNC:727	ARTN	-2,1595452	down	FALSE	SECRETED
HGNC:18060	ARX	-1,7926711	down	FALSE	CILIATED
HGNC:21899	ATG9B	-1,2289092	down	TRUE	CILIATED
HGNC:13543	ATP10B	1,70812525	up	FALSE	SECRETED
HGNC:21649	BAIAP2L1	-0,8597352	down	TRUE	OTHER
HGNC:948	BAIAP3	-1,1118071	down	TRUE	CILIATED
HGNC:1033	BDNF	2,50878413	up	FALSE	OTHER
HGNC:17106	BEST4	-2,347965	down	FALSE	CILIATED
HGNC:19351	BICC1	-6,4576639	down	FALSE	OTHER
HGNC:16108	BPIFB1	1,71482067	up	FALSE	SECRETED
HGNC:28716	C10orf67	-0,9818863	down	TRUE	CILIATED
HGNC:31428	C10orf99	3,77627766	up	FALSE	OTHER
HGNC:1169	C11orf16	-1,3150563	down	FALSE	CILIATED
HGNC:32044	C1orf141	-1,2005111	down	FALSE	CILIATED
HGNC:28547	C1orf87	-3,572423	down	FALSE	CILIATED
HGNC:31374	C1QTNF8	-2,6872537	down	FALSE	OTHER
HGNC:16216	C20orf85	-1,7295228	down	TRUE	CILIATED
HGNC:26324	C2orf50	-1,2065292	down	TRUE	CILIATED
HGNC:1318	C3	1,33597771	up	FALSE	SECRETED
HGNC:27028	C5orf49	-0,9454297	down	TRUE	CILIATED

HGNC:1339	C6	-3,3758098	down	FALSE	CILIATED
HGNC:21233	C6orf118	-1,1422893	down	FALSE	CILIATED
HGNC:20881	C6orf52	-1,456422	down	FALSE	CILIATED
HGNC:22247	C7orf57	-1,2288942	down	TRUE	CILIATED
HGNC:30905	C8orf34	-5,4941948	down	FALSE	OTHER
HGNC:19919	C9orf24	-1,2632036	down	TRUE	CILIATED
HGNC:28337	C9orf72	-0,9142192	down	TRUE	CILIATED
HGNC:1482	CAPN5	1,00621617	up	FALSE	OTHER
HGNC:28375	CAPSL	-1,2144556	down	TRUE	CILIATED
HGNC:25062	CATIP	-1,0931009	down	TRUE	CILIATED
HGNC:28598	CATSPERD	-6,9030538	down	FALSE	CILIATED
HGNC:25002	CCDC113	-1,1778987	down	FALSE	CILIATED
HGNC:29296	CCDC146	-0,7549198	down	TRUE	CILIATED
HGNC:23813	CCDC3	2,48814439	up	FALSE	OTHER
HGNC:26552	CCDC33	-1,5248103	down	TRUE	CILIATED
HGNC:25244	CCDC39	-1,1049222	down	TRUE	CILIATED
HGNC:26090	CCDC40	-1,4800059	down	TRUE	CILIATED
HGNC:25367	CCDC8	-2,2122962	down	TRUE	OTHER
HGNC:26900	CCDC96	-0,944654	down	TRUE	CILIATED
HGNC:24235	CD200R1	2,3410335	up	FALSE	SECRETED
HGNC:1678	CD4	-2,1094121	down	FALSE	CILIATED
HGNC:26308	CDHR3	-1,0878548	down	TRUE	CILIATED
HGNC:34527	CDHR4	-1,2728302	down	FALSE	CILIATED
HGNC:1781	CDKL1	-1,3417505	down	FALSE	CILIATED
HGNC:2550	CELF2	1,62102858	up	FALSE	SECRETED
HGNC:17966	CEP83	-1,3076033	down	TRUE	CILIATED
HGNC:26741	CES4A	-1,9448863	down	FALSE	CILIATED
HGNC:28567	CFAP107	-1,9179721	down	TRUE	CILIATED
HGNC:32325	CFAP126	-0,7912868	down	TRUE	CILIATED
HGNC:32305	CFAP141	-3,4778397	down	TRUE	CILIATED
HGNC:27843	CFAP157	-1,1842272	down	TRUE	CILIATED
HGNC:25064	CFAP210	-1,0674164	down	TRUE	CILIATED
HGNC:33720	CFAP221	-0,778217	down	FALSE	CILIATED
HGNC:32331	CFAP276	-2,0653574	down	FALSE	CILIATED
HGNC:26684	CFAP43	-1,0721656	down	TRUE	CILIATED
HGNC:17229	CFAP45	-1,0493407	down	TRUE	CILIATED
HGNC:25247	CFAP46	-1,2692064	down	TRUE	CILIATED
HGNC:16053	CFAP52	-0,9701931	down	TRUE	CILIATED
HGNC:26530	CFAP53	-1,1466756	down	TRUE	CILIATED
HGNC:26676	CFAP58	-0,9598037	down	FALSE	CILIATED
HGNC:25325	CFAP65	-0,8228829	down	TRUE	CILIATED
HGNC:37100	CFAP73	-1,0086422	down	TRUE	CILIATED
HGNC:33776	CFAP77	-0,7919937	down	TRUE	CILIATED
HGNC:53789	CFAP97D2	-4,3329316	down	FALSE	CILIATED
HGNC:51180	CFAP99	-1,2000167	down	TRUE	CILIATED
HGNC:1972	CHST4	1,80522881	up	FALSE	SECRETED
HGNC:2018	CLCA4	1,58904277	up	FALSE	SECRETED
HGNC:25678	CLXN	-0,8941274	down	FALSE	CILIATED

HGNC:2152	CNGA4	-1,4030609	down	FALSE	OTHER
HGNC:2155	CNN1	-3,8242277	down	FALSE	OTHER
HGNC:2169	CNTF	-3,7687976	down	FALSE	CILIATED
HGNC:13834	CNTNAP3	1,18368306	up	FALSE	OTHER
HGNC:2194	COL17A1	2,64052422	up	FALSE	OTHER
HGNC:22442	COL28A1	-4,7548728	down	FALSE	OTHER
HGNC:2209	COL5A1	-2,8949513	down	FALSE	OTHER
HGNC:2226	COLQ	-1,4896068	down	TRUE	OTHER
HGNC:2227	COMP	6,02258461	up	FALSE	SECRETED
HGNC:21744	CPEB1	-2,8528734	down	FALSE	OTHER
HGNC:14399	CPVL	-2,1933659	down	FALSE	OTHER
HGNC:29219	CRACD	-1,1817748	down	FALSE	CILIATED
HGNC:28657	CRACR2A	1,2290015	up	FALSE	OTHER
HGNC:12024	CRISP2	-2,6641126	down	FALSE	OTHER
HGNC:51677	CROCC2	-1,8979557	down	TRUE	CILIATED
HGNC:2418	CRYM	2,02109046	up	FALSE	OTHER
HGNC:14026	CSMD1	-3,1509446	down	FALSE	CILIATED
HGNC:26193	CSPP1	-1,003176	down	TRUE	CILIATED
HGNC:2478	CST6	-3,2812956	down	FALSE	CILIATED
HGNC:26133	CWH43	-2,7868509	down	FALSE	CILIATED
HGNC:18141	DCDC2	-2,3697516	down	TRUE	CILIATED
HGNC:32576	DCDC2B	-3,0726795	down	FALSE	CILIATED
HGNC:2709	DCT	-4,5065028	down	FALSE	OTHER
HGNC:18104	DEFB124	-4,2831915	down	FALSE	OTHER
HGNC:16940	DGAT2	1,25537529	up	FALSE	OTHER
HGNC:2846	DGCR6	-1,0524129	down	FALSE	OTHER
HGNC:2855	DGKI	-1,7096236	down	FALSE	OTHER
HGNC:16888	DHRS9	0,9748026	up	FALSE	OTHER
HGNC:2899	DLEC1	-1,3048749	down	FALSE	CILIATED
HGNC:2928	DMD	-1,5944819	down	FALSE	OTHER
HGNC:13909	DMRT3	-1,4892565	down	FALSE	OTHER
HGNC:2948	DNAH2	-1,224612	down	FALSE	CILIATED
HGNC:18744	DNAI2	-1,9957523	down	TRUE	CILIATED
HGNC:26252	DNAI4	-1,1371506	down	FALSE	CILIATED
HGNC:2985	DOC2A	-1,1426482	down	FALSE	CILIATED
HGNC:3013	DPYS	-5,6905767	down	FALSE	OTHER
HGNC:24245	DRC1	-1,7044362	down	TRUE	CILIATED
HGNC:25289	DRC7	-0,639262	down	TRUE	CILIATED
HGNC:3023	DRD2	-2,0906266	down	FALSE	OTHER
HGNC:37261	DTHD1	-0,88249	down	TRUE	CILIATED
HGNC:32315	DYNLT4	-1,7570607	down	TRUE	CILIATED
HGNC:26882	DYNLT5	-3,0150236	down	FALSE	CILIATED
HGNC:26551	DZIP1L	-1,1102742	down	TRUE	CILIATED
HGNC:34531	EFCAB10	-1,3595285	down	TRUE	CILIATED
HGNC:26330	EFHB	-2,5939427	down	TRUE	CILIATED
HGNC:25334	ELMOD1	-3,1220253	down	FALSE	OTHER
HGNC:31670	ENO4	-1,0028024	down	TRUE	CILIATED
HGNC:25474	ENOX1	-4,0835302	down	FALSE	CILIATED

HGNC:3357	ENPP2	-4,2613678	down	FALSE	OTHER
/	ENSG00000188897/LOC400499	-1,5723476	down	FALSE	OTHER
HGNC:15932	EPPIN	-4,3601231	down	FALSE	CILIATED
HGNC:38825	EPPIN-WFDC6	-6,6778131	down	FALSE	OTHER
HGNC:3542	F5	-2,1422142	down	FALSE	OTHER
HGNC:3561	FABP6	-6,8875673	down	FALSE	CILIATED
HGNC:28587	FAM110B	-3,1668358	down	FALSE	OTHER
HGNC:26413	FAM124A	-4,9820883	down	FALSE	OTHER
HGNC:19371	FAM13C	-2,1692325	down	FALSE	OTHER
HGNC:34242	FAM166B	-0,9806809	down	FALSE	CILIATED
HGNC:27938	FAM166C	-1,742524	down	FALSE	CILIATED
HGNC:20991	FAM184A	-1,9598483	down	FALSE	CILIATED
HGNC:26883	FAM216B	-1,5398069	down	TRUE	CILIATED
HGNC:26335	FAM81B	-1,3540467	down	TRUE	CILIATED
HGNC:16121	FAM83C	1,40990666	up	FALSE	OTHER
HGNC:16122	FAM83D	1,00511438	up	FALSE	OTHER
HGNC:25531	FAR2	2,11179351	up	FALSE	OTHER
HGNC:3601	FBLN2	2,80304119	up	FALSE	SECRETED
HGNC:13617	FBXO15	-1,21665	down	TRUE	CILIATED
HGNC:3671	FGF14	-3,2388721	down	FALSE	OTHER
HGNC:29408	FHAD1	-1,3214759	down	TRUE	CILIATED
HGNC:21015	FILIP1	-1,5247252	down	FALSE	CILIATED
HGNC:14074	FMN2	-4,3262712	down	FALSE	OTHER
HGNC:13875	FOXP2	-0,8028689	down	FALSE	OTHER
HGNC:28572	FRMPD2	-3,3215129	down	TRUE	CILIATED
HGNC:4013	FUT2	1,03488778	up	FALSE	OTHER
HGNC:4092	GAD1	-1,6409736	down	FALSE	CILIATED
HGNC:24846	GAS2L2	-2,5303584	down	FALSE	CILIATED
HGNC:4183	GBP2	1,22149678	up	FALSE	OTHER
HGNC:18177	GIPC2	-3,5696946	down	FALSE	CILIATED
HGNC:4278	GJA4	-5,1917407	down	FALSE	OTHER
HGNC:4376	GMPR	-1,6303955	down	FALSE	CILIATED
HGNC:19991	GPR135	-1,138951	down	TRUE	CILIATED
HGNC:16693	GPR162	-2,8346473	down	FALSE	CILIATED
HGNC:4475	GPR20	2,99951424	up	FALSE	OTHER
HGNC:4597	GRM5	-3,4007525	down	FALSE	OTHER
HGNC:4628	GSTA3	-4,4762489	down	FALSE	CILIATED
HGNC:13242	HHATL	-2,3343326	down	FALSE	OTHER
HGNC:4942	HLA-DQA1	2,78002813	up	FALSE	OTHER
HGNC:4947	HLA-DRA	0,89969653	up	FALSE	OTHER
HGNC:11630	HNF1B	-2,1630643	down	FALSE	OTHER
HGNC:25061	HOATZ	-1,3557412	down	FALSE	CILIATED
HGNC:5225	HSF2	-0,8878862	down	FALSE	OTHER
HGNC:5362	ID3	1,82874075	up	FALSE	OTHER
HGNC:5466	IGF2	4,48454941	up	FALSE	OTHER
HGNC:28868	IGF2BP3	2,33842524	up	FALSE	OTHER
HGNC:16830	IL32	-2,3768373	down	FALSE	OTHER
HGNC:6017	IL5RA	-1,1439418	down	TRUE	CILIATED

HGNC:14359	IRX2	-5,7171303	down	FALSE	OTHER
HGNC:14675	IRX6	-2,0601386	down	FALSE	SECRETED
HGNC:6173	ITM2A	-3,6025554	down	FALSE	OTHER
HGNC:26288	JHY	-1,1087688	down	FALSE	OTHER
HGNC:24796	KANK3	2,28540666	up	FALSE	OTHER
HGNC:6217	KATNB1	-0,7179514	down	TRUE	CILIATED
HGNC:6231	KCNB1	-2,9253512	down	TRUE	OTHER
HGNC:6262	KCNJ16	-2,5346735	down	FALSE	CILIATED
HGNC:6286	KCNMB2	-3,0194749	down	FALSE	OTHER
HGNC:6289	KCNMB4	2,87465364	up	FALSE	OTHER
HGNC:18893	KCNRG	-2,7299617	down	TRUE	CILIATED
HGNC:14678	KCTD12	-1,4826764	down	TRUE	OTHER
HGNC:51250	KIAA2012	-2,4018838	down	TRUE	CILIATED
HGNC:19349	KIF21A	-0,9497666	down	FALSE	CILIATED
HGNC:25573	KLHDC8A	-1,5339895	down	FALSE	OTHER
HGNC:21221	KLHL32	-2,2062684	down	FALSE	CILIATED
HGNC:18653	KLHL6	-1,3994873	down	FALSE	CILIATED
HGNC:26488	KNCN	-5,2904274	down	FALSE	SECRETED
HGNC:6416	KRT14	4,9231688	up	FALSE	CILIATED
HGNC:26707	KRT40	3,74684269	up	FALSE	SECRETED
HGNC:29612	LAPTM5	3,33811583	up	FALSE	OTHER
HGNC:29471	LAYN	1,16394443	up	FALSE	OTHER
HGNC:33874	LGALS9C	1,50534726	up	FALSE	OTHER
HGNC:33718	LKAAEAR1	-3,2626185	down	FALSE	CILIATED
HGNC:26683	LMNTD1	-1,5845862	down	FALSE	CILIATED
HGNC:29480	LRG1	0,73862571	up	FALSE	OTHER
HGNC:23199	LRRC18	-1,6479681	down	TRUE	CILIATED
HGNC:19138	LRRC23	-0,8088966	down	TRUE	CILIATED
HGNC:28562	LRRC43	-1,3511339	down	TRUE	CILIATED
HGNC:25047	LRRC46	-0,877149	down	TRUE	CILIATED
HGNC:26556	LRRC71	-1,1766921	down	FALSE	CILIATED
HGNC:34301	LRRC74B	-1,4859045	down	TRUE	CILIATED
HGNC:6705	LSAMP	-1,8359542	down	FALSE	CILIATED
HGNC:23206	LUZP2	2,92777222	up	FALSE	OTHER
HGNC:6740	LYZ	2,12938861	up	FALSE	SECRETED
HGNC:6816	MAK	-1,1204819	down	TRUE	CILIATED
HGNC:6834	MAOB	-1,9792676	down	FALSE	OTHER
HGNC:26249	MAP3K19	-1,3531009	down	FALSE	CILIATED
HGNC:6882	MAPK8IP1	0,93815765	up	FALSE	CILIATED
HGNC:26655	MARCHF10	-1,7697957	down	TRUE	CILIATED
HGNC:6903	MAT1A	-1,764067	down	FALSE	CILIATED
HGNC:15505	MBOAT7	0,75725179	up	TRUE	OTHER
HGNC:6934	MCAM	2,00760309	up	FALSE	OTHER
HGNC:7043	MGAM	1,90450297	up	FALSE	OTHER
HGNC:7105	MITF	0,92438111	up	FALSE	OTHER
HGNC:7125	MLF1	-1,1574562	down	TRUE	CILIATED
HGNC:12744	MLXIPL	-2,3225575	down	FALSE	OTHER
HGNC:14668	MMEL1	1,3635028	up	FALSE	SECRETED

HGNC:14366	MMP28	1,73580831	up	FALSE	OTHER
HGNC:29636	MNS1	-1,191543	down	TRUE	CILIATED
HGNC:26287	MROH9	-6,2910773	down	FALSE	CILIATED
HGNC:7371	MSLN	0,90528335	up	FALSE	OTHER
HGNC:7372	MSMB	2,94955039	up	FALSE	SECRETED
HGNC:7516	MUC5B	2,63965205	up	FALSE	SECRETED
HGNC:19677	MYCBPAP	-1,0267212	down	FALSE	CILIATED
HGNC:23172	MYCT1	-8,3364792	down	FALSE	OTHER
HGNC:7590	MYLK	-2,9120751	down	FALSE	CILIATED
HGNC:29826	MYLK3	-2,6332384	down	TRUE	OTHER
HGNC:14083	MYO15B	-1,5088822	down	FALSE	OTHER
HGNC:7656	NCAM1	-2,1433754	down	FALSE	OTHER
HGNC:33739	NCCRP1	2,36714577	up	FALSE	SECRETED
HGNC:14466	NDRG4	-2,2266437	down	TRUE	OTHER
HGNC:7751	NELL2	-1,9023686	down	FALSE	OTHER
HGNC:7876	NOS3	-1,6924366	down	TRUE	OTHER
HGNC:37454	NPIPB6	-2,5948341	down	FALSE	OTHER
HGNC:8021	NT5E	1,64086225	up	FALSE	OTHER
HGNC:25208	NTN5	-1,5207359	down	FALSE	OTHER
HGNC:26560	ODAD1	-1,2958294	down	TRUE	CILIATED
HGNC:25583	ODAD2	-1,9087986	down	TRUE	CILIATED
HGNC:25280	ODAD4	-0,9066665	down	FALSE	CILIATED
HGNC:34388	ODF3B	-0,6902205	down	TRUE	CILIATED
HGNC:8155	OPRL1	-2,4863387	down	FALSE	OTHER
HGNC:27346	OTUD1	-1,5634651	down	FALSE	OTHER
HGNC:19152	PACRG	-0,9188003	down	TRUE	CILIATED
HGNC:8596	PAM	1,13768373	up	FALSE	SECRETED
HGNC:26876	PARP15	2,81757155	up	FALSE	OTHER
HGNC:8620	PAX6	-0,9686247	down	FALSE	OTHER
HGNC:8779	PDE3B	1,87080035	up	FALSE	OTHER
HGNC:8968	PIGR	0,96665617	up	FALSE	SECRETED
HGNC:17644	PIWIL2	-3,8940463	down	FALSE	OTHER
HGNC:17661	PLA1A	-1,0172672	down	FALSE	CILIATED
HGNC:17824	PLAAT2	-1,7855595	down	TRUE	CILIATED
HGNC:9051	PLAT	2,55546892	up	FALSE	SECRETED
HGNC:15917	PLCB1	2,10474612	up	FALSE	OTHER
HGNC:9093	PLTP	-1,2138511	down	FALSE	CILIATED
HGNC:21185	PNLDC1	-3,0448924	down	FALSE	OTHER
HGNC:16953	POSTN	-1,6008136	down	FALSE	SECRETED
HGNC:9213	POU2F2	-1,5794355	down	FALSE	OTHER
HGNC:21557	PPIL6	-1,0618002	down	TRUE	CILIATED
HGNC:9287	PPP1R1B	2,4803994	up	FALSE	OTHER
HGNC:28869	PPP1R32	-2,2643504	down	TRUE	CILIATED
HGNC:9306	PPP2R2C	2,77156835	up	FALSE	OTHER
HGNC:9364	PRG4	3,45094491	up	FALSE	OTHER
HGNC:20340	PRICKLE2	-1,1319581	down	FALSE	OTHER
HGNC:9402	PRKCG	1,88502338	up	FALSE	OTHER
HGNC:9446	PRLR	-5,0420395	down	FALSE	OTHER

HGNC:5188	PRMT8	-3,2767518	down	TRUE	OTHER
HGNC:9456	PROS1	-2,4992032	down	TRUE	CILIATED
HGNC:28574	PRR18	-2,8566644	down	TRUE	CILIATED
HGNC:25673	PRR29	-1,1292144	down	FALSE	CILIATED
HGNC:9586	PTCH2	-1,3670347	down	FALSE	OTHER
HGNC:9594	PTGER2	-1,8269561	down	FALSE	OTHER
HGNC:9600	PTGFR	1,97683065	up	FALSE	SECRETED
HGNC:9682	PTPRT	-3,6054617	down	FALSE	OTHER
HGNC:9725	PYGL	1,30246419	up	FALSE	OTHER
HGNC:9778	RAB3B	-2,2681937	down	FALSE	OTHER
HGNC:15922	REM1	-2,8578586	down	FALSE	OTHER
HGNC:24499	RGS22	-1,1157445	down	FALSE	CILIATED
HGNC:13872	RIPOR2	-1,6134294	down	TRUE	CILIATED
HGNC:10263	RP1	-0,9450753	down	TRUE	CILIATED
HGNC:10294	RPE65	-2,6116213	down	FALSE	OTHER
HGNC:10295	RPGR	-1,2106883	down	FALSE	CILIATED
HGNC:30908	RSAD2	-1,6549276	down	FALSE	OTHER
HGNC:27362	RSPH10B	-1,3846092	down	FALSE	CILIATED
HGNC:10473	RUNX3	-1,5345977	down	FALSE	OTHER
HGNC:1348	SAMD9	1,17125362	up	FALSE	OTHER
HGNC:19869	SCARF2	-2,9582343	down	FALSE	CILIATED
HGNC:18384	SCGB3A1	4,56325567	up	FALSE	SECRETED
HGNC:10583	SCN11A	-2,4736822	down	FALSE	OTHER
HGNC:35414	SDR42E2	-1,8927164	down	FALSE	CILIATED
HGNC:8941	SERPINA1	1,38872853	up	FALSE	OTHER
HGNC:14221	SERPINB11	2,12039916	up	FALSE	OTHER
HGNC:16087	SFXN3	-1,1222806	down	FALSE	CILIATED
HGNC:24699	SH3RF3	-4,2901665	down	FALSE	OTHER
HGNC:14295	SHANK2	-1,1276476	down	TRUE	OTHER
HGNC:37231	SHISA9	-2,1146537	down	FALSE	OTHER
HGNC:30553	SIAH3	-3,0765965	down	FALSE	OTHER
HGNC:15605	SKAP1	-4,3010154	down	FALSE	CILIATED
HGNC:23520	SLC16A9	1,91067769	up	FALSE	SECRETED
HGNC:10941	SLC1A3	1,8183431	up	FALSE	OTHER
HGNC:11003	SLC29A1	-1,6369231	down	FALSE	OTHER
HGNC:11020	SLC34A2	3,64920564	up	FALSE	OTHER
HGNC:20644	SLC37A2	1,30354365	up	FALSE	OTHER
HGNC:19429	SLC41A1	-0,9346901	down	FALSE	OTHER
HGNC:19119	SLC5A8	-1,7992227	down	FALSE	OTHER
HGNC:13622	SLC6A16	-3,422269	down	FALSE	OTHER
HGNC:10961	SLCO1B3	1,44914103	up	FALSE	OTHER
HGNC:26481	SLFN13	-0,8515806	down	FALSE	CILIATED
HGNC:11085	SLIT1	-2,9334774	down	FALSE	OTHER
HGNC:11118	SMN2	-1,1523305	down	FALSE	OTHER
HGNC:11139	SNCAIP	-3,0652872	down	TRUE	OTHER
HGNC:11204	SOX9	-1,7951362	down	FALSE	CILIATED
HGNC:11215	SPAG6	-1,2065348	down	TRUE	CILIATED
HGNC:22957	SPATS1	-2,6832967	down	TRUE	CILIATED

HGNC:15874	SPEF1	-0,9917545	down	FALSE	CILIATED
HGNC:11285	SRD5A2	-2,7680096	down	FALSE	CILIATED
HGNC:18080	ST3GAL6	-1,6261893	down	TRUE	OTHER
HGNC:11356	STAG3	-3,71344	down	FALSE	OTHER
HGNC:24133	STAP1	-1,6816543	down	FALSE	OTHER
HGNC:19162	STARD9	-2,502581	down	FALSE	OTHER
HGNC:44668	STMND1	-0,961764	down	FALSE	CILIATED
HGNC:19420	STOML3	-2,0679153	down	FALSE	CILIATED
HGNC:30650	STRA6	2,00205934	up	FALSE	OTHER
HGNC:17089	SYNE1	-1,340543	down	FALSE	CILIATED
HGNC:19861	SYNE3	-2,3714111	down	FALSE	OTHER
HGNC:23142	SYT16	2,89699581	up	FALSE	OTHER
HGNC:24119	SYT17	0,83547028	up	FALSE	OTHER
HGNC:11595	TBX18	-2,0312966	down	FALSE	OTHER
HGNC:14293	TEKT3	-3,0859364	down	FALSE	CILIATED
HGNC:28622	TEX26	-2,5459376	down	FALSE	CILIATED
HGNC:11757	TFF3	1,7212872	up	FALSE	OTHER
HGNC:11760	TFPI	1,36207999	up	FALSE	OTHER
HGNC:11773	TGFBR2	0,92286448	up	FALSE	OTHER
HGNC:11778	TGM2	3,15283928	up	FALSE	SECRETED
HGNC:11822	TIMP3	0,75700102	up	FALSE	OTHER
HGNC:28517	TMEM178A	-1,1458969	down	FALSE	OTHER
HGNC:34295	TMEM212	-1,4909798	down	TRUE	CILIATED
HGNC:18515	TMEM47	-4,385609	down	FALSE	OTHER
HGNC:28279	TNFAIP8L1	-0,9030138	down	TRUE	CILIATED
HGNC:11908	TNFRSF11A	1,03997874	up	FALSE	OTHER
HGNC:33715	TOGARAM2	-1,2687877	down	TRUE	CILIATED
HGNC:24164	TPPP	-1,5291912	down	TRUE	CILIATED
HGNC:14215	TRIM55	-3,6449468	down	FALSE	CILIATED
HGNC:26725	TRMT9B	-1,3694	down	FALSE	OTHER
HGNC:34348	TRNP1	1,60275452	up	FALSE	CILIATED
HGNC:14006	TRPV6	-1,9480741	down	FALSE	OTHER
HGNC:17753	TSPAN5	0,9347453	up	FALSE	OTHER
HGNC:11855	TSPAN8	2,16973992	up	FALSE	SECRETED
HGNC:25660	TTC21B	-1,1494543	down	FALSE	OTHER
HGNC:26693	TTLL10	-1,2097669	down	TRUE	CILIATED
HGNC:12406	TUB	-1,0526746	down	TRUE	CILIATED
HGNC:18637	TUBA4B	-0,8489482	down	TRUE	CILIATED
HGNC:21381	UBE3D	-1,2827342	down	FALSE	OTHER
HGNC:17781	UCN3	-4,2570149	down	FALSE	OTHER
HGNC:12560	UMODL1	-1,3219451	down	FALSE	OTHER
HGNC:26444	UROC1	-6,675259	down	FALSE	OTHER
HGNC:12601	USH2A	2,1225744	up	FALSE	OTHER
HGNC:2464	VCAN	-3,4827979	down	FALSE	CILIATED
HGNC:30387	VMO1	1,02446175	up	FALSE	SECRETED
HGNC:27879	VSIG10L2	1,9657482	up	FALSE	OTHER
HGNC:17149	VSIG2	1,37651135	up	FALSE	OTHER
HGNC:29323	WDFY4	-2,5047418	down	TRUE	OTHER

HGNC:23745	WDR38	-2,5122891	down	FALSE	CILIATED
HGNC:26587	WDR49	-1,0017193	down	FALSE	CILIATED
HGNC:28020	WDR86	-2,0349549	down	TRUE	CILIATED
HGNC:16164	WFDC6	-3,9471314	down	FALSE	CILIATED
HGNC:12736	WIPF1	-2,3886462	down	FALSE	OTHER
HGNC:26245	ZBBX	-0,9789057	down	TRUE	CILIATED
HGNC:12930	ZBTB16	-1,525591	down	FALSE	OTHER
HGNC:20354	ZC2HC1C	-0,8565357	down	FALSE	CILIATED
HGNC:20342	ZDHHC15	-3,9071126	down	FALSE	OTHER
HGNC:29377	ZMAT1	-1,3306189	down	FALSE	OTHER
HGNC:22785	ZNF727	-3,8544274	down	FALSE	OTHER
HGNC:21767	ZNF853	-1,7184202	down	FALSE	OTHER

Table S4. 785 FOXJ1 chIP peaks

chr	start	end	width	score	signalValue	pValue	qValue	peak	Genes
chr1	1116255	1116529	275	316	5.48629	37.89333	31.64858	148	C1orf159
chr1	1173725	1174070	346	281	7.95438	34.20718	28.15143	152	TTLL10-AS1,TTLL10
chr1	2322560	2322856	297	134	4.9409	18.61486	13.40676	166	ENSG00000287356,MORN1
chr1	2801635	2801862	228	76	3.83126	12.36897	7.63095	112	TTC34
chr1	3668933	3669193	261	140	4.25484	19.27062	14.02367	119	TP73,TP73-AS3
chr1	10561630	10561805	176	87	4.33898	13.57186	8.74524	105	PEX14
chr1	10643795	10644101	307	294	6.85594	35.58816	29.46319	148	CASZ1,ENSG00000272078
chr1	12746087	12746327	241	98	5.78926	14.73872	9.81891	119	CFAP107
chr1	15247213	15247404	192	96	3.8995	14.51531	9.60908	60	FHAD1
chr1	16926710	16926890	181	111	4.46419	16.18254	11.15642	74	CROCC
chr1	22185105	22185567	463	107	4.50171	15.68827	10.70351	318	NA
chr1	23013763	23014094	332	133	4.41943	18.50548	13.31109	194	TEX46
chr1	23793430	23793750	321	177	5.59969	23.28478	17.78892	153	ENSG00000289835,LYPLA2
chr1	25198703	25198877	175	77	3.72815	12.49725	7.7496	86	NA
chr1	26306457	26306773	317	241	5.96504	30.02166	24.17332	168	UBXN11
chr1	27234190	27234362	173	80	3.31462	12.80328	8.04025	68	WDTC1-DT,WDTC1
chr1	27573171	27573351	181	39	3.70326	8.33456	3.96937	49	AHDC1
chr1	36083901	36084431	531	215	7.67317	27.22499	21.51246	207	ENSG00000232862,TEKT2,ADPRS
chr1	36207405	36207580	176	120	4.38058	17.10061	12.01049	103	NA
chr1	36450308	36450572	265	98	4.42674	14.79414	9.87103	138	OSCP1
chr1	37859210	37859478	269	24	2.68632	6.61392	2.45893	207	MTF1,ENSG00000230955
chr1	39142731	39142924	194	69	3.40062	11.63439	6.95575	122	MACF1
chr1	39941549	39941754	206	149	6.04432	20.30506	14.99253	96	NA
chr1	42463026	42463330	305	258	4.58072	31.81516	25.88447	155	PPCS,CCDC30
chr1	43147734	43148040	307	267	5.82963	32.6817	26.7038	139	FAM183A
chr1	44807275	44807523	249	141	4.05872	19.43479	14.17648	143	DYNLT4,ENSG00000290041,BTBD19
chr1	53940160	53940472	313	56	4.89686	10.20991	5.65609	82	HSPB11
chr1	54028040	54028286	247	75	4.4104	12.25609	7.52793	138	TMEM59

chr1	59057304	59057603	300	59	3.52879	10.50498	5.92566	138	LINC01358,ENSG00000241042,ENSG00000235215
chr1	65068158	65068418	261	53	3.99715	9.91648	5.39115	139	JAK1,ENSG00000288804
chr1	67580023	67580268	246	108	4.94248	15.83136	10.82769	143	NA
chr1	74198024	74198254	231	179	4.36072	23.46631	17.95593	133	LRRIQ3,FPGT-TNNI3K,FPGT
chr1	74652893	74653065	173	46	2.96319	9.1213	4.67548	50	ERICH3
chr1	74673715	74673883	169	35	2.98252	7.87747	3.56283	111	ERICH3
chr1	75074860	75075044	185	72	6.21873	11.98628	7.28641	51	NA
chr1	85062009	85062484	476	446	9.80772	51.74451	44.6815	293	DNAI3
chr1	87154361	87154563	203	79	4.16714	12.7073	7.94803	78	ENSG00000267561,LINC01140
chr1	91830312	91830514	203	104	4.79651	15.37246	10.40467	132	TGFBR3,RN7SL653P
chr1	92784785	92784990	206	61	3.19113	10.71702	6.12358	124	EVI5
chr1	100413144	100413339	196	73	3.29224	12.03666	7.33139	80	CDC14A
chr1	111346466	111346773	308	129	3.92343	18.16022	12.98935	129	PIFO
chr1	113930599	113930880	282	76	3.83596	12.38638	7.64784	38	HIPK1-AS1,HIPK1
chr1	113966154	113966322	169	68	4.6039	11.51716	6.85147	74	HIPK1
chr1	115099336	115099752	417	267	5.82963	32.6817	26.7038	191	LINC01765
chr1	115677252	115677432	181	30	3.58081	7.28596	3.03705	55	VANGL1
chr1	116002844	116003053	210	88	4.79375	13.67575	8.83181	149	SLC22A15
chr1	118185197	118185455	259	140	4.83949	19.2665	14.02054	132	SPAG17,ENSG00000287980
chr1	154206245	154206543	299	133	4.56699	18.50021	13.30939	141	CFAP141
chr1	155194850	155195048	199	136	3.91182	18.91337	13.69425	78	MUC1,THBS3-AS1,MIR92B
chr1	156003425	156003616	192	75	3.95272	12.26488	7.53398	126	ARHGEF2,ENSG00000285677
chr1	159900014	159900426	413	168	5.24278	22.28522	16.84596	142	CFAP45,MIR4259,ENSG00000287040
chr1	161165925	161166241	317	126	3.71849	17.82574	12.67878	113	ENSG00000290115,PPOX
chr1	161367736	161368003	268	200	6.95096	25.64522	20.01699	142	CFAP126,ENSG00000288670
chr1	164638071	164638321	251	208	7.25123	26.52814	20.85386	137	PBX1,RNU6-171P
chr1	173669712	173669901	190	94	4.13373	14.33291	9.44188	125	ENSG00000285777,ANKRD45,ENSG00000289426
chr1	176893643	176893865	223	151	7.54173	20.43329	15.1145	99	ASTN1
chr1	183198455	183198674	220	75	3.95272	12.26488	7.53398	109	LAMC2
chr1	183653182	183653460	279	259	5.74866	31.87829	25.94411	140	RGL1,APOBEC4

chr1	192489791	192490087	297	186	4.93899	24.19118	18.64128	150	ENSG00000285280
chr1	194184592	194184784	193	61	4.43339	10.76953	6.16822	96	ENSG00000227240,EEF1A1P14
chr1	202069726	202069966	241	82	4.94493	13.05854	8.26293	122	NA
chr1	202964415	202964619	205	22	2.86213	6.36615	2.25068	200	CYB5R1
chr1	205563840	205564040	201	129	3.92343	18.16022	12.98935	74	RNU2-19P,MFSD4A
chr1	211788802	211788980	179	89	5.89198	13.76768	8.91668	62	LPGAT1
chr1	211893372	211893624	253	208	6.03888	26.56787	20.89046	93	NA
chr1	214337566	214337848	283	164	6.2918	21.88351	16.47357	132	NA
chr1	214602936	214603104	169	40	2.69338	8.41305	4.03616	41	CENPF
chr1	216855176	216855403	228	43	3.60668	8.81714	4.39715	88	ESRRG
chr1	217631144	217631569	426	150	5.12921	20.38161	15.06464	134	GPATCH2,SPATA17
chr1	221230696	221230918	223	296	8.64095	35.81319	29.6795	102	NA
chr1	222044049	222044285	237	164	6.2918	21.88351	16.47357	115	LINC01705
chr1	228809771	228809995	225	87	3.57103	13.57408	8.7456	105	NA
chr1	229030753	229030984	232	114	6.59901	16.53457	11.47805	130	LINC02815
chr1	234523592	234523823	232	53	3.00596	9.88064	5.36159	157	NA
chr1	244362240	244362427	188	50	3.26213	9.52161	5.02808	92	C1orf100
chr1	244969461	244969743	283	134	4.07952	18.69032	13.48072	139	ENSG00000272195,EFCAB2
chr1	245060800	245060994	195	62	4.89412	10.87969	6.26517	144	EFCAB2
chr10	7834331	7834597	267	93	5.74527	14.24254	9.36621	134	TAF3,ENSG00000233990
chr10	13511919	13512119	201	62	4.74888	10.88828	6.27143	65	BEND7
chr10	14653712	14653952	241	172	6.44161	22.76446	17.29613	110	FAM107B,ENSG00000236495
chr10	14959344	14959534	191	87	3.6778	13.55988	8.73794	80	DCLRE1C,MEIG1
chr10	17216297	17216575	279	49	3.92881	9.45641	4.97039	51	VIM-AS1
chr10	22345412	22345668	257	207	7.04083	26.38529	20.7163	116	ENSG00000272366,SPAG6
chr10	22927891	22928204	314	282	5.99156	34.30632	28.24315	154	ARMC3
chr10	23346050	23346228	179	109	5.24317	16.01515	10.99714	77	C10orf67
chr10	25016072	25016296	225	84	3.23082	13.23102	8.4251	126	ENSG00000285859,ENKUR,THNSL1
chr10	27483122	27483373	252	79	4.16714	12.7073	7.94803	166	NA
chr10	27998755	27998972	218	67	4.34434	11.45519	6.79668	110	ODAD2,ENSG00000233472

chr10	31186700	31186878	179	98	5.78926	14.73872	9.81891	114	LINC02664
chr10	38008060	38008239	180	94	4.28212	14.28609	9.40208	78	ZNF33A
chr10	43408686	43408963	278	277	6.65429	33.82823	27.78572	157	HNRNPF
chr10	43436995	43437471	477	102	3.98616	15.15791	10.20182	132	ZNF487
chr10	48274543	48274777	235	49	4.14102	9.44346	4.96176	103	FRMPD2
chr10	48935193	48935420	228	43	3.60668	8.81714	4.39715	15	WDFY4,ENSG00000241577,LRRC18
chr10	50035510	50035698	189	131	5.93109	18.32917	13.15204	125	FAM21EP
chr10	57199626	57199822	197	94	5.62977	14.36917	9.47543	77	ENSG00000289158
chr10	60387596	60387781	186	64	2.88471	11.11648	6.48421	54	ANK3
chr10	60943374	60943598	225	52	3.51353	9.7355	5.22177	99	RHOBTB1,ENSG00000289989
chr10	63273688	63273895	208	94	4.28212	14.28609	9.40208	83	JMJD1C
chr10	78204114	78204393	280	80	5.14521	12.85084	8.08394	172	ENSG00000282863,LINC00595
chr10	80356590	80356802	213	73	3.29224	12.03666	7.33139	163	DYDC1,DYDC2
chr10	80429835	80430005	171	64	3.31965	11.06255	6.43556	94	PRXL2A
chr10	82661126	82661360	235	112	5.22358	16.3254	11.294	151	NRG3
chr10	93445704	93445875	172	150	4.546	20.33859	15.02267	86	MYOF
chr10	96633152	96633428	277	245	7.39618	30.38652	24.52205	118	PIK3AP1
chr10	97401283	97401536	254	321	6.3964	38.46808	32.17224	116	RRP12,ENSG00000231970
chr10	102699055	102699240	186	108	4.94248	15.83136	10.82769	102	ARL3
chr10	104232192	104232521	330	428	9.28059	49.82674	42.85836	191	CFAP43,GSTO1
chr10	110013790	110013980	191	59	3.52879	10.50498	5.92566	107	ADD3
chr10	110422921	110423125	205	81	4.64395	12.92183	8.14108	71	NA
chr10	116849418	116849623	206	109	4.40158	15.94629	10.93424	137	HSPA12A,ENO4
chr10	118047008	118047176	169	33	3.02468	7.64301	3.34791	33	RAB11FIP2,CASC2
chr10	124565960	124566167	208	205	5.74689	26.24231	20.58504	109	LHPP
chr10	124795728	124796074	347	317	7.46625	38.05654	31.79645	113	ENSG00000258539,EEF1AKMT2
chr10	125096521	125096695	175	67	4.34434	11.45519	6.79668	88	CTBP2
chr10	125896552	125896753	202	42	3.92184	8.6258	4.22664	39	DHX32,FANK1
chr10	132942564	132942757	194	24	2.82303	6.59238	2.43992	153	CFAP46
chr11	236746	236945	200	137	4.60177	18.96897	13.74721	136	SIRT3,PSMD13

chr11	537339	537536	198	68	3.22067	11.48341	6.82011	132	HRAS,LRRC56
chr11	3498569	3498745	177	55	4.55101	10.1125	5.56723	29	ENPP7P15,ENSG00000254757,ENSG00000255367
chr11	8086363	8086599	237	108	4.94248	15.83136	10.82769	117	TUB
chr11	8597277	8597572	296	114	4.82109	16.47051	11.4164	187	STK33
chr11	9568884	9569080	197	34	2.56027	7.76681	3.46548	138	WEE1
chr11	10858140	10858354	215	100	3.6501	14.93691	10.00835	104	ZBED5,ZBED5-AS1
chr11	15967194	15967364	171	66	4.29324	11.3132	6.667	117	SOX6
chr11	16981754	16981985	232	175	5.98653	23.04104	17.55829	136	PLEKHA7
chr11	17291745	17291944	200	149	7.6698	20.21567	14.90739	113	NUCB2
chr11	61480901	61481203	303	88	4.0329	13.66761	8.82719	220	ENSG00000256591,PPP1R32
chr11	63567001	63567294	294	172	4.63474	22.70859	17.24999	164	PLAAT2
chr11	66619203	66619492	290	133	3.51188	18.52514	13.33055	134	RNU4-39P,RBM14,RBM14-RBM4
chr11	68532727	68533032	306	46	3.40373	9.13495	4.68577	102	PPP6R3
chr11	70626033	70626210	178	28	3.71778	7.10754	2.88472	94	SHANK2,SHANK2-AS1
chr11	71710275	71710629	355	92	5.54036	14.15946	9.28673	233	ALG1L9P,ENSG00000254972,ENPP7P8
chr11	72812650	72812834	185	82	3.93208	13.01288	8.22195	105	ENSG00000285693,ATG16L2
chr11	73983329	73983543	215	55	2.97639	10.1223	5.5753	77	UCP2,ENSG00000287425
chr11	76065569	76065750	182	73	5.82789	12.05837	7.35016	109	UVRAG
chr11	80024257	80024432	176	65	5.18494	11.15267	6.51675	117	NA
chr11	82830793	82831072	280	95	4.94356	14.44287	9.54021	133	PRCP,EIF2S2P6
chr11	83193632	83193847	216	56	3.11515	10.17479	5.62294	90	ANKRD42-DT,ANKRD42
chr11	93197213	93197381	169	13	2.42636	5.23026	1.33031	85	SLC36A4
chr11	96035418	96035634	217	55	4.68133	10.08274	5.54538	159	MAML2
chr11	101914821	101915210	390	122	3.60145	17.39633	12.27889	192	ANGPTL5,CEP126
chr11	102414698	102414899	202	76	4.67235	12.34522	7.61132	51	TMEM123
chr11	102469448	102469679	232	79	4.46562	12.72899	7.96819	92	TMEM123,ENSG00000255482
chr11	111893649	111893855	207	102	5.47834	15.15422	10.20037	107	RPL37AP8
chr11	111927253	111927441	189	53	3.00596	9.88064	5.36159	59	CRYAB,DIXDC1,RNA5SP351
chr11	117098490	117098662	173	115	5.32954	16.63567	11.57191	118	SIK3,ENSG00000224077
chr11	117327732	117328001	270	126	4.63784	17.80967	12.66607	114	CEP164,ENSG00000250699

chr11	118173615	118173783	169	48	4.35314	9.35489	4.88552	66	SCN2B
chr11	119195850	119196183	334	161	5.14195	21.51784	16.12467	167	CCDC153
chr11	119353019	119353233	215	184	6.27829	24.00343	18.46539	117	NA
chr11	119381688	119381869	182	136	3.91182	18.91337	13.69425	101	USP2,USP2-AS1
chr11	126091976	126092156	181	112	5.62699	16.31211	11.2813	97	NA
chr11	128422667	128422861	195	81	4.06252	12.92731	8.14399	52	NA
chr11	129234434	129234915	482	94	4.67532	14.32593	9.43821	342	ARHGAP32
chr12	694083	694267	185	67	4.70875	11.45267	6.79668	70	NA
chr12	3571352	3571539	188	122	5.20964	17.38262	12.26658	143	PRMT8
chr12	6904793	6905103	311	366	7.10576	43.32287	36.69548	136	LRRC23
chr12	8572772	8572972	201	76	5.08749	12.35811	7.62338	112	ENSG00000255801
chr12	11771490	11771662	173	42	3.74512	8.69985	4.29263	83	ETV6
chr12	11862869	11863142	274	127	4.83111	17.86851	12.71651	136	ETV6
chr12	18966987	18967228	242	94	4.28212	14.28609	9.40208	134	NA
chr12	21628026	21628326	301	154	5.04113	20.75881	15.41184	177	ENSG00000285854
chr12	22588947	22589220	274	114	4.15947	16.47057	11.4164	123	C2CD5-AS1
chr12	25195014	25195276	263	32	2.39215	7.5817	3.2985	138	DNAI7,ETFRF1
chr12	28191764	28192044	281	89	3.91456	13.76323	8.91324	115	CCDC91,ENSG00000247934
chr12	48904011	48904289	279	64	3.31965	11.06255	6.43556	134	RPL32P27,ENSG00000272822,CCDC65
chr12	49201928	49202216	289	201	5.28599	25.80918	20.1692	133	TUBA1C
chr12	55420450	55420928	479	137	5.4768	18.97742	13.75262	330	NA
chr12	56677335	56677550	216	61	4.19454	10.74336	6.14631	115	PTGES3
chr12	56752407	56752583	177	87	4.33898	13.57186	8.74524	104	PRIM1,HSD17B6
chr12	57936002	57936178	177	20	2.16276	6.18864	2.09457	140	ATP23,GIHCG
chr12	64984092	64984326	235	221	5.95584	27.90839	22.16159	119	LINC02389,LINC02231
chr12	75329960	75330186	227	100	4.23455	15.00853	10.0678	115	CAPS2,GLIPR1L1
chr12	85036272	85036445	174	63	3.1491	10.93952	6.32005	78	TSPAN19,LRRIQ1
chr12	89863964	89864164	201	28	2.92386	7.11056	2.88516	138	ENSG00000258216
chr12	94458789	94459024	236	109	5.24317	16.01515	10.99714	148	CEP83,CEP83-DT
chr12	96255269	96255516	248	92	5.54036	14.15946	9.28673	128	ELK3

chr12	96489441	96489760	320	241	6.47807	29.975460000000002	24.13077	135	ENSG00000258272,CFAP54
chr12	96810054	96810328	275	289	8.93355	34.99819	28.90435	132	CFAP54
chr12	100200608	100200845	238	91	3.25292	13.99406	9.12954	141	ENSG00000257489,ACTR6
chr12	111424258	111424426	169	61	5.34704	10.75973	6.16171	24	SH2B3
chr12	113149572	113149842	271	84	3.72619	13.25855	8.44928	128	CFAP73
chr12	113220982	113221168	187	121	3.65753	17.22575	12.12006	134	IQCD,TPCN1
chr12	116257192	116257462	271	76	5.08749	12.35811	7.62338	112	MED13L
chr12	119208663	119208876	214	59	3.52879	10.50498	5.92566	109	HSPB8
chr12	119334529	119334843	315	145	5.61039	19.7905	14.51434	94	CCDC60
chr12	121758568	121758996	429	88	4.79375	13.67575	8.83181	249	TMEM120B
chr12	122182990	122183295	306	63	3.73313	10.97547	6.35144	129	LRRC43
chr12	131956057	131956285	229	39	3.37476	8.36097	3.99153	128	EP400
chr13	23980232	23980409	178	113	4.43619	16.38981	11.35339	103	ENSG00000273167,SPATA13
chr13	27960883	27961128	246	38	3.1255	8.18931	3.83601	50	CDX2
chr13	32334849	32335039	191	31	4.27764	7.43028	3.15888	158	BRCA2
chr13	34750414	34750683	270	111	5.28878	16.14593	11.12206	115	NA
chr13	35617392	35617576	185	43	3.60668	8.81714	4.39715	31	NBEA
chr13	40172301	40172547	247	56	3.73634	10.21547	5.65889	133	LINC00598,LINC00332
chr13	40753492	40753670	179	26	2.79611	6.84579	2.65218	95	MRPS31
chr13	42781448	42781675	228	121	4.94162	17.22235	12.11731	100	FAM216B
chr13	44068930	44069128	199	56	3.11515	10.17479	5.62294	74	NA
chr13	45231768	45232022	255	57	4.94926	10.31802	5.75532	154	GTF2F2
chr13	50015219	50015501	283	199	7.60644	25.54087	19.91882	117	DLEU2,TRIM13,KCNRG
chr13	76884273	76884441	169	48	4.35314	9.35489	4.88552	106	KCTD12,ENSG00000278727
chr13	85224474	85224678	205	120	5.79751	17.15858	12.05994	94	NA
chr13	95060072	95060240	169	42	3.03294	8.6438	4.24131	121	ABCC4
chr13	103655737	103655948	212	69	4.94675	11.6815	6.99874	88	NA
chr13	110134920	110135113	194	74	4.09793	12.19176	7.46991	107	ENSG00000287575
chr13	114260150	114260359	210	129	4.80863	18.12607	12.9573	91	CDC16
chr14	31127845	31128033	189	89	5.89198	13.76768	8.91668	85	AP4S1,HECTD1

chr14	49635158	49635421	264	168	5.24278	22.28522	16.84596	142	DNAAF2
chr14	51401360	51401536	177	43	3.60668	8.81714	4.39715	36	FRMD6-AS2
chr14	55591011	55591198	188	115	5.60158	16.63487	11.57131	78	KTN1
chr14	59429915	59430083	169	39	4.29332	8.33231	3.96771	99	GPR135
chr14	59465320	59465531	212	52	3.51353	9.7355	5.22177	99	GPR135,L3HYPDH
chr14	59679964	59680144	181	55	4.26287	10.03802	5.50484	98	RTN1
chr14	61017376	61017611	236	201	5.92908	25.73507	20.10077	129	SLC38A6
chr14	62117176	62117410	235	133	6.31429	18.49082	13.30363	140	ENSG00000289503,ENSG00000258903,<br>LINC00643
chr14	66007056	66007275	220	69	5.82701	11.66901	6.98777	63	ENSG00000258847
chr14	73644969	73645191	223	87	4.33898	13.57186	8.74524	55	DNAL1
chr14	75258990	75259361	372	210	3.75154	26.77951	21.09317	210	ENSG00000258740,ENSG00000289340,<br>ENSG00000258820
chr14	75277152	75277394	243	233	5.87184	29.20237	23.39009	111	FOS
chr14	75294076	75294334	259	160	4.26165	21.39963	16.01681	150	DPPA5P4,LINC01220
chr14	77086350	77086582	233	76	3.83126	12.36897	7.63095	120	ENSG00000258473
chr14	77287705	77287894	190	36	3.75133	7.94646	3.61898	98	POMT2
chr14	77377170	77377445	276	57	3.29291	10.29271	5.73246	137	TMED8,SAMD15
chr14	78752965	78753181	217	92	4.40527	14.0992	9.23	74	NRXN3,ENSG00000258478
chr14	82404090	82404289	200	68	5.76976	11.56225	6.89055	95	NA
chr14	88824375	88824551	177	79	4.01251	12.75157	7.99044	75	ENSG00000274492,TTC8
chr14	89349385	89349635	251	160	4.9398	21.40232	16.01681	115	FOXN3,ENSG00000258752
chr14	89350117	89350333	217	233	6.38476	29.1755	23.37119	119	FOXN3,ENSG00000258752,ENSG00000258699
chr14	93926297	93926552	256	92	5.87507	14.13193	9.26111	122	FAM181A-AS1,FAM181A
chr14	95664613	95664790	178	85	5.34249	13.31768	8.50614	106	TCL6,ENSG00000259084
chr14	97975280	97975517	238	98	6.16121	14.74507	9.82522	138	LINC01550
chr14	101851834	101852030	197	65	5.18494	11.15267	6.51675	142	PPP2R5C
chr14	102304968	102305324	357	219	4.43802	27.65781	21.92409	177	MOK
chr14	105294390	105294578	189	115	5.07606	16.60141	11.53973	106	BRF1
chr14	105474179	105474444	266	108	5.1815	15.83721	10.83301	147	ENSG00000257270,CRIP2
chr14	105481924	105482158	235	139	4.50609	19.20069	13.96615	133	CRIP1

chr15	33804215	33804384	170	73	5.42062	12.00634	7.30418	94	RYR3
chr15	36080899	36081127	229	132	7.07037	18.45565	13.27337	115	ENSG00000259639
chr15	37984477	37984646	170	45	3.49529	8.9649	4.52995	37	LINC02345
chr15	42913619	42913826	208	97	3.80545	14.63979	9.72598	136	TTBK2
chr15	43185318	43185492	175	71	3.63495	11.87997	7.18327	90	ENSG00000285080,ENSG00000285117, ENSG00000261687,CCNDBP1
chr15	43510921	43511129	209	83	3.43538	13.1705	8.36821	87	TP53BP1,MAP1A
chr15	49035929	49036104	176	89	3.91456	13.76323	8.91324	69	SECISBP2L
chr15	51622933	51623122	190	40	3.07573	8.413	4.03616	72	DMXL2
chr15	56464955	56465237	283	105	3.29143	15.51912	10.54492	154	MNS1
chr15	56640259	56640444	186	48	4.09236	9.32322	4.85747	114	ZNF280D
chr15	70252390	70252621	232	77	5.14462	12.49151	7.74634	113	NA
chr15	73596228	73596554	327	114	4.82109	16.47051	11.4164	159	NPTN
chr15	74318460	74318775	316	340	8.24615	40.47173	34.01321	163	CCDC33,ENSG00000277749
chr15	77351064	77351232	169	105	5.45648	15.47848	10.50635	82	PEAK1
chr15	78267282	78267474	193	203	7.59898	26.02071	20.37582	88	DNAJA4-DT,DNAJA4
chr15	84831680	84831886	207	92	5.87507	14.13193	9.26111	111	ALPK3
chr15	86540903	86541191	289	74	4.27458	12.15924	7.44426	123	AGBL1
chr15	90034436	90034668	233	120	4.53702	17.09502	12.0074	93	ZNF710
chr16	610889	611076	188	92	4.22941	14.09838	9.22938	117	RAB40C
chr16	1331858	1332040	183	87	4.33898	13.57186	8.74524	85	BAIAP3
chr16	1680191	1680439	249	146	5.09698	19.90001	14.62105	116	ENSG00000261732,JPT2
chr16	4414352	4414551	200	59	3.52879	10.50498	5.92566	88	CORO7-PAM16,CORO7
chr16	4734707	4734942	236	66	3.54201	11.27452	6.62927	150	ANKS3,ENSG00000266994,DNAAF8
chr16	10323070	10323246	177	48	4.09236	9.32322	4.85747	52	ATF7IP2
chr16	12088174	12088358	185	113	4.43619	16.38981	11.35339	121	SNX29,ENSG00000175604
chr16	22201824	22202104	281	162	5.3801	21.69285	16.29047	141	EEF2K
chr16	23453175	23453366	192	107	4.61197	15.75579	10.75851	79	COG7,ENSG00000260136
chr16	27512108	27512292	185	42	2.88722	8.61052	4.21833	124	GTF3C1
chr16	27625421	27625636	216	266	6.472	32.61878	26.64407	88	KATNIP

chr16	30948328	30948526	199	135	5.07317	18.73752	13.52609	78	FBXL19,ENSG00000261487,ORAI3
chr16	46745585	46745843	259	95	4.94356	14.44287	9.54021	179	MYLK3
chr16	51755177	51755368	192	67	4.96223	11.37253	6.7231	99	LINC01571
chr16	53044273	53044664	392	364	7.3631	43.06275	36.44422	219	ENSG00000277639
chr16	54204907	54205092	186	101	4.8089	15.07277	10.12219	121	NA
chr16	55830165	55830333	169	42	3.74512	8.69985	4.29263	46	CES1
chr16	57694778	57694966	189	100	4.23455	15.00853	10.0678	65	ENSG00000260467,DRC7
chr16	57735628	57735826	199	75	3.55194	12.27868	7.54757	121	ENSG00000288753,KATNB1
chr16	58495175	58495426	252	131	7.48586	18.3726	13.19309	158	NDRG4,RNU6-103P
chr16	58551478	58551796	319	392	10.74244	46.09171	39.28614	153	CNOT1,SNORA46
chr16	66927183	66927362	180	142	5.3032	19.53907	14.27463	78	RRAD
chr16	67396363	67396653	291	328	7.25923	39.18304	32.83024	136	TPPP3,ZDHHC1
chr16	67857947	67858170	224	152	4.53415	20.60415	15.27889	76	NUTF2,ENSG00000262141
chr16	68269411	68269590	180	107	5.57407	15.78466	10.78731	65	SLC7A6
chr16	68520763	68520969	207	31	2.75288	7.3613	3.10142	92	NA
chr16	70700737	70700953	217	135	5.18267	18.75686	13.5427	147	VAC14
chr16	71230500	71231002	503	539	13.11323	61.58569	53.90908	219	HYDIN
chr16	75501847	75502084	238	113	4.28737	16.41427	11.37031	113	ENSG00000273971,TMEM231P1
chr16	80540949	80541203	255	58	3.07753	10.40519	5.83306	62	DYNLRB2-AS1,DYNLRB2
chr16	82434945	82435134	190	55	4.55101	10.1125	5.56723	102	ENSG00000259873
chr16	85112375	85112586	212	87	4.8867	13.59372	8.76162	87	CIBAR2
chr16	89657767	89657994	228	133	4.73866	18.53356	13.33676	98	CHMP1A,SPATA33
chr16	89827698	89827912	215	98	4.93338	14.76088	9.83972	130	SPIRE2
chr17	1087831	1087999	169	56	3.11515	10.17479	5.62294	114	ABR
chr17	5508944	5509215	272	106	6.36333	15.59644	10.61729	138	NLRP1
chr17	6831607	6831881	275	108	3.96739	15.90642	10.89522	178	TEKT1
chr17	7354368	7354538	171	42	2.68219	8.67861	4.27506	133	KCTD11,ENSG00000263171,TMEM95
chr17	7471513	7471686	174	70	3.73044	11.73616	7.0489	98	ZBTB4
chr17	7835345	7835571	227	36	2.62926	7.94886	3.62053	116	KDM6B,ENSG00000288748
chr17	8460121	8460472	352	100	4.58003	14.9989	10.06206	189	NDEL1

chr17	9576518	9576857	340	107	4.19417	15.73717	10.74019	162	STX8,CFAP52
chr17	11598226	11598590	365	392	5.8249	46.08544	39.28434	162	DNAH9
chr17	12069319	12069551	233	81	4.64395	12.92183	8.14108	79	MAP2K4
chr17	12415139	12415439	301	81	4.21845	12.87579	8.10132	151	NA
chr17	16380577	16380761	185	106	4.02139	15.58833	10.61077	126	UBB
chr17	17972796	17973030	235	118	3.2156	16.94045	11.86027	108	TOM1L2,DRC3
chr17	18179333	18179501	169	55	3.87384	10.11109	5.56723	72	MYO15A,ENSG00000266677,ALKBH5
chr17	19254775	19254978	204	46	3.40373	9.13495	4.68577	134	EPN2
chr17	21295347	21295625	279	278	7.26274	33.84411	27.80097	147	MAP2K3
chr17	30961067	30961435	369	68	3.9774	11.52013	6.85155	230	ENSG00000230113
chr17	31273648	31273821	174	61	4.43339	10.76953	6.16822	140	NF1,OMG
chr17	40322919	40323125	207	107	4.70056	15.72927	10.73674	113	RARA,ENSG00000287644
chr17	41416593	41416875	283	340	7.82375	40.46367	34.00596	161	ENSG00000234859,KRT41P
chr17	47831570	47831814	245	165	4.69609	22.01701	16.59904	93	MRPL10,LRRC46
chr17	57537922	57538149	228	48	4.09236	9.32322	4.85747	126	MSI2
chr17	57912066	57912328	263	233	6.36828	29.11236	23.30912	111	CUEDC1,ENSG00000264914
chr17	57935578	57935758	181	38	3.12934	8.20262	3.84552	115	CUEDC1
chr17	62800756	62800998	243	183	5.44442	23.84446	18.31053	114	MARCHF10
chr17	65564838	65565017	180	48	4.09236	9.32322	4.85747	134	AXIN2,ENSG00000266076
chr17	68512186	68512488	303	120	4.24612	17.14026	12.043	163	ENSG00000267009,PRKAR1A
chr17	72407576	72407761	186	69	3.84292	11.61416	6.93771	94	LINC00511
chr17	73455480	73455732	253	57	4.94926	10.31802	5.75532	147	SDK2
chr17	74274076	74274345	270	261	7.48051	32.14526	26.19381	133	DNAI2
chr17	75011025	75011230	206	102	5.09336	15.22279	10.26303	129	MRPL58
chr17	76141230	76141459	230	182	4.03465	23.80779	18.27817	121	FOXJ1,RNF157-AS1
chr17	78759377	78759578	202	52	3.51353	9.7355	5.22177	73	CYTH1
chr17	80036495	80036802	308	114	4.15947	16.47057	11.4164	143	TBC1D16,CCDC40
chr17	81044641	81044841	201	178	6.09286	23.39534	17.89669	128	BAIAP2
chr17	82636652	82636941	290	134	4.07952	18.69032	13.48072	167	FOXK2,WDR45B
chr18	9838516	9838711	196	62	3.01403	10.879	6.26507	74	RAB31

chr18	9946188	9946362	175	42	3.92184	8.6258	4.22664	150	VAPA
chr18	13381956	13382203	248	80	3.31462	12.80328	8.04025	140	LDLRAD4
chr18	21167723	21168033	311	187	7.16163	24.35243	18.79543	151	NA
chr18	35377266	35377491	226	37	2.94629	8.11762	3.77127	119	ZNF396
chr18	43831023	43831280	258	187	7.71693	24.31714	18.76318	129	NA
chr18	47795487	47795737	251	125	6.33184	17.67238	12.53906	159	NA
chr18	50266411	50266658	248	131	3.55686	18.33877	13.16155	140	CFAP53
chr18	50307695	50307953	259	293	6.32586	35.494	29.37332	150	ENSG00000267695
chr18	74147721	74147900	180	45	2.67204	8.94486	4.51604	114	FBXO15,TIMM21
chr19	1860128	1860347	220	83	5.42183	13.12778	8.32842	116	KLF16,ENSG00000267141
chr19	2785128	2785396	269	58	3.07753	10.40519	5.83306	211	SGTA,THOP1
chr19	3107435	3107627	193	33	3.02468	7.64301	3.34791	147	GNA11
chr19	4640761	4641028	268	148	5.1605	20.13573	14.83334	135	TNFAIP8L1
chr19	4759842	4760017	176	107	4.70056	15.72927	10.73674	91	NA
chr19	5680761	5680943	183	38	2.62123	8.21786	3.85974	154	RPL36,MICOS13,HSD11B1L
chr19	6066599	6066952	354	241	6.47807	29.975460000000002	24.13077	220	RFX2,ENSG00000267262
chr19	6110396	6110651	256	198	4.66752	25.5096	19.89247	151	RFX2,ENSG00000267299
chr19	11815307	11815544	238	115	3.59657	16.63284	11.56964	131	ZNF440
chr19	12140261	12140475	215	92	3.64701	14.17326	9.29826	138	ZNF20,ZNF625-ZNF20
chr19	12703087	12703271	185	88	4.79375	13.67575	8.83181	39	TNPO2
chr19	13846639	13846811	173	71	3.34309	11.83129	7.13864	118	MIR23AHG
chr19	14006070	14006486	417	394	7.1251	46.29669	39.48569	282	RFX1,ENSG00000275091
chr19	16324416	16324594	179	75	4.4104	12.25609	7.52793	57	KLF2-DT,KLF2
chr19	16533337	16533505	169	97	5.28596	14.65806	9.74288	77	ENSG00000141979,CHERP
chr19	18117722	18117903	182	106	4.88325	15.64616	10.66595	115	MAST3
chr19	18281635	18281902	268	361	6.80123	42.76589	36.17828	146	JUND,MIR3188
chr19	18416828	18417083	256	153	5.77964	20.7277	15.3865	130	SSBP4
chr19	18741869	18742077	209	83	3.82136	13.12509	8.32598	96	CRTC1
chr19	35454666	35454861	196	119	7.50903	16.98089	11.90014	77	NA
chr19	36797326	36797515	190	97	3.80545	14.63979	9.72598	129	ZNF790-AS1

chr19	38664057	38664231	175	54	3.90741	9.93931	5.41306	119	ACTN4
chr19	41298320	41298505	186	108	4.94248	15.83136	10.82769	100	HNRNPUL1
chr19	45450739	45451003	265	222	5.43408	28.0066	22.25268	130	ERCC1
chr19	45792829	45793075	247	44	2.75751	8.88719	4.46062	202	DMWD
chr19	46416515	46416781	267	50	3.61582	9.58321	5.08496	106	CCDC8
chr19	48320636	48320926	291	196	6.2594	25.23654	19.63323	148	ODAD1,EMP3
chr19	49813642	49813837	196	31	2.44091	7.44672	3.17426	21	FUZ,MED25
chr19	49817401	49817607	207	107	3.65284	15.7002	10.71118	90	FUZ,MED25
chr19	49817906	49818085	180	48	2.82164	9.37105	4.89584	55	FUZ,MED25
chr19	50051030	50051264	235	122	3.93638	17.40788	12.28917	78	ZNF473,ENSG00000269091,ENSG00000204666
chr19	50510657	50511083	427	90	4.53724	13.89681	9.03675	197	JOSD2
chr19	51108521	51108809	289	87	3.33467	13.56919	8.74524	128	CTU1
chr19	53867428	53867713	286	159	4.76606	21.33487	15.95826	161	MYADM-AS1,MYADM
chr19	53991323	53991516	194	123	7.50069	17.44189	12.32249	102	CACNG6
chr19	54180047	54180567	521	188	5.31302	24.43501	18.8708	162	MBOAT7
chr19	55166626	55166884	259	231	5.62747	28.91024	23.11943	137	DNAAF3,DNAAF3-AS1,ENSG00000239137
chr19	55279779	55280082	304	231	5.63261	28.93396	23.13992	160	HSPBP1,BRSK1
chr19	58033780	58034015	236	87	5.97844	13.54546	8.72363	119	ZSCAN1
chr19	58098284	58098610	327	97	3.46292	14.70334	9.78437	92	ZSCAN18
chr19	58362725	58363044	320	431	5.71894	50.14591	43.15745	161	ENSG00000268230,ZNF497,ZNF497-AS1,RNA5SP473
chr19	58401311	58401488	178	58	2.84631	10.37163	5.80327	66	ZNF584
chr19	58476005	58476173	169	79	3.16986	12.6912	7.933	101	ZNF446,ENSG00000269106
chr19	58573526	58573775	250	145	3.8338	19.80335	14.52618	124	MZF1-AS1,MZF1,CENPBD2P,ENSG00000286104
chr2	11132857	11133186	330	100	4.23455	15.00853	10.0678	214	ENSG00000145063,C2orf50
chr2	24984944	24985158	215	29	3.75953	7.19911	2.9584	96	DNAJC27-AS1
chr2	26401769	26402073	305	167	5.03301	22.14835	16.71877	151	DRC1
chr2	26970313	26970576	264	199	4.93866	25.58577	19.96088	130	ENSG00000230286,MAPRE3
chr2	28621462	28621744	283	48	2.82164	9.37105	4.89584	162	PLB1
chr2	28982075	28982252	178	80	4.40816	12.86882	8.09814	77	TOGARAM2
chr2	28993631	28993871	241	31	2.64811	7.41241	3.14807	109	TOGARAM2

chr2	38620323	38620555	233	82	3.27054	13.01425	8.22237	147	NA
chr2	47230522	47230797	276	130	5.34322	18.17471	13.00103	149	EPCAM-DT,ENSG00000226087
chr2	51857604	51857799	196	62	4.74888	10.88828	6.27143	122	ENSG00000231918
chr2	54545889	54546066	178	87	4.33898	13.57186	8.74524	56	SPTBN1,ENSG00000234943
chr2	55616494	55616779	286	94	4.67532	14.32593	9.43821	137	PPP4R3B,PPP4R3B-DT
chr2	61065509	61065957	449	81	3.49505	12.9839	8.1968	145	SANBR
chr2	61853999	61854223	225	25	2.50288	6.7766	2.58989	77	FAM161A,RPL31P30,ENSG00000277498
chr2	62450761	62450937	177	82	4.94493	13.05854	8.26293	81	ENSG00000228541
chr2	63876033	63876213	181	55	3.87384	10.11109	5.56723	114	UGP2
chr2	70367859	70368062	204	80	4.40816	12.86882	8.09814	79	NA
chr2	71372092	71372260	169	68	5.76976	11.56225	6.89055	137	ZNF638,ENSG00000281195
chr2	73307825	73308024	200	69	4.94675	11.6815	6.99874	96	ENSG00000287435
chr2	74067288	74067470	183	26	2.67191	6.87897	2.67732	85	TET3
chr2	74421569	74421796	228	165	4.69609	22.01701	16.59904	134	ENSG00000159239,C2orf81,HMGA1P8,WDR54
chr2	84516331	84516619	289	244	5.58673	30.28967	24.43038	133	DNAH6
chr2	94871316	94871601	286	261	7.48051	32.14526	26.19381	117	ENSG00000259848,TEKT4
chr2	95945759	95945927	169	50	4.71358	9.50326	5.0128	105	ANKRD36C
chr2	96855019	96855223	205	63	3.1491	10.93952	6.32005	110	ANKRD23,ANKRD39
chr2	98060523	98060700	178	140	5.69083	19.2518	14.00993	103	NA
chr2	98869376	98869604	229	120	3.45069	17.19117	12.09181	129	CRACDL
chr2	101696701	101696895	195	57	3.62333	10.34913	5.78223	62	MAP4K4
chr2	112636771	112636992	222	71	5.36323	11.88625	7.18881	122	NA
chr2	119355923	119356231	309	283	8.73188	34.42583	28.35494	168	C2orf76
chr2	120503826	120504004	179	104	4.92956	15.44379	10.47353	132	NA
chr2	120959485	120959685	201	114	4.82109	16.47051	11.4164	108	GLI2
chr2	125573506	125573715	210	65	5.55037	11.14895	6.51456	145	NA
chr2	127277006	127277234	229	61	4.43339	10.76953	6.16822	111	ERCC3
chr2	143356156	143356324	169	75	3.95272	12.26488	7.53398	52	ARHGAP15,ENSG00000228655
chr2	145495738	145495930	193	49	3.74026	9.45666	4.97039	87	NA
chr2	156331889	156332138	250	131	5.16923	18.34677	13.16814	130	NR4A2

chr2	161362814	161363048	235	82	6.1054	12.98774	8.20017	89	PSMD14
chr2	166125592	166125778	187	48	3.89493	9.36515	4.89178	111	SCN1A-AS1,SCN1A
chr2	169694285	169694539	255	168	4.06615	22.3138	16.87128	126	CFAP210,PHOSPHO2,KLHL23
chr2	177242240	177242471	232	53	3.43218	9.92229	5.39682	94	NFE2L2
chr2	181656963	181657159	197	67	4.34434	11.45519	6.79668	137	CERKL
chr2	190469940	190470191	252	147	4.22266	20.00266	14.70824	100	MFSD6
chr2	196068490	196068959	470	116	3.85251	16.71662	11.64943	347	DNAH7,ENSG00000227878
chr2	201357341	201357539	199	94	4.13373	14.33291	9.44188	76	CASP8,FLACC1
chr2	202073047	202073374	328	88	4.0329	13.66761	8.82719	183	KIAA2012
chr2	202099495	202099674	180	79	4.94817	12.72054	7.96058	100	KIAA2012,KIAA2012-AS1
chr2	212640556	212640748	193	41	3.87682	8.515	4.13196	46	ENSG00000273118
chr2	213284170	213284496	327	152	4.4068	20.62387	15.29041	166	SPAG16-DT,SPAG16
chr2	215529417	215529595	179	89	5.11545	13.85127	8.99668	78	ENSG00000237525
chr2	218356760	218357041	282	134	4.9409	18.61486	13.40676	120	CATIP-AS2,CATIP
chr2	218569911	218570162	252	70	3.73044	11.73616	7.0489	146	USP37,CNOT9
chr2	219041337	219041696	360	261	6.45265	32.09431	26.14712	197	CFAP65
chr2	219266229	219266555	327	203	7.41653	26.02086	20.37582	131	TUBA4A,TUBA4B
chr2	219597946	219598259	314	153	3.76921	20.70956	15.36938	166	STK11IP
chr2	230640460	230640658	199	57	5.27285	10.26044	5.70268	105	NA
chr2	230778578	230779017	440	131	5.16923	18.34677	13.16814	285	CAB39
chr2	237213699	237213875	177	63	5.89142	11.01782	6.3933	25	NA
chr2	237674852	237675182	331	61	3.19113	10.71702	6.12358	130	LRRFIP1
chr2	240557839	240558027	189	61	4.00742	10.77997	6.17484	157	ANKMY1,ENSG00000287049,DUSP28
chr2	240906174	240906407	234	60	2.92602	10.61472	6.031	71	CROCC2
chr2	241149467	241149734	268	100	4.39191	14.98188	10.04919	119	PASK,PPP1R7
chr20	290648	290871	224	62	2.90439	10.86461	6.25183	103	C20orf96
chr20	1325102	1325328	227	59	3.23868	10.50097	5.92425	109	SDCBP2,ENSG00000274322,SDCBP2-AS1
chr20	1423942	1424110	169	108	4.84017	15.85188	10.84693	105	NA
chr20	1467706	1467880	175	68	4.141	11.46283	6.80081	67	NSFL1C
chr20	2207191	2207543	353	62	4.12586	10.84147	6.23501	112	ENSG00000287561,ENSG00000226644,

									ENSG00000286022
chr20	2987162	2987341	180	61	5.71211	10.7005	6.10917	69	PTPRA
chr20	20052516	20052708	193	67	3.29308	11.38561	6.73581	27	CRNKL1,CFAP61
chr20	31879717	31879906	190	95	4.00807	14.41265	9.51423	122	TTLL9
chr20	35540046	35540345	300	62	3.37957	10.86481	6.25183	186	ERGIC3
chr20	35769232	35769464	233	135	5.18267	18.75686	13.5427	121	PHF20
chr20	36944370	36944611	242	166	4.43737	22.02507	16.60537	146	SAMHD1
chr20	46090012	46090249	238	121	3.65753	17.22575	12.12006	102	NCOA5
chr20	47295958	47296230	273	140	4.83949	19.2665	14.02054	146	ZMYND8
chr20	47686636	47686841	206	140	4.25484	19.27062	14.02367	113	SULF2
chr20	49420391	49420568	178	81	4.64395	12.92183	8.14108	129	KCNB1,ENSG00000283529
chr20	50730417	50730587	171	68	3.68406	11.56515	6.89335	103	PARD6B
chr20	51531062	51531246	185	78	5.72934	12.64201	7.88748	58	NFATC2
chr20	58150797	58151043	247	127	4.10289	17.94781	12.79095	91	C20orf85
chr21	17512752	17512949	198	83	3.82136	13.12509	8.32598	125	CXADR
chr21	28892410	28892593	184	82	4.94493	13.05854	8.26293	57	HSPD1P7
chr21	32433425	32433692	268	262	5.97923	32.16423	26.21102	126	CFAP298-TCP10L,EVA1C
chr21	32612493	32612802	310	118	3.78814	16.91502	11.83545	250	CFAP298-TCP10L,CFAP298
chr21	42496120	42496435	316	330	6.08349	39.40646	33.04144	154	RSPH1,SLC37A1,RSPH1-DT
chr21	45287885	45288067	183	100	4.39191	14.98188	10.04919	85	POFUT2,LINC00205
chr21	46318208	46318578	371	361	8.4369	42.78077	36.18279	143	C21orf58
chr22	17198738	17199009	272	33	3.02468	7.64301	3.34791	30	ADA2
chr22	20038222	20038421	200	67	4.70875	11.45267	6.79668	113	TANGO2
chr22	21014271	21014469	199	103	3.88642	15.26869	10.30467	101	ENSG00000284060,TUBA3FP,P2RX6, ENSG00000284130
chr22	21045655	21045954	300	347	5.89979	41.25539	34.74314	154	P2RX6P,LRRC74B
chr22	23141963	23142158	196	179	4.58051	23.40797	17.90723	124	RSPH14,RAB36
chr22	23145259	23145551	293	302	6.00237	36.44796	30.28272	149	RSPH14,RAB36
chr22	25159125	25159314	190	29	3.13371	7.19822	2.9584	143	KIAA1671
chr22	30475065	30475316	252	146	4.45319	19.90908	14.62281	131	SEC14L3,ENSG00000181123

chr22	30667760	30667985	226	141	4.05872	19.43479	14.17648	123	SLC35E4,DUSP18,ENSG00000288153
chr22	37844154	37844380	227	92	3.64701	14.17326	9.29826	132	ANKRD54,MIR658,EIF3L
chr22	38656261	38656529	269	74	2.90112	12.11393	7.40468	135	FAM227A,CBY1
chr22	48370025	48370285	261	90	6.38292	13.9362	9.07422	150	NA
chr22	50207593	50207868	276	29	3.75953	7.19911	2.9584	135	SELENOO,ENSG00000272836
chr22	50532194	50532362	169	74	4.27458	12.15924	7.44426	120	TYMP,ODF3B
chr22	50783545	50783732	188	71	3.02057	11.87567	7.1816	96	RPL23AP82,RABL2B
chr3	3110183	3110522	340	172	5.88384	22.69491	17.23881	222	IL5RA
chr3	8138948	8139154	207	81	4.21845	12.87579	8.10132	93	LMCD1-AS1
chr3	11254943	11255145	203	101	4.8089	15.07277	10.12219	109	HRH1
chr3	12721157	12721333	177	92	4.93769	14.07913	9.21065	83	NA
chr3	15378160	15378375	216	84	3.72619	13.25855	8.44928	79	HMGN2P7
chr3	15512288	15512464	177	76	5.42314	12.38347	7.64507	70	COLQ
chr3	19934160	19934359	200	61	4.43339	10.76953	6.16822	98	EFHB
chr3	33802157	33802335	179	48	3.89493	9.36515	4.89178	84	PDCD6IP-DT,PDCD6IP
chr3	39180863	39181046	184	94	3.57853	14.33958	9.44636	89	NA
chr3	41126477	41126671	195	65	4.59473	11.17248	6.53457	133	NA
chr3	41131263	41131738	476	56	3.73634	10.21547	5.65889	313	NA
chr3	41962133	41962564	432	85	3.38226	13.36605	8.5526	337	ULK4
chr3	42220877	42221083	207	128	5.06214	17.98455	12.82302	49	TRAK1,RNU4-78P
chr3	45466841	45467033	193	75	4.88986	12.23178	7.50739	110	LARS2
chr3	47282511	47282714	204	71	3.68313	11.81441	7.1241	115	KIF9,KLHL18
chr3	50112562	50112843	282	128	5.06214	17.98455	12.82302	85	RBM5
chr3	50238864	50239032	169	28	2.92386	7.11056	2.88516	102	GNAI2,ENSG00000213600
chr3	56319374	56319568	195	85	5.2	13.34353	8.53127	74	ERC2
chr3	56557078	56557311	234	120	4.53702	17.09502	12.0074	113	CCDC66
chr3	57544295	57544522	228	152	4.4068	20.62387	15.29041	125	DNAH12,RNU6-483P
chr3	59050092	59050285	194	110	4.8	16.06062	11.03865	85	CFAP20DC,CFAP20DC-DT
chr3	63652460	63652775	316	304	8.21593	36.63785	30.46827	132	SNTN
chr3	66597433	66597609	177	36	3.18414	7.97469	3.64248	125	NA

chr3	70811890	70812156	267	176	7.6501	23.15308	17.66033	134	NA
chr3	75342533	75342738	206	108	4.94248	15.83136	10.82769	90	MYLKP1
chr3	87025739	87025974	236	141	7.75321	19.39868	14.14251	93	NA
chr3	93911162	93911385	224	130	5.34322	18.17471	13.00103	133	PROS1
chr3	93979868	93980165	298	104	3.79897	15.39792	10.42831	156	PROS1,ARL13B
chr3	97764269	97764450	182	75	3.24697	12.24913	7.5227	129	ARL6
chr3	98801131	98801338	208	140	5.84239	19.30247	14.05197	124	ST3GAL6,DCBLD2
chr3	101216840	101217069	230	30	3.58081	7.28596	3.03705	137	NA
chr3	112795666	112795862	197	31	2.75288	7.3613	3.10142	92	NA
chr3	114519474	114519797	324	185	5.70949	24.09338	18.55149	157	ZBTB20,ZBTB20-AS5
chr3	129058450	129058701	252	146	4.45319	19.90908	14.62281	146	GP9
chr3	138113472	138113663	192	70	4.35298	11.78413	7.09453	83	DZIP1L
chr3	140020024	140020244	221	89	5.89198	13.76768	8.91668	100	CLSTN2
chr3	140505324	140505545	222	53	5.07969	9.89332	5.37293	84	CLSTN2
chr3	142117288	142117492	205	146	4.59274	19.90409	14.62115	84	TFDP2
chr3	142866911	142867114	204	151	3.89189	20.4215	15.10306	84	PCOLCE2
chr3	149792775	149793024	250	37	2.70581	8.14834	3.79845	187	ANKUB1
chr3	150632879	150633155	277	249	6.58787	30.84582	24.95986	154	NA
chr3	152258456	152258638	183	83	3.82136	13.12509	8.32598	120	MBNL1,MBNL1-AS1
chr3	158570987	158571199	213	151	3.89189	20.4215	15.10306	107	MLF1-DT,MLF1
chr3	159978452	159978632	181	63	3.73313	10.97547	6.35144	37	IL12A-AS1
chr3	167380199	167380475	277	199	4.93866	25.58577	19.96088	128	ZBBX
chr3	171843089	171843375	287	185	5.70949	24.09338	18.55149	147	TMEM212-AS1,TMEM212
chr3	174949630	174949854	225	121	5.30292	17.30219	12.1929	104	NAALADL2
chr3	180679405	180679640	236	120	4.24612	17.14026	12.043	105	CCDC39,ENSG00000145075,CCDC39-AS1
chr3	183265150	183265427	278	110	3.53561	16.04714	11.02577	111	MCF2L2,B3GNT5
chr3	184338679	184338918	240	113	4.28737	16.41427	11.37031	113	FAM131A
chr3	188090807	188090975	169	52	4.54871	9.79173	5.27591	98	NA
chr3	189955378	189955608	231	221	5.94853	27.87861	22.13197	73	NA
chr3	190860603	190860899	297	81	4.64395	12.92183	8.14108	149	GMNC

chr3	195133543	195133777	235	162	7.05085	21.69084	16.29047	120	XXYLT1
chr4	2418858	2419038	181	90	3.44991	13.93728	9.07422	112	ZFYVE28,CFAP99
chr4	4033206	4033374	169	58	4.41801	10.42235	5.8491	147	NA
chr4	7042857	7043138	282	95	3.18525	14.4385	9.53853	145	ENSG00000245748,CCDC96,TADA2B
chr4	8428429	8428658	230	84	3.23082	13.23102	8.4251	128	ACOX3
chr4	9604064	9604363	300	112	5.47792	16.29862	11.26997	149	ENSG00000287117
chr4	15774202	15774374	173	113	4.61151	16.40499	11.36492	138	CD38
chr4	36206054	36206252	199	107	4.70056	15.72927	10.73674	118	ARAP2
chr4	36281475	36281749	275	165	6.46305	21.94461	16.53082	138	DTHD1
chr4	38161023	38161262	240	175	4.9941	23.04174	17.55852	121	NA
chr4	38516236	38516414	179	87	4.54174	13.59116	8.75955	84	LINC01258,LINC01259
chr4	39539145	39539463	319	126	4.47378	17.79598	12.65595	92	UGDH-AS1,ENSG00000287262
chr4	40312192	40312374	183	67	3.58784	11.45109	6.79668	68	LINC02265
chr4	40377866	40378056	191	35	2.98252	7.87747	3.56283	69	RNU7-74P
chr4	52051219	52051466	248	188	5.31262	24.43329	18.86924	142	SPATA18
chr4	53443123	53443291	169	32	4.06736	7.58369	3.2985	82	FIP1L1,ENSG00000282278
chr4	56530292	56530473	182	97	5.91781	14.62799	9.71652	86	THEGL
chr4	60795096	60795264	169	36	3.75133	7.94646	3.61898	49	LINC02496
chr4	76567122	76567300	179	41	3.29394	8.54787	4.16021	50	SHROOM3
chr4	80058754	80059069	316	223	5.54596	28.14262	22.3838	151	ANTXR2
chr4	84579422	84579740	319	60	3.44854	10.67807	6.08783	153	CDS1
chr4	87935604	87935936	333	94	4.13373	14.33291	9.44188	100	NA
chr4	89029750	89030036	287	120	5.36868	17.12548	12.03407	141	FAM13A
chr4	105181317	105181649	333	122	5.20964	17.38262	12.26658	128	TET2,TET2-AS1
chr4	127632772	127632983	212	120	4.38058	17.10061	12.01049	98	INTU
chr4	128846117	128846423	307	330	6.99029	39.45487	33.07962	156	JADE1
chr4	152679005	152679208	204	55	4.04473	10.04651	5.51034	100	TMEM154
chr4	153152087	153152301	215	60	3.44854	10.67807	6.08783	108	TRIM2,ENSG00000288637
chr4	155376828	155377075	248	99	3.99062	14.89883	9.9721	97	MAP9-AS1,MAP9
chr4	168200808	168200987	180	59	4.57352	10.48178	5.90675	139	NA

chr4	168727948	168728203	256	160	4.9398	21.40232	16.01681	147	PALLD
chr4	184703855	184704028	174	70	5.48024	11.75681	7.06815	83	CENPU
chr4	185397198	185397460	263	337	6.55833	40.17134	33.73528	138	LRP2BP,ANKRD37
chr4	188439919	188440090	172	55	4.04473	10.04651	5.51034	113	LINC01060,ENSG00000289879
chr5	672816	673124	309	273	5.12054	33.36143	27.34613	111	TPPP,ENSG00000271781
chr5	912361	912906	546	310	5.57915	37.3007	31.08395	304	TRIP13,ENSG00000287600
chr5	7850927	7851275	349	145	5.61039	19.7905	14.51434	213	C5orf49,MTRR
chr5	10441766	10442074	309	229	5.42479	28.7259	22.94139	130	ROPN1L-AS1,ROPN1L
chr5	17227760	17227950	191	39	4.00183	8.26991	3.90889	121	BASP1
chr5	17852671	17852904	234	60	3.04404	10.64017	6.0537	132	LINC02223
chr5	33642170	33642401	232	74	5.16899	12.18948	7.46991	76	ADAMTS12
chr5	34839006	34839271	266	79	3.16986	12.6912	7.933	178	TTC23L-AS1,TTC23L
chr5	35617795	35618089	295	267	5.19438	32.71878	26.73873	124	SPEF2
chr5	35938612	35938903	292	241	5.96504	30.02166	24.17332	135	CAPSL,ENSG00000249748
chr5	51432090	51432281	192	57	4.94926	10.31802	5.75532	149	ENSG00000248891
chr5	62167690	62167943	254	88	4.0329	13.66761	8.82719	145	NA
chr5	62603837	62604082	246	127	5.51378	17.93702	12.78189	148	IPO11
chr5	73716539	73716786	248	74	4.49414	12.18149	7.46273	98	ARHGEF28
chr5	74765271	74765468	198	170	5.96323	22.54873	17.0962	105	GFM2,NSA2
chr5	77620533	77620704	172	81	4.21845	12.87579	8.10132	71	WDR41
chr5	78062454	78062835	382	69	4.94675	11.6815	6.99874	240	AP3B1
chr5	80787417	80787676	260	68	5.76976	11.56225	6.89055	124	MSH3
chr5	91463641	91463822	182	62	4.74888	10.88828	6.27143	99	ARRDC3-AS1
chr5	94544362	94544654	293	126	7.22133	17.83655	12.68878	150	KIAA0825
chr5	95392648	95392937	290	205	7.50266	26.25769	20.59754	125	FAM81B
chr5	96714940	96715149	210	103	3.88642	15.26869	10.30467	107	CAST
chr5	96823938	96824120	183	64	4.82552	11.0635	6.43622	99	ENSG00000247121
chr5	98938642	98938820	179	56	3.73634	10.21547	5.65889	98	CHD1-DT
chr5	110726602	110726787	186	140	4.15109	19.34282	14.08999	108	TMEM232
chr5	115169904	115170075	172	77	4.49808	12.50834	7.76015	51	TRIM36

chr5	115571580	115571749	170	42	4.15527	8.61621	4.22012	42	NA
chr5	122129339	122129640	302	167	5.03301	22.14835	16.71877	162	ENSG00000247311,ZNF474,ENSG00000250803
chr5	122323320	122323508	189	71	5.36323	11.88625	7.18881	66	SNCAIP,ENSG00000249621,ENSG00000289054
chr5	123700647	123700840	194	87	4.17232	13.60114	8.7661	102	NA
chr5	126484300	126484468	169	33	3.71113	7.59606	3.30863	14	GRAMD2B,ENSG00000250602
chr5	134437009	134437195	187	95	3.18525	14.4385	9.53853	112	LINC02999,ENSG00000250994
chr5	137604882	137605055	174	75	3.95272	12.26488	7.53398	27	NA
chr5	138350255	138350558	304	106	4.75715	15.58275	10.60529	167	ENSG00000249971,KDM3B
chr5	139689752	139689969	218	125	5.18542	17.67109	12.53861	97	ENSG00000249526
chr5	142360315	142360554	240	149	6.04432	20.30506	14.99253	113	SPRY4-AS1
chr5	143682248	143682488	241	50	3.61582	9.58321	5.08496	152	ENSG00000249881
chr5	156402698	156402962	265	198	4.66752	25.5096	19.89247	137	SGCD
chr5	172765349	172765526	178	68	3.328	11.54135	6.87181	64	ENSG00000213386,ENSG00000253736
chr5	175385765	175385954	190	36	2.62926	7.94886	3.62053	87	NA
chr5	176547653	176548021	369	97	5.43317	14.66044	9.74482	278	CDHR2,RN7SL684P
chr5	178892714	178892914	201	48	5.36507	9.37351	4.89741	145	ZFP2
chr6	3796425	3796598	174	50	3.61582	9.58321	5.08496	65	NA
chr6	3887272	3887467	196	77	5.01429	12.53559	7.78609	94	ENSG00000233068
chr6	3909272	3909453	182	76	3.83126	12.36897	7.63095	143	ENSG00000260604
chr6	9999208	9999392	185	57	4.94926	10.31802	5.75532	36	OFCC1
chr6	10838413	10838615	203	73	3.29224	12.03666	7.33139	116	TMEM14B,ENSG00000272162,MAK
chr6	11318465	11318678	214	33	2.49778	7.60003	3.31217	69	NEDD9
chr6	18053271	18053441	171	46	2.74315	9.14886	4.69898	48	NA
chr6	19804665	19804852	188	76	3.41907	12.40291	7.66165	112	LNC-LBCS
chr6	22236115	22236322	208	23	4.19843	6.45599	2.3254	67	CASC15
chr6	24330219	24330393	175	48	3.32715	9.32179	4.85747	84	DCDC2
chr6	24360001	24360245	245	90	4.24718	13.86368	9.00851	101	DCDC2
chr6	24825289	24825552	264	70	3.73044	11.73616	7.0489	131	RIPOR2
chr6	28896326	28896517	192	37	2.94629	8.11762	3.77127	124	HCG14
chr6	37102540	37102841	302	328	7.26814	39.22051	32.86357	146	NA

chr6	37347649	37347943	295	63	3.73313	10.97547	6.35144	155	NA
chr6	39725282	39725532	251	21	2.27449	6.22165	2.12212	84	KIF6
chr6	44342517	44342747	231	61	4.19454	10.74336	6.14631	84	ENSG00000272442,SPATS1
chr6	44800137	44800342	206	73	3.29224	12.03666	7.33139	103	ENSG00000286417
chr6	45730310	45730516	207	36	3.18414	7.97469	3.64248	94	NA
chr6	47680065	47680359	295	145	5.61039	19.7905	14.51434	145	ADGRF2
chr6	52420185	52420468	284	225	4.58894	28.31176	22.54602	151	EFHC1
chr6	53578474	53578697	224	87	5.97844	13.54546	8.72363	141	GCLC,ENSG00000231683
chr6	53719083	53719251	169	31	2.75288	7.3613	3.10142	43	NA
chr6	79270004	79270285	282	158	7.04421	21.20427	15.83719	127	NA
chr6	84052546	84052760	215	55	3.35534	10.09402	5.55471	188	MRAP2
chr6	107419844	107420052	209	74	5.16899	12.18948	7.46991	96	PDSS2
chr6	108848507	108848726	220	114	4.04835	16.55282	11.49466	163	ARMC2
chr6	109440499	109440769	271	120	5.00752	17.0917	12.00691	134	PPIL6,SMPD2
chr6	113713783	113713975	193	67	4.34434	11.45519	6.79668	125	NA
chr6	113824576	113824784	209	102	5.20197	15.16669	10.20978	85	ENSG00000231912
chr6	116616350	116616562	213	114	3.65531	16.46313	11.41543	136	RSPH4A
chr6	122389728	122390037	310	134	4.9409	18.61486	13.40676	179	NA
chr6	122671304	122671540	237	48	3.89493	9.36515	4.89178	114	PKIB
chr6	126110466	126110736	271	151	6.92545	20.44782	15.12792	130	TRMT11
chr6	127320288	127320535	248	62	3.85687	10.86124	6.25117	101	ECHDC1,RNA5SP217
chr6	129661734	129662151	418	613	14.85068	70.88221	61.39269	228	ARHGAP18
chr6	131366160	131366415	256	146	4.45319	19.90908	14.62281	159	ENSG00000290067
chr6	131717767	131717958	192	45	3.95406	8.97747	4.54111	62	ENPP3
chr6	132067403	132067637	235	39	3.37476	8.36097	3.99153	134	CCN2-AS1
chr6	132076664	132076855	192	128	5.96802	18.01752	12.85348	60	CCN2-AS1
chr6	136288857	136289091	235	104	3.79897	15.39792	10.42831	131	BCLAF1,ENSG00000289312
chr6	137415089	137415380	292	151	5.94908	20.43759	15.11862	149	NA
chr6	137717469	137717789	321	249	5.47051	30.87075	24.98133	163	LINC03004
chr6	144098971	144099245	275	74	4.09793	12.19176	7.46991	116	SF3B5

chr6	146598773	146599129	357	231	7.40214	28.92895	23.13788	201	ADGB-DT,ADGB
chr6	150177876	150178143	268	155	5.2703	20.90985	15.55546	152	PPP1R14C
chr6	151493874	151494147	274	231	7.25581	28.96362	23.16783	136	CCDC170
chr6	158644863	158645181	319	272	6.40883	33.23581	27.2273	155	DYNLT1,SYTL3
chr6	161074833	161075031	199	51	3.91768	9.69744	5.19088	102	MAP3K4
chr6	162727843	162728117	275	273	5.12054	33.36143	27.34613	110	PRKN,PACRG
chr6	165266780	165266948	169	87	4.33898	13.57186	8.74524	107	NA
chr6	165775108	165775492	385	91	3.72448	14.01991	9.15221	152	PDE10A,ENSG00000236627,ENSG00000223942
chr6	166309138	166309626	489	337	9.13809	40.13509	33.70551	272	PRR18,ENSG00000287189
chr6	167893517	167893713	197	43	3.60668	8.81714	4.39715	97	AFDN
chr6	170295965	170296163	199	107	4.70056	15.72927	10.73674	104	DLL1,FAM120B
chr7	521883	522091	209	122	5.20964	17.38262	12.26658	94	PDGFA,PDGFA-DT
chr7	794303	794531	229	97	6.12766	14.67395	9.75694	143	NA
chr7	3294762	3294958	197	13	2.19452	5.27675	1.36922	69	SDK1-AS1
chr7	6991257	6991437	181	42	3.03294	8.6438	4.24131	15	ENSG00000287860
chr7	7255036	7255204	169	62	4.35012	10.86238	6.25183	46	LINC03016
chr7	12686667	12686869	203	220	5.1666	27.81314	22.07374	104	ARL4A
chr7	18278395	18278574	180	81	4.21845	12.87579	8.10132	74	HDAC9
chr7	20190835	20191018	184	118	5.43988	16.9548	11.87443	103	MACC1
chr7	27748082	27748252	171	95	6.22755	14.46644	9.56322	86	TAX1BP1
chr7	27764137	27764310	174	73	5.26027	12.03145	7.3279	123	TAX1BP1
chr7	28933400	28933664	265	184	5.78881	23.98663	18.4501	202	NA
chr7	29877342	29877569	228	73	4.95465	12.04479	7.3372	112	WIPF3
chr7	30988817	30989010	194	55	4.04473	10.04651	5.51034	69	GHRHR
chr7	32905419	32905687	269	91	6.87333	13.98822	9.12432	148	NA
chr7	33868180	33868388	209	95	6.41645	14.42519	9.52562	103	BBS9
chr7	46319810	46320035	226	85	5.34249	13.31768	8.50614	132	ENSG00000237760
chr7	48035375	48035646	272	113	4.28737	16.41427	11.37031	51	C7orf57
chr7	64556216	64556513	298	68	4.6039	11.51716	6.85147	218	ZNF680
chr7	64877476	64877683	208	52	4.54871	9.79173	5.27591	158	ZNF273

chr7	64910501	64910691	191	42	4.15527	8.61621	4.22012	96	ZNF273
chr7	66087055	66087249	195	79	4.94817	12.72054	7.96058	72	ASL,ENSG00000249319
chr7	74480403	74480589	187	85	3.64352	13.40923	8.59171	120	GTF2IRD1
chr7	76054316	76054484	169	45	3.95406	8.97747	4.54111	69	MDH2
chr7	77122494	77122703	210	115	3.3976	16.62714	11.56454	133	ENSG00000259628,FAM185BP,CCDC146
chr7	83037098	83037283	186	48	4.46854	9.30476	4.84531	109	PCLO
chr7	98352721	98352912	192	52	3.51353	9.7355	5.22177	104	BAIAP2L1
chr7	98376759	98376965	207	83	3.82136	13.12509	8.32598	85	BAIAP2L1
chr7	100082549	100082821	273	165	3.87539	21.93137	16.52063	119	ZNF3
chr7	101940986	101941211	226	145	5.61039	19.7905	14.51434	102	CUX1
chr7	105581350	105581621	272	159	4.76606	21.33487	15.95826	122	EFCAB10
chr7	105940121	105940382	262	62	3.85687	10.86124	6.25117	77	CDHR3
chr7	105963003	105963238	236	232	6.99492	29.07579	23.27682	116	CDHR3
chr7	106118270	106118466	197	118	6.38363	16.93104	11.85091	122	NA
chr7	113831529	113831738	210	34	4.44029	7.73126	3.43184	105	NA
chr7	127751035	127751302	268	74	4.27458	12.15924	7.44426	160	SND1
chr7	129494254	129494472	219	48	5.36507	9.37351	4.89741	145	NA
chr7	131061881	131062172	292	87	4.17232	13.60114	8.7661	147	LINC-PINT
chr7	133815935	133816160	226	21	3.1459	6.21183	2.11317	12	EXOC4
chr7	139230992	139231208	217	96	3.51755	14.51682	9.60973	90	ENSG00000228360,UBN2
chr7	140358479	140358801	323	81	4.64395	12.92183	8.14108	144	SLC37A3
chr7	141650045	141650279	235	57	3.29291	10.29271	5.73246	146	AGK
chr7	149838108	149838302	195	35	2.39511	7.85773	3.54573	167	ZNF862
chr7	151013716	151013995	280	55	4.04473	10.04651	5.51034	143	NOS3,ATG9B
chr7	151408891	151409180	290	146	4.45319	19.90908	14.62281	163	WDR86,WDR86-AS1
chr7	156488243	156488465	223	144	8.15781	19.6941	14.425	135	RNF32-DT
chr7	157397180	157397382	203	148	5.99219	20.15226	14.8472	66	DNAJB6
chr7	158818393	158818566	174	56	3.11515	10.17479	5.62294	66	ESYT2
chr8	2794681	2794867	187	69	4.94675	11.6815	6.99874	132	ENSG00000253853
chr8	8963835	8964051	217	84	4.78338	13.31016	8.49964	110	RPL10P19

chr8	11466706	11466903	198	55	3.35534	10.09402	5.55471	92	FAM167A
chr8	21948212	21948418	207	155	5.2703	20.90985	15.55546	122	XPO7
chr8	28800039	28800252	214	58	4.41801	10.42235	5.8491	116	INTS9,INTS9-AS1
chr8	30082352	30082587	236	33	2.63817	7.68034	3.38372	70	SARAF,ENSG00000272256
chr8	37881824	37882006	183	42	3.74512	8.69985	4.29263	152	RAB11FIP1
chr8	54561359	54561582	224	102	3.98616	15.15791	10.20182	97	RP1
chr8	54708161	54708436	276	87	4.17232	13.60114	8.7661	130	RP1
chr8	55346573	55346770	198	106	5.99602	15.61935	10.63956	107	XKR4
chr8	66525379	66525547	169	70	4.82857	11.74322	7.05518	80	NA
chr8	67028482	67028727	246	113	4.43619	16.38981	11.35339	93	PPP1R42
chr8	67064823	67065144	322	78	3.63953	12.6448	7.88797	147	COPS5,CSPP1
chr8	85200456	85200650	195	74	4.27458	12.15924	7.44426	92	E2F5
chr8	93754678	93754929	252	199	4.35659	25.58865	19.9634	137	TMEM67
chr8	94512802	94513142	341	65	5.18494	11.15267	6.51675	90	VIRMA
chr8	100158250	100158449	200	66	3.74005	11.25654	6.61637	109	SPAG1
chr8	102898079	102898320	242	115	4.34317	16.64104	11.57717	95	AZIN1,MAILR
chr8	120515101	120515302	202	68	4.89182	11.55409	6.8832	109	MTBP
chr8	124262812	124263055	244	81	4.21845	12.87579	8.10132	75	NA
chr8	125290611	125290856	246	82	3.93208	13.01288	8.22195	98	NSMCE2
chr8	144094734	144094981	248	107	4.19417	15.73717	10.74019	110	CYC1
chr8	144478027	144478195	169	47	3.84911	9.24109	4.78465	27	FOXH1,PPP1R16A
chr9	2560196	2560535	340	140	6.82306	19.28307	14.03582	218	VLDLR-AS1
chr9	3525658	3525968	311	155	5.2703	20.90985	15.55546	162	RFX3,ENSG00000288963,RFX3-DT
chr9	4334472	4334670	199	75	3.24697	12.24913	7.5227	105	GLIS3
chr9	15468859	15469070	212	58	5.15809	10.39223	5.82151	62	PSIP1
chr9	26956311	26956491	181	69	3.40062	11.63439	6.95575	59	IFT74,IFT74-AS1
chr9	27572362	27572671	310	174	5.12621	22.90228	17.42229	181	C9orf72
chr9	34381444	34381717	274	268	7.52284	32.78462	26.80133	136	MYORG,C9orf24
chr9	34458690	34458910	221	32	2.56513	7.49483	3.22024	131	FAM219A,DNAI1
chr9	35103263	35103515	253	89	3.46551	13.78268	8.93088	77	STOML2

chr9	42231946	42232264	319	169	7.32119	22.35946	16.91511	190	ENSG00000275649
chr9	66942543	66942762	220	125	6.33184	17.67238	12.53906	85	ENSG00000277350
chr9	68530735	68530958	224	82	4.94493	13.05854	8.26293	96	PGM5
chr9	72527510	72527760	251	134	4.17886	18.60535	13.40071	139	TMC1,ENSG00000289993
chr9	76688101	76688331	231	94	4.13373	14.33291	9.44188	87	PRUNE2,PCA3
chr9	77508561	77508758	198	101	4.8089	15.07277	10.12219	84	GNA14,GNA14-AS1
chr9	78167344	78167588	245	79	3.16986	12.6912	7.933	150	NA
chr9	84086992	84087315	324	226	7.74337	28.43185	22.66058	195	ENSG00000227463
chr9	87595732	87595951	220	134	6.39566	18.69457	13.48485	101	DAPK1
chr9	94806353	94806574	222	153	4.19401	20.73776	15.3949	101	AOPEP,MIR2278
chr9	104091225	104091411	187	55	4.68133	10.08274	5.54538	134	SMC2-DT,SMC2
chr9	105447959	105448140	182	107	4.70056	15.72927	10.73674	93	FSD1L
chr9	106955022	106955222	201	69	3.84292	11.61416	6.93771	120	ZNF462
chr9	108919590	108919774	185	91	3.72448	14.01991	9.15221	118	ELP1
chr9	109640930	109641117	188	105	5.61428	15.49877	10.52515	116	PALM2AKAP2
chr9	112308636	112308845	210	69	2.94019	11.61331	6.93771	144	PTBP3
chr9	114348909	114349134	226	183	6.1139	23.88799	18.35295	102	AKNA
chr9	114505521	114505695	175	35	2.98252	7.87747	3.56283	91	WHRN
chr9	115262449	115262647	199	69	2.94019	11.61331	6.93771	150	DELEC1
chr9	116667353	116667627	275	44	5.0554500000000004	8.83515	4.41322	147	ASTN2
chr9	122159830	122159999	170	101	4.10097	15.06948	10.12219	108	NDUFA8,MORN5
chr9	125862118	125862292	175	59	4.75547	10.57304	5.99172	100	PBX3
chr9	127706793	127707106	314	190	5.54524	24.63602	19.0609	171	PTRH1,CFAP157
chr9	127716011	127716193	183	70	3.54722	11.77674	7.08759	78	PTRH1,TTC16
chr9	128094366	128094616	251	192	4.86268	24.85635	19.27306	151	SLC25A25,ENSG00000230536
chr9	131370853	131371023	171	35	2.49639	7.87425	3.56197	30	ENSG00000285287,PRRC2B
chr9	132409908	132410106	199	81	4.06252	12.92731	8.14399	119	TTF1,CFAP77
chr9	136171447	136171665	219	94	4.07083	14.37861	9.48359	101	NA
chr9	137240285	137240546	262	149	3.66457	20.27328	14.96259	129	TUBB4B
chr9	137556181	137556349	169	48	4.09236	9.32322	4.85747	73	DPH7

chrX	10197561	10198016	456	207	7.04083	26.38529	20.7163	308	CLCN4
chrX	13791602	13791787	186	114	7.28376	16.55409	11.4956	118	GPM6B
chrX	15751438	15751687	250	78	5.72934	12.64201	7.88748	134	CA5B
chrX	24877975	24878143	169	29	3.75953	7.19911	2.9584	58	POLA1
chrX	35919488	35919850	363	76	3.83126	12.36897	7.63095	206	CFAP47
chrX	38801179	38801408	230	96	3.8995	14.51531	9.60908	89	ENSG00000287729,MID1IP1
chrX	40008769	40008941	173	57	5.27285	10.26044	5.70268	91	ENSG00000289570,ENSG00000223486
chrX	54747940	54748139	200	61	4.00742	10.77997	6.17484	119	NA
chrX	107206502	107206733	232	74	4.49414	12.18149	7.46273	63	NUP62CL,DNAAF6
chrX	107711102	107711335	234	104	6.6838	15.38734	10.41922	98	NA
chrX	117407756	117408010	255	132	7.07037	18.45565	13.27337	157	NA

Table S5. Variant allele frequency in *FOXJ1* transcripts in DCP521 and 17GM00680

				**Read number in RNAseq** (allele ratio)		
	**DNA**	**protein**	**ACMG classification**	**Ref**	**Alt**	**Gnomad 4.1 MAF (%)**
DCP521	c.867dup	p.(Ser290Glnfs*12)	5	1493 (73%)	543 (27%)	0
	c.726G>A	p.(Thr242=)	1	880 (52%)	799 (47%)	9,8
17GM00680	c.867dup	p.(Ser290Glnfs*12)	5	1119 (71%)	464 (29%)	0
	c.222C>T	p.(Pro74=)	1	813 (56%)	627 (44%)	0,08
	c.*332G>C	p.=	1	1246 (44%)	993 (56%)	11%
